# Different Anti-Vascular Endothelial Growth Factor for Patients With Diabetic Macular Edema: A Network Meta-Analysis

**DOI:** 10.3389/fphar.2022.876386

**Published:** 2022-06-23

**Authors:** Xian Wang, Xiaoning He, Fang Qi, Jia Liu, Jing Wu

**Affiliations:** ^1^ The Affiliated Hospital of Guizhou Medical University, Guiyang, China; ^2^ School of Pharmaceutical Science and Technology, Tianjin University, Tianjin, China; ^3^ Academic Department, Systematic Review Solutions Ltd, Shanghai, China

**Keywords:** diabetic macular edema, network meta-analysis, aflibercept, ranibizumab, conbercept

## Abstract

**Background:** Antiangiogenic therapy with anti-vascular endothelial growth factor (VEGF) is commonly used to treat diabetic macular edema (DME), which can reduce edema, improve vision, and prevent further visual loss. There is little head-to-head trial data to guide the selection of an individual VEGF inhibitor. Therefore, we aimed to investigate the efficacy and safety of anti-VEGF for patients with DME and to assess the differences between clinically relevant options by using network meta-analysis (NMA).

**Methods:** MEDLINE, Embase, the Cochrane Library, Web of Science, Chinese Biomedical Literature Database, Wanfang, China National Knowledge Infrastructure, and VIP databases were searched for published randomized controlled trials (RCTs) from their inception to November 2020. We included RCTs of anti-VEGF drugs (intravitreal aflibercept (IVT-AFL), intravitreal ranibizumab (IVR), and intravitreal conbercept (IVC)) treating adult patients who were diagnosed with DME, regardless of stage or duration of the disease. We estimated summary odds ratios (ORs) and mean differences (MDs) with 95% credible intervals (CrIs) using a Bayesian NMA. This study’s registration number is CRD42021259335.

**Results:** We identified 43 RCTs comprising 8,234 patients. Beneficial effects were observed in patients who used IVT-AFL compared with those who used other anti-VEGF therapies at 1-year follow-up on corrected visual acuity (BCVA) improvements (all patients: versus IVR: MD 2.83, 95% CrIs 1.64, 4.01, versus IVC: MD 2.41, 95% CrIs −0.52, 5.32; patients with worse baseline visual acuity (VA): versus IVR: MD 3.39, 95% CrIs 1.89, 4.9, versus IVC: MD 3.49, 95% CrIs 0.49, 6.44) and the proportion of patients with a gain of at least 15 Early Treatment Diabetic Retinopathy Study (ETDRS) letters (all patients: versus IVR: OR 1.55, 95% CrIs 1.11, 2.17, versus IVC: OR 2.78, 95% CrIs 1.23, 6.04; patients with worse baseline VA: versus IVR: OR 2.05, 95% CrIs 1.18, 3.58, versus IVC: OR 2.85, 95% CrIs 1.24, 6.41). The effect of improvement in BCVA was identified for IVT-AFL compared to intravitreal bevacizumab. Based on the surface under the cumulative ranking curve (SUCRA), IVT-AFL had the highest probability of being the most effective option (99.9% and 99.5% in terms of the two primary outcomes, respectively). At the 2-year follow-up, numerical differences were identified favoring IVT-AFL; however, they did not reach statistical significance when comparing IVT-AFL to IVR. In the analysis of adverse events, IVT-AFL showed a lower risk of incidence of ocular adverse events compared to other anti-VEGF therapies at 1-year follow-up (versus IVR: OR 0.45, 95% CrIs 0.28, 0.7; versus IVC: OR 0.36, 95% CrIs 0.21, 0.63).

**Conclusion:** IVT-AFL resulted in greater beneficial effects on BCVA and a higher proportion of patients with a gain of at least 15 ETDRS letters compared to IVR or IVC one year after treatment (especially in DME patients with worse baseline VA). In addition, fewer ocular adverse events occurred in the IVT-AFL group compared to the IVR or IVC groups. After two years, there was insufficient evidence to identify which anti-VEGF has superior efficacy or safety.

**Clinical Trial Registration:** https://www.crd.york.ac.uk/prospero/, PROSPERO; https://www.crd.york.ac.uk/prospero/display_record.php?ID=CRD42021259335, CRD42021259335

## Introduction

The prevalence of diabetes is rising, and consequently, the concern for the complication of diabetic retinopathy (DR) has also increased (Zheng et al., 2012; Bandello et al., 2017). Globally, the number of people with DR is estimated to grow yearly, from 126.6 million in 2010 to 191.0 million by 2030, Zheng et al. (2012). Diabetic macular edema (DME) is one of the leading causes of vision loss in the working-age population (Klein, 2007).

DME is defined as the presence of intraretinal fluid (edema) and thickening involving the macula, the portion of the retina that is responsible for central vision. It is a vision-threatening complication of diabetes and can occur at any stage or severity of DR. Edema that is centrally located within the macula can result in substantial decreases in visual acuity (VA) (Bandello et al., 2017). The therapeutic goal of DME is to improve patients’ visual function and vision-related quality of life (Jain et al., 2013). There are various treatments for DME, among them anti-vascular endothelial growth factor (VEGF) treatment, which is recommended by several clinical guidelines as a first-line treatment option by several clinical guidelines (Schmidt-Erfurth et al., 2017; Cheung et al., 2018). Anti-VEGF can be classified into the following: monoclonal antibodies to VEGF [e.g., intravitreal bevacizumab (IVB)], antibody fragments to VEGF [e.g., intravitreal ranibizumab (IVR)], and receptor fusion proteins of VEGF with the constant region of a human immunoglobulin (Ig) [e.g., intravitreal aflibercept (IVT-AFL) and intravitreal conbercept (IVC)] (Urias et al., 2017; Browning et al., 2018).

IVR is the first intraocular injection solution approved by the National Medical Products Administration (NMPA) and was approved for the DME indication in November 2018 in China. It contains an antigen-binding fragment (Fab-12), which has been widely used in DME, retinal vein occlusion, and neovascular age-related macular degeneration (nAMD). A large number of randomized controlled studies (RCTs)—such as RISE and RIDE, Protocol I, RESOLVE, and RESTORE—have demonstrated the safety and efficacy of IVR in the treatment of DME (Nguyen et al., 2012; Karst et al., 2018; Rentiya et al., 2020; Sadda et al., 2020). The recommended dose of IVR is 0.5 mg, one injection per month until best-corrected visual acuity (BCVA) is achieved. However, systemic effects observed in some clinical studies, such as hypertension, proteinuria, inhibition of bone growth, and infertility.

IVT-AFL is the first anti-VEGF that was approved by the NMPA for the treatment of DME in China (February 2018). IVT-AFL is a fusion protein comprising the second Ig domain of human VEGF receptor (VEGFR) 1, the third Ig domain of human VEGFR2, and the Fc region of human IgG1 (Cursiefen et al., 2014). VIVID and VISTA were two similarly designed randomized phase III trials that demonstrated the efficacy and safety of IVT-AFL for the treatment of patients with DME (Korobelnik et al., 2014; Brown et al., 2015). It is recommended for patients with DME with worse baseline VA at baseline (Cheung et al., 2018), and the recommended dose is 2 mg (0.05 ml) administered by intravitreal injection every 4 weeks (monthly) for the first five injections, followed by 2 mg (0.05 ml) *via* intravitreal injection once every 8 weeks (2 months).

IVB is still used off-label for the treatment of DME. Although IVB has shown a positive impact on patients with DME, it was found to be inferior to other therapies in several RCTs (Rajendram et al., 2012; Nepomuceno et al., 2013; Kriechbaum et al., 2014; Sonoda et al., 2014). In addition, significant effort is necessary to formulate IVB before it can be administered as an intravitreal injection (Diabetic Retinopathy Clinical Research Network et al., 2015).

IVC is a recombinant soluble VEGF receptor decoy. Its affinity for VEGF is 50 times that of bevacizumab and 30 times that of ranibizumab (Zhang et al., 2009). IVC was independently developed in China and was approved for the treatment of DME in 2019. The majority of the evidence for the efficacy and safety of IVC comes from retrospective studies, with similar outcomes compared to IVR (Liu and Li, 2019). In addition, one RCT (Liu et al., 2021) assessed the efficacy and safety of IVC in patients with worse baseline VA (BCVA<0.5).

Anti-VEGF therapies result in greater improvements in VA compared to other therapies (such as laser) (Virgili et al., 2018; Moisseiev and Loewenstein, 2017). Three anti-VEGF drugs (IVR, IVT-AFL, and IVC) are commonly used in clinical practice in China. To date, there are limited head-to-head comparative trials between these three anti-VEGF drugs. Previously, a network meta-analysis (NMA) was conducted to compare the efficacy and safety of these anti-VEGF drugs (Virgili et al., 2018). However, previous work has not explored the efficacy of anti-VEGF drugs on the treatment of DME in patients with worse baseline VA, nor has it investigated the long-term effects (e.g., data at the 2-year follow-up point). To provide the best current evidence to inform clinical practice, we conducted a new NMA to investigate the efficacy and safety of all available anti-VEGF drugs for DME and also assess the differences between the relevant options.

## Methods

The full protocol of this NMA has been registered on PROSPERO (registration number is CRD42021259335).

### Search Strategy

The English and Chinese databases of MEDLINE, Embase, the Cochrane Library, Web of Science, Chinese Biomedical Literature Database, Wanfang, China National Knowledge Infrastructure, and VIP were searched in literature published from inception to November 2020, without limitations on date/time, language, or document type. Search strategies for all databases are described in detail (Supplementary Appendix S1).

### Criteria for Considering Studies for This Network Meta-Analysis

We included all RCTs that met the following criteria: 1) patients with a diagnosis of DME regardless of sex, stage, or duration of the disease; 2) monotherapy of any of the following anti-VEGF drugs (DME indication approved in China), including IVT-AFL, IVR, and IVC; 3) due to insufficient direct information (i.e., head-to-head RCTs) on anti-VEGF drugs of interest, we also considered the following interventions to increase the available indirect information in the network: IVB, laser, dexamethasone implant, and placebo (sham injection or sham laser). We excluded literature whose language was not in English or Chinese, literature that was only available with an abstract but not a full-text report, or literature that was not peer reviewed. For multiple publications that were reported for the same trial or that had the same or overlapping patient groups, we only included publications with available data for targeted outcomes or those with the largest sample size.

Our primary outcomes were visual outcomes: mean change in BCVA [measure in Early Treatment Diabetic Retinopathy Study (ETDRS) letters] from baseline and the proportion of patients with a gain of at least 15 ETDRS letters (3 ETDRS lines or 0.3 logMAR). Secondary outcomes were the proportion of patients with a gain of at least 10 ETDRS letters (2 ETDRS lines or 0.2 logMAR); anatomical outcomes: mean change in central retinal thickness (CRT, μm) from baseline; the proportion of patients with complete disappearance of retinal edema after treatment (no included RCTs reported this outcome in this review); quality of life; adverse events (serious, ocular, systemic); and the number of injections. For all analyses, we recorded the outcomes at 1-year and 2-year follow-ups.

Two reviewers independently made the selections according to the titles and abstracts of the search results. Then, the full texts of the potentially included records were reviewed. Disagreements were resolved through full discussion, with assistance from a third party if necessary. The full process of study selection was presented in a PRISMA flow diagram.

### Data Extraction and Quality Assessment

Two reviewers independently extracted the following information from included studies: the information on references (author’s name, year of publication, etc.); the information on participants (country, diagnosis, sample size, age, sex, clinical stage, other important baseline clinical characteristics, criteria of inclusion, and exclusion); the information on intervention (dosage/frequency of intervention, description of intervention); and the information on outcomes (definition of the outcome, observed timepoint, and results data). Disagreements were resolved through full discussion, with assistance from a third party if necessary.

Two reviewers independently assessed the risk of bias of the included RCTs. Seven domains of the Cochrane Risk of Bias tool were evaluated, including sequence generation, allocation concealment, blinding of patients and personnel, blinding of outcome assessment, incomplete outcome data, selective outcome reporting, and other biases (Higgins et al., 2011). Disagreements were resolved through full discussion, with assistance from a third party if necessary.

Additionally, we assessed the certainty of evidence contributing to focus comparison estimates (between IVT-AFL, IVR, and IVC) of the primary outcomes (all patients at 1-year follow-up) with the Grading of Recommendations Assessment, Development, and Evaluation (GRADE) framework (Salanti et al., 2014).

### Data Analysis

We performed the Bayesian NMA using R 3.6.3 software (GeMTC package) (R Core Team, 2018). The pooled estimation and the probability for which treatment was superior were obtained using the Markov Chains Monte Carlo method. The model convergence was assessed by trace plots and Brooks–Gelman–Rubin plots (Gelman and Rubin, 1992) (Supplementary Appendices S6, S7). Dichotomous outcomes were estimated using odds ratios (ORs) and their 95% credible intervals (CrIs); continuous outcomes were estimated using the mean difference (MD) and its 95% CrIs. The cumulated ranking probabilities for all treatments were estimated and reported as the surface under the cumulative ranking curve (SUCRA). Evidence inconsistency and clinical similarities in patient characteristics and settings across trials were carefully assessed before analysis. Network geometry uses nodes to represent different interventions and edges to represent the head-to-head comparisons between interventions. The size of nodes and thickness of edges were associated with the total number of included trials for each intervention and the number of included trials including every pair of interventions, respectively. Network geometry was performed by STATA 16.0 software.

We performed an NMA according to the timepoints of follow-up, and descriptive summaries were conducted on data that was reported for less than 1 year. We planned *a priori* subgroup analyses based on the following factors:

• Worse baseline VA (defined as patients with much worse VA than 20/40);
• Previously treated (naïve versus previously treated).

When other definitions of worse baseline VA (such as VA less than 0.5 measured by decimal, VA more than 0.3 measured by logMAR, or VA less than 70 or 73 letters) were reported in the original studies, we also considered these patients as worse baseline VA. NMA was only performed on patients to have worse baseline VA. We did not analyze the other subgroup due to insufficient data.

## Results

### Study Selection

**FIGURE 1:**
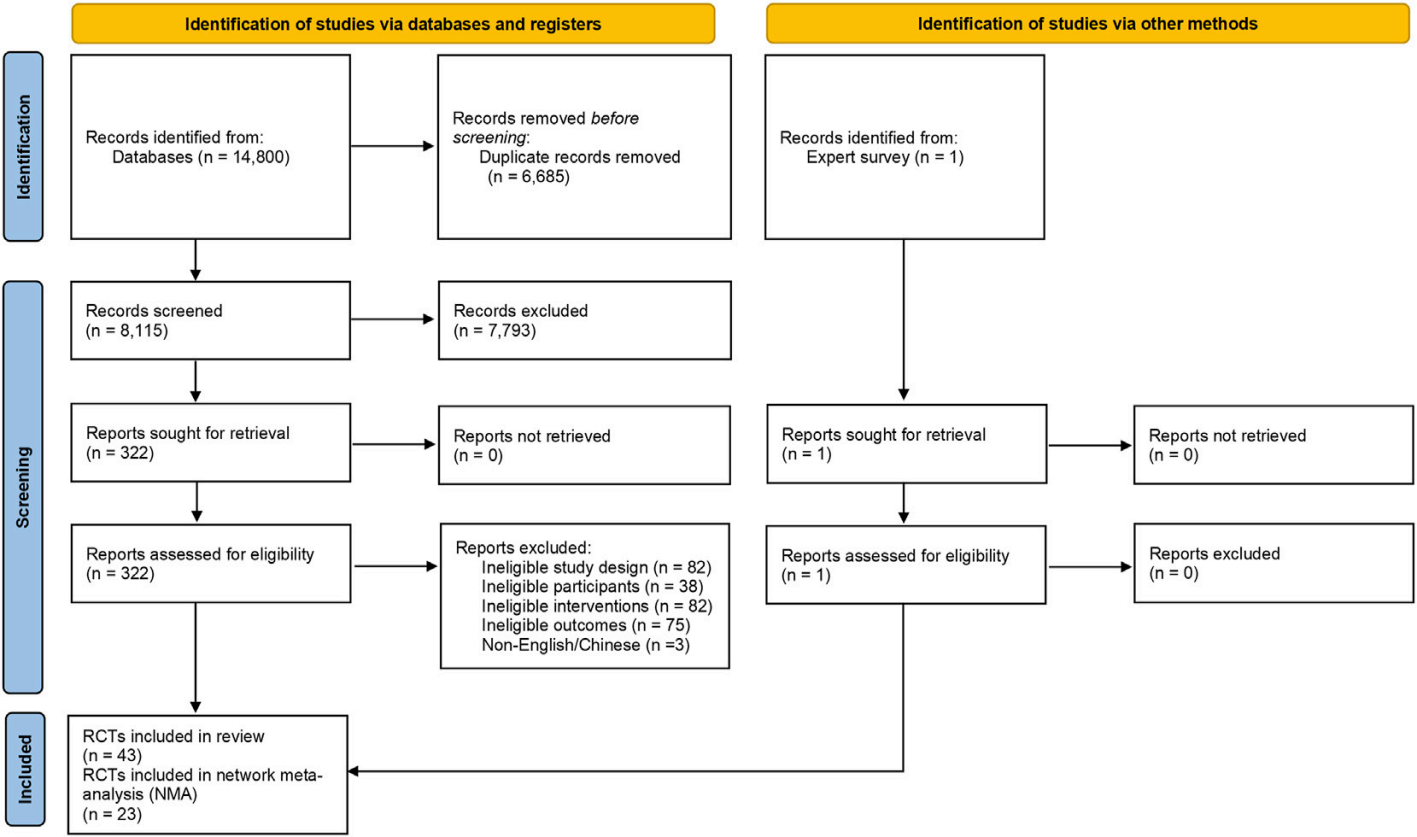
PRISMA flow diagram.

A total of 14,800 citations were identified by the search; one RCT was identified through an expert survey; and 323 potentially included records were retrieved in full text (Figure 1). Overall, 43 RCTs were included in this systematic review (references were listed in Supplementary Appendix S3), of which 23 RCTs were included in this NMA.

### Study Characteristics

The characteristics of the included studies are presented in Supplementary Appendix S2 (Supplementary Tables S1–S3). A total of 21 RCTs were multicenter trials. Overall, the RCT included in the NMA described outcomes for 5,366 patients with DME. The sample size of the included trials ranged from 18 to 1,048; the median sample size was 91.

### Risk of Bias

**FIGURE 2:**
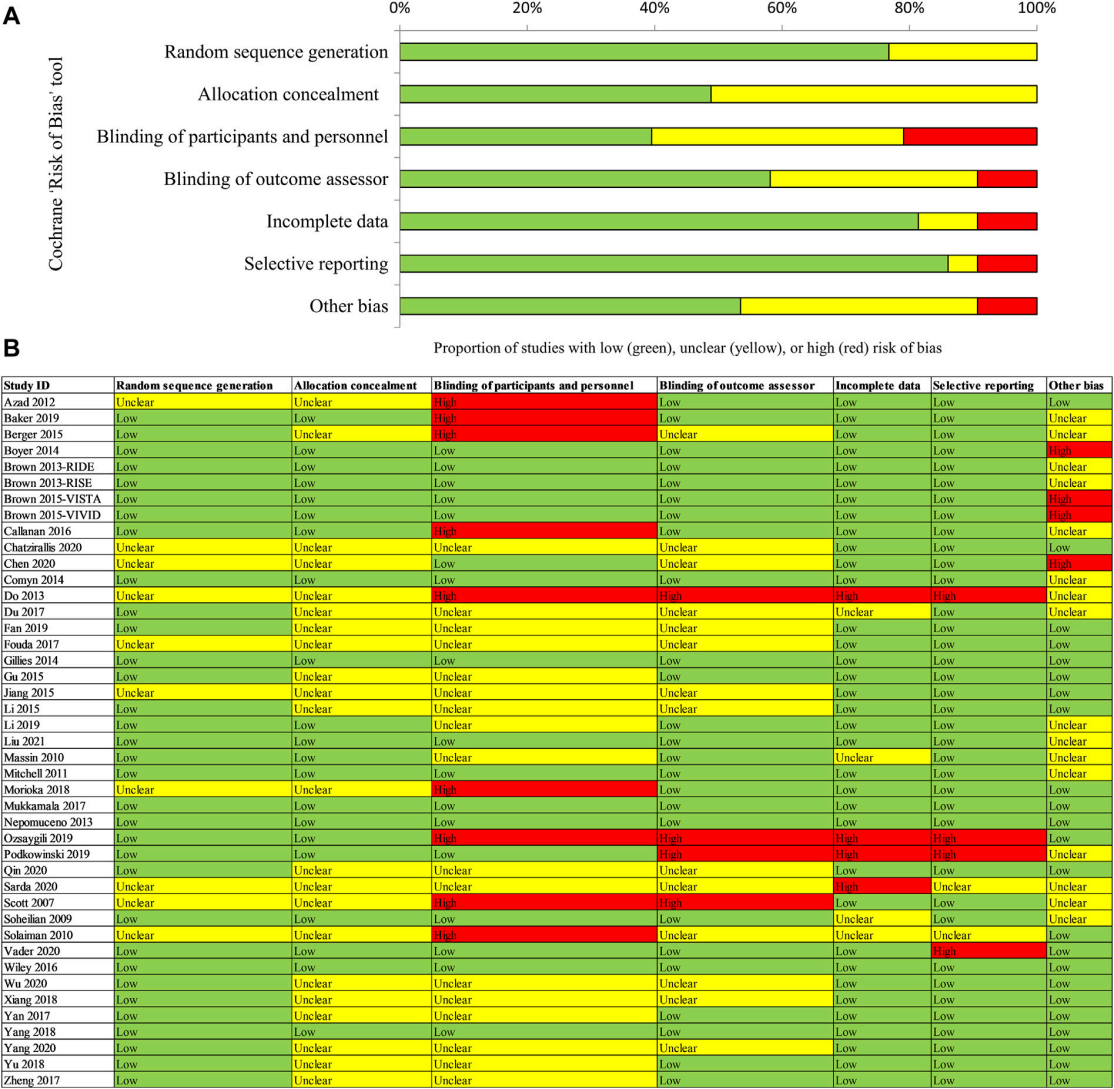
Assessment of risk of bias for included RCTs [**(A)** Risk of bias graph; **(B)** Risk of bias summary]. Notes: **(A)** Risk of bias graph: reviewers’ judgements about each risk of bias item are presented as percentages across all randomized controlled trials (RCTs). **(B)** Risk of bias summary: reviewers’ judgements about each risk of bias item for each included RCT. “Low” risk of bias in green, “Unclear” in yellow, and “High” risk of bias in red.

Figure 2A shows a detailed summary of the risk of bias in the included RCTs. For details of assessment on each domain, Figure 2B. Approximately 80% (33/43) of included trials reported adequate sequence generation and 50% (21/43) reported allocation concealment and were rated as having a low risk for selection bias; insufficient information was provided on other trials, which were rated as having unclear risk for these two domains. Approximately 10% (4/43) of included trials were open-label trials and were rated as having a high risk for performance and assessment bias. Five other trials were also rated as high risk for performance bias because the compared interventions were easily identified (e.g., anti-VEGF versus laser). About 33% (14/43) of included trials did not report information on the blinding of outcome assessment and were rated as having an unclear risk for assessment bias. Regarding attrition bias, about 10% (4/43) of included trials were rated as high risk due to a high drop-out rate (more than 20%) or an unbalance of missing data. Two included trials did not report pre-defined outcomes in the methods and were rated as high risk for reporting bias. A total of 45% (20/43) of included trials were funded by industry. The other trials were not identified as being at risk of other biases.

### Effect of Measurements

Unit of analysis issues was described in detail in Supplementary Appendix S4.

### Mean Change in Best-Corrected Visual Acuity Measured by Early Treatment Diabetic Retinopathy Study Letters from Baseline

**FIGURE 3:**
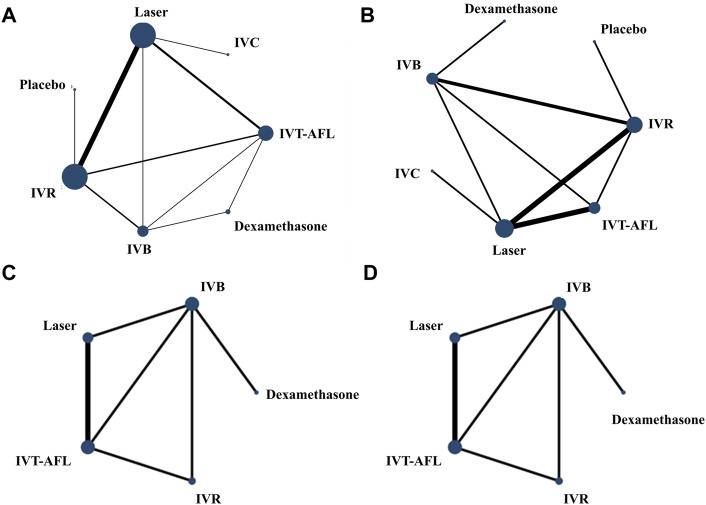
Network geometry for BCVA (ETDRS letters) mean change from baseline. All populations at 1-year follow-up [**(A)** 18 trials] and 2-year follow-up [**(C)** 5 trials]. Population with worse baseline VA at 1-year follow-up [**(B)** 12 trials] and 2-year follow-up [**(D)** 5 trials]. BCVA, best-corrected visual acuity; ETDRS, Early Treatment Diabetic Retinopathy Study; IVB, intravitreal bevacizumab; IVC, intravitreal conbercept; IVR, intravitreal ranibizumab; IVT-AFL, intravitreal aflibercept. Notes: Direct comparisons are represented by the black lines connecting the different interventions. Line width is proportional to the number of trials including every pair of interventions, whereas circle size is proportional to the total number of trials for each intervention in the network.

**FIGURE 4:**
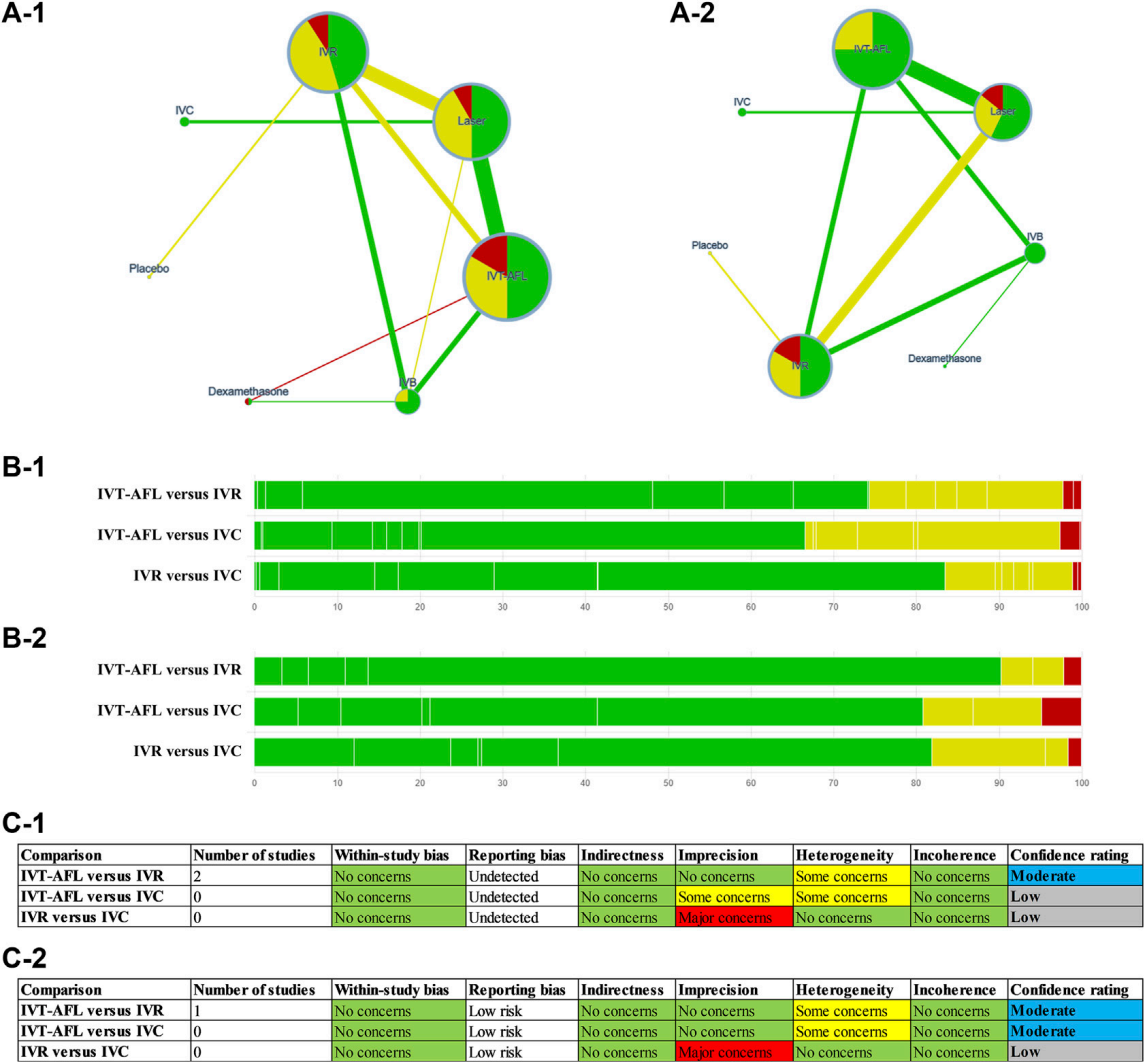
GRADE for the primary outcomes **(A-1, B-1, C-1)** are for the outcome BCVA (ETDRS letters) mean change from baseline; **(A-2, B-2, C-2)** are for the proportion of patients with a gain of at least 15 ETDRS letters. Notes: **(A)** Summary of study limitations of the included randomized controlled trials (RCTs). The colors in the circles indicate the percentage of low risk of bias RCTs (green), moderate risk of bias RCTs (yellow), and high risk of bias RCTs (red) involving each intervention. The colors of the line then indicate the summative risk of bias assessment of each comparison based on the above information–low risk of bias comparison (green), moderate risk of bias comparison (yellow), high risk of bias comparison (red). **(B)** Contribution of risk of bias comparisons to focus comparison estimates (between IVT-AFL, IVR, and IVC). **(C)** Table of domains for downgrading. BCVA, best-corrected visual acuity; ETDRS, Early Treatment Diabetic Retinopathy Study; GRADE, Grading of Recommendations Assessment, Development and Evaluation; IVC, intravitreal conbercept; IVR, intravitreal ranibizumab; IVT-AFL, intravitreal aflibercept.

Evidence networks are presented in Figure 3. Results of the NMA are reported in Table 1. The certainty of the evidence for the relative treatment effects is presented in Figure 4C-1. When compared to patients treated with other anti-VEGF therapies, patients who were treated with IVT-AFL had greater BCVA gains after treatment at 1-year follow-up [versus IVR: MD 2.83, 95% CrIs 1.64, 4.01, moderate certainty; versus IVC: MD 2.41, 95% CrIs -0.52, 5.32 (nearly reached statistical significance), low certainty; versus IVB: MD 4.56, 95% CrIs 3.16, 5.96]. Consistent improvements were also observed for IVT-AFL when compared with dexamethasone implant, laser, and placebo at 1-year follow-up. At the 2-year follow-up, greater improvements in BCVA outcomes were detected for anti-VEGF therapies compared to other therapies (dexamethasone implant/laser/placebo). No statistically significant difference was identified between IVT-AFL and IVR (MD 0.68, 95% CrIs −1.52, 2.94), but improved BCVA outcomes were detected for IVT-AFL in comparison to IVB (MD 3.17, 95% CrIs 0.87, 5.46).

**TABLE 1:** MDs with 95% CrIs of network meta-analysis for BCVA (ETDRS letters) mean change from baseline.

At 1-year follow-up						
**IVT-AFL**	***3.39*** (***1.89, 4.9***)	***3.49*** (***0.49, 6.44***)	***5.83*** (***4.03, 7.63***)	***9.12*** (***3.39, 14.81***)	***11.38*** (***10.14, 12.62***)	***15.08*** (***10.48, 19.73***)
***2.83*** (***1.64, 4.01***)	**IVR**	0.1 (−2.81, 2.99)	***2.44*** (***1.27, 3.61***)	***5.73*** (***0.2, 11.2***)	***8*** (***6.89, 9.11***)	***11.71*** (***7.33, 16.08***)
2.41 (−0.52, 5.32)	−0.41 (−3.26, 2.39)	**IVC**	2.34 (−0.7, 5.45)	5.64 (−0.62, 11.86)	***7.89*** (***5.22, 10.61***)	***11.61*** (***6.38, 16.89***)
***4.56*** (***3.16, 5.96***)	***1.73*** (***0.71, 2.76***)	2.14 (−0.81, 5.14)	**IVB**	3.28 (−2.08, 8.64)	***5.55*** (***4.02, 7.09***)	***9.26*** (***4.71, 13.8***)
***3.26*** (***1.79, 4.73***)	0.43 (−1.41, 2.28)	0.84 (−2.38, 4.12)	−1.3 (−3.24, 0.65)	**Dexamethasone implant**	2.27 (−3.3, 7.87)	5.99 (−1.13, 12.96)
***10.3*** (***9.18, 11.41***)	***7.47*** (***6.65, 8.29***)	***7.88*** (***5.2, 10.62***)	***5.74*** (***4.48, 6.99***)	***7.04*** (***5.22, 8.85***)	**Laser**	3.72 (−0.78, 8.22)
***14.54*** (***10.03, 19.05***)	***11.72*** (***7.34, 16.07***)	***12.13*** (***6.88, 17.28***)	***9.99*** (***5.47, 14.45***)	***11.28*** (***6.55, 16.01***)	4.25 (−0.21, 8.68)	**Placebo**
**At 2-year follow-up**						
**IVT-AFL**	2.2 (−1.32, 5.72)	**-**	***5.21*** (***1.82, 8.59***)	3.7 (−0.98, 8.39)	***9.93*** (***8.2, 11.66***)	**-**
0.70 (−1.53, 2.92)	**IVR**	**-**	3.01 (−0.55, 6.52)	1.5 (−3.35, 6.3)	***7.73*** (***3.88, 11.59***)	**-**
**-**	**-**	**IVC**	**-**	**-**	**-**	**-**
***3.19*** (***0.93, 5.45***)	2.48 (0.24, 4.71)	**-**	**IVB**	−1.5 (−4.77, 1.74)	***4.72*** (***1.1, 8.3***)	**-**
***5.68*** (***0.05, 11.31***)	5.02 (−0.61, 10.61)	**-**	2.53 (−2.63, 7.61)	**Dexamethasone implant**	***6.23*** (***1.37, 11.08***)	**-**
***9.81*** (***8.06, 11.53***)	***9.13*** (***6.35, 11.85***)	**-**	***6.64*** (***3.9, 9.39***)	4.11 (−1.72, 9.94)	**Laser**	**-**
**-**	**-**	**-**	**-**	**-**	**-**	**Placebo**

Results of the network meta-analysis for all the population were in the lower triangle, and the estimation was calculated as the treatment in column compared with the treatment in row. MDs higher than 0 favor the treatment in the column. Results of the network meta-analysis for the population with worse baseline VA were in the upper triangle, and the estimation was calculated as the treatment in the row compared with the treatment in the column. MDs higher than 0 favor treatment in row. Statistically, significance was presented in bold italic format. To obtain MDs for comparisons in the opposite direction, negatives should be taken.

BCVA, best-corrected visual acuity; CrI, credible interval; ETDRS, early treatment diabetic retinopathy study; IVB, intravitreal bevacizumab; IVC, intravitreal conbercept; IVR, intravitreal ranibizumab; IVT-AFL, intravitreal aflibercept; MD, mean difference.

The analysis of the subgroup of patients with worse baseline VA also concluded that patients receiving IVT-AFL experienced greater BCVA gains after 1-year follow-up compared to patients receiving other anti-VEGF therapies (versus IVR: MD 3.39, 95% CrIs 1.89, 4.9; versus IVC: MD 3.49, 95% CrIs 0.49, 6.44; versus IVB: MD 5.83, 95% CrIs 4.03, 7.63). At the 2-year follow-up, there were improved BCVA outcomes for anti-VEGF therapies versus laser (MD 9.93, 95% CrIs 8.2, 11.66). No statistically significant difference was identified for IVT-AFL versus IVR (MD 2.2, 95% CrIs −1.32, 5.72). In addition, an improvement effect for BCVA was detected for IVT-AFL compared to IVB (MD 3.17, 95% CrIs 0.87, 5.46) for the patients with worse baseline VA at the 2-year follow-up.

Results of SUCRA showed that IVT-AFL had a chance of 99% and approximately 95% (at the 1-year and 2-year follow-ups, respectively) of being the anti-VEGF treatment associated with the greatest improvement in BCVA and that laser or placebo was associated with the worst BCVA improvement when compared to all other therapies (Table 2).

**TABLE 2:** Ranking probabilities for all interventions for BCVA (ETDRS letters) mean change from baseline.

At 1-year follow-up				
Interventions	All patients		Patients with worse baseline VA	
	Ranks	SUCRA	Ranks	SUCRA
*IVT-AFL*	1	0.9910915	1	0.9980333
*IVR*	3	0.6776878	2	0.7510292
*IVC*	2	0.7133875	3	0.7293625
*IVB*	5	0.4194051	4	0.4918083
*Dexamethasone implant*	4	0.3624541	5	0.3191792
*Laser*	6	0.1616045	6	0.1935250
*Placebo*	7	0.0051457	7	0.0170625

**At 2-year follow-up**				
**Interventions**	**All patients**		**Patients with worse baseline VA**	
	**Ranks**	**SUCRA**	**Ranks**	**SUCRA**
*IVT-AFL*	1	0.9249188	1	0.9563687
*IVR*	2	0.8048500	2	0.6975625
*IVB*	3	0.4621562	4	0.3069875
*Dexamethasone implant*	4	0.2873125	3	0.5363687
*Laser*	5	0.0207625	5	0.0027125

BCVA, best-corrected visual acuity; ETDRS, early treatment diabetic retinopathy study; IVB, intravitreal bevacizumab; IVC, intravitreal conbercept; IVR, intravitreal ranibizumab; IVT-AFL, intravitreal aflibercept; SUCRA, the surface under the cumulative ranking curve; VA, visual acuity.

### The Proportion of Patients With a Gain of at Least 15 Early Treatment Diabetic Retinopathy Study Letters

**FIGURE 5:**
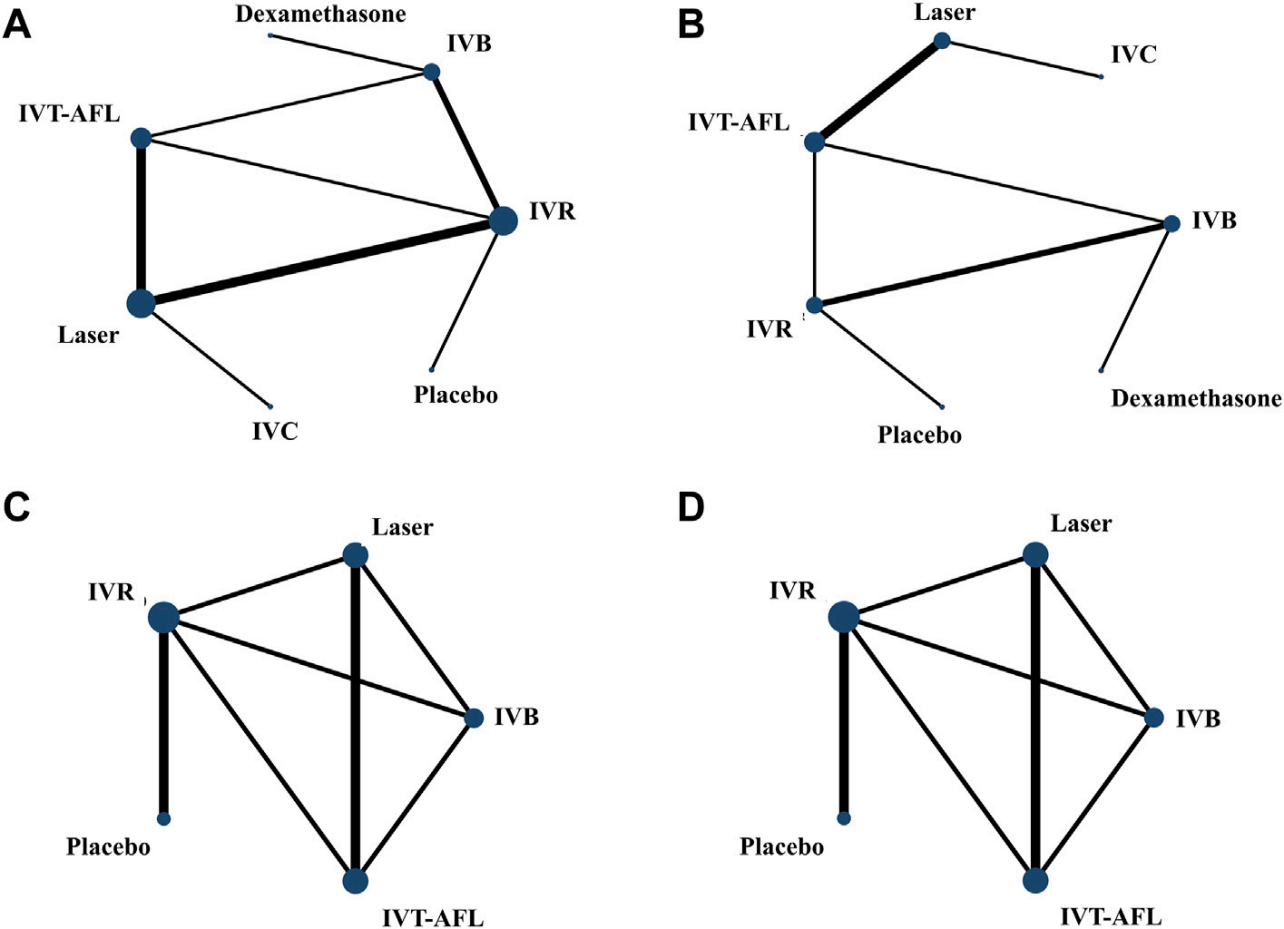
Network geometry for the proportion of patients with at least 15 ETDRS letters. All populations at 1-year follow-up [**(A)** 11 trials] and 2-year follow-up [**(C)** 7 trials]. Population with worse baseline VA at 1-year follow-up [**(B)** 8 trials] and 2-year follow-up [**(D)** 7 trials]. ETDRS, Early Treatment Diabetic Retinopathy Study; IVB, intravitreal bevacizumab; IVC, intravitreal conbercept; IVR, intravitreal ranibizumab; IVT-AFL, intravitreal aflibercept. Notes: Direct comparisons are represented by the black lines connecting the different interventions. Line width is proportional to the number of trials including every pair of interventions, whereas circle size is proportional to the total number of trials for each intervention in the network.

Evidence networks are presented in Figure 5. Results of the NMA are reported in Table 3. The certainty of the evidence for the relative treatment effects is presented in Figure 4C-2. When compared to other anti-VEGF therapies, more patients using IVT-AFL gained at least 15 letters after treatment at 1-year follow-up (versus IVR: OR 1.55, 95% CrIs 1.11, 2.17, moderate certainty; versus IVC: OR 2.78, 95% CrIs 1.23, 6.04, moderate certainty; versus IVB: OR 1.85, 95% CrIs 1.26, 2.73). Consistently, IVT-AFL showed beneficial effects on this outcome compared to dexamethasone implant, laser, and placebo at 1-year follow-up. At the 2-year follow-up, patients treated with anti-VEGF showed better outcomes when compared to patients treated with laser/placebo. No statistically significant differences were identified among patients treated with the different anti-VEGF therapies (IVT-AFL versus IVR: OR 1.18, 95% CrIs 0.8, 1.73; IVT-AFL versus IVB: OR 1.38, 95% CrIs 0.95, 2.01).

**TABLE 3:** ORs with 95% CrIs of network meta-analysis for the proportion of patients with a gain of at least 15 ETDRS letters.

At 1-year follow-up						
**IVT-AFL**	***2.05*** (***1.18, 3.58***)	***2.85*** (***1.24, 6.41***)	***2.89*** (***1.67, 5.08***)	***4.72*** (***1.56, 14.47***)	***5.48*** (***3.87, 7.97***)	***9.16*** (***2.99, 32.83***)
***1.55*** (***1.11, 2.17***)	**IVR**	1.4 (0.5, 3.74)	1.41 (0.86, 2.32)	2.31 (0.78, 6.89)	***2.69*** (***1.37, 5.23***)	***4.44*** (***1.71, 14.23***)
***2.78*** (***1.23, 6.04***)	1.79 (0.77, 4.03)	**IVC**	1.01 (0.38, 2.77)	1.66 (0.41, 6.71)	1.93 (0.94, 4.11)	3.23 (0.79, 14.85)
***1.85*** (***1.26, 2.73***)	1.19 (0.82, 1.75)	0.67 (0.28, 1.61)	**IVB**	1.63 (0.62, 4.38)	1.9 (0.98, 3.7)	***3.15*** (***1.08, 11.07***)
***3.03*** (***1.06, 8.71***)	1.95 (0.69, 5.57)	1.1 (0.3, 4.12)	1.63 (0.62, 4.41)	**Dexamethasone implant**	1.17 (0.36, 3.78)	1.95 (0.45, 9.29)
***5.35*** (***3.93, 7.41***)	***3.45*** (***2.39, 5.09***)	1.93 (0.94, 4.1)	***2.89*** (***1.83, 4.63***)	1.77 (0.6, 5.19)	**Laser**	1.66 (0.51, 6.25)
***6.9*** (***2.51, 22.69***)	***4.43*** (***1.72, 13.93***)	2.51 (0.7, 10.25)	***3.73*** (***1.33, 12.46***)	2.3 (0.54, 10.73)	1.29 (0.46, 4.29)	**Placebo**
**At 2-year follow-up**						
**IVT-AFL**	1.1 (0.64, 1.87)	-	1.41 (0.86, 2.29)	-	***3.72*** (***2.63, 5.37***)	***4.28*** (***2.22, 8.32***)
1.18 (0.8, 1.73)	**IVR**	-	1.28 (0.74, 2.22)	-	***3.4*** (***1.88, 6.19***)	***3.89*** (***2.67, 5.82***)
-	-	**IVC**	-	-	-	-
1.38 (0.95, 2.01)	1.17 (0.78, 1.75)	-	**IVB**	-	***2.64*** (***1.57, 4.55***)	***3.03*** (***1.56, 5.97***)
***3.72*** (***2.64, 5.32***)	***3.16*** (***1.94, 5.19***)	-	***2.7*** (***1.71, 4.32***)	**Dexamethasone implant**	-	-
-	-	-	-	-	**Laser**	1.15 (0.56, 2.33)
***4.6*** (***2.67, 8***)	***3.89*** (***2.66, 5.83***)	-	***3.34*** (***1.92, 5.85***)	-	1.23 (0.66, 2.32)	**Placebo**

Results of the network meta-analysis for all the population were in the lower triangle, and the estimation was calculated as the treatment in column compared with the treatment in row. ORs higher than 1 favor the treatment in the column. Results of the network meta-analysis for the population with worse baseline VA were in the upper triangle, and the estimation was calculated as the treatment in the row compared with the treatment in the column. ORs higher than 1 favor the treatment in row. Statistically, significance was presented in bold italic format. To obtain ORs for comparisons in the opposite direction, reciprocals should be taken.

CrI, credible interval; ETDRS, early treatment diabetic retinopathy study; IVB, intravitreal bevacizumab; IVC, intravitreal conbercept; IVR, intravitreal ranibizumab; IVT-AFL, intravitreal aflibercept; OR, odds ratio.

Similar results were obtained for patients with worse baseline VA. More patients receiving IVT-AFL gained at least 15 letters after treatment at 1-year follow-up compared to those receiving other anti-VEGF therapies (versus IVR: OR 2.05, 95% CrIs 1.18, 3.58; versus IVC: OR 2.85, 95% CrIs 1.24, 6.41; versus IVB: OR 2.89, 95% CrIs 1.67, 5.08); beneficial effects on this outcome were also detected for IVT-AFL compared to dexamethasone implant, laser, or placebo. At the 2-year follow-up, improved BCVA effects were detected for anti-VEGF therapies in comparison to other therapies (laser/placebo); no statistically significant differences were identified among the different anti-VEGF therapies (IVT-AFL versus IVR: OR 1.1, 95% CrIs 0.64, 1.87; IVT-AFL versus IVB: OR 1.41, 95% CrIs 0.86, 2.29; IVR versus IVB: OR 1.28, CrIs 0.74, 2.22).

Results of SUCRA showed that IVT-AFL had a chance of 99% and approximately 90% (at the 1-year and 2-year follow-ups, respectively) of being the anti-VEGF treatment associated with the best visual outcome and that laser or placebo was associated with the lowest proportions of patients gaining at least 15 letters (Table 4) when compared to other therapies.

**TABLE 4:** Ranking probabilities for all interventions for the proportion of patients with a gain of at least 15 ETDRS letters.

At 1-year follow-up				
Interventions	All patients		Patients with worse baseline VA	
	Ranks	SUCRA	Ranks	SUCRA
*IVT-AFL*	1	0.99460833	1	0.99740417
*IVR*	2	0.77286250	2	0.76619167
*IVC*	4	0.45182083	3	0.57498333
*IVB*	3	0.63882917	4	0.56081250
*Dexamethasone implant*	5	0.40756667	5	0.31252917
*Laser*	6	0.14410417	6	0.21077083
*Placebo*	7	0.09020833	7	0.07730833

**At 2-year follow-up**				
**Interventions**	**All patients**		**Patients with worse baseline VA**	
	**Ranks**	**SUCRA**	**Ranks**	**SUCRA**
*IVT-AFL*	1	0.938750	1	0.8875875
*IVR*	2	0.743794	2	0.7948312
*IVB*	3	0.567450	3	0.5673750
*Laser*	4	0.186694	4	0.1619687
*Placebo*	5	0.063313	5	0.0882375

ETDRS, early treatment diabetic retinopathy study; IVB, intravitreal bevacizumab; IVC, intravitreal conbercept; IVR, intravitreal ranibizumab; IVT-AFL, intravitreal aflibercept; SUCRA, the surface under the cumulative ranking curve; VA, visual acuity.

### The Proportion of Patients With a Gain of at Least 10 Early Treatment Diabetic Retinopathy Study Letters

Twelve trials reported 1-year follow-up results and five trials reported 2-year follow-up data for the proportion of patients with a gain of at least 10 ETDRS letters. Results indicated that there was no statistically significant difference in outcomes between IVT-AFL and other anti-VEGF therapies (IVR, IVC, or IVB) at either 1-year follow-up or 2-year follow-up. By contrast, beneficial effects of IVT-AFL were observed compared with laser at both 1-year and 2-year follow-ups (Supplementary Appendix S5).

In a subgroup analysis of patients with worse baseline VA, patients receiving IVT-AFL showed more BCVA improvements after 1-year follow-up compared with patients with other anti-VEGF therapies (versus IVC: OR 2.26, 95% CrIs 1.17, 4.33; versus IVB: MD 2.29, 95% CrIs 1.26, 4.24). In the subgroup of patients with lower baseline VA, IVT-AFL performed consistently better than laser (Supplementary Appendix S5).

### Anatomical Outcome: Mean Change in Central Retinal Thickness (μm) from Baseline

Seventeen trials reported 1-year follow-up results and four trials reported 2-year follow-up data for anatomical outcomes. IVT-AFL was associated with greater improvements in CRT thickness compared with other therapies, except for the comparison between IVT-AFL and IVR or IVC at the 1-year follow-up, where no statistically significant difference was observed. At the 2-year follow-up, no statistically significant differences in anatomical outcomes were identified for all comparisons except for that between IVT-AFL and IVB. Furthermore, results from patients with worse baseline VA indicated that at 1 year, the CRT of those in the IVT-AFL group decreased more than that in the other therapies after treatment (except for the comparison between IVT-AFL and IVC, Supplementary Appendix S5). We did not identify sufficient data for subgroup analysis at the 2-year follow-up.

### Quality of Life

Five trials reported 1-year follow-up results and one trial reported around 2-year follow-up (100 weeks) data for this outcome assessed by the National Eye Institute Visual Function Questionnaire-25 item (NEI VFQ-25). NMA was not performed due to insufficient data, and the results of every single trial are presented in Table 5. The quality of life improved significantly in the anti-VEGF groups (IVT-AFL, IVR, or IVC) when compared with the laser group at 1-year follow-up. At the 2-year follow-up, no clear differences were identified for all comparisons.

**TABLE 5:** Quality of life assessed by NEI VFQ-25, high = well).

IVT-AFL (2mg, bi-monthly) versus Laser						
Item	Follow-up	Study ID	Sample Size	MD	95% CI	*p*-Value
*Subscale (near activities) score*	1-year	Chen 2020	251	***6.87***	***0.48 to 13.26***	***0.04***
	100-week	Brown 2015-VISTA	305	*4.7*	*−0.18 to 9.58*	0.08*
		Brown 2015-VIVID	267	2.2	Not reported	>0.05**
*Subscale (distance activities) score*	1-year	Chen 2020	251	***8.01***	***2.36 to 13.66***	***0.005***
	100-week	Brown 2015-VISTA	305	2.4	Not reported	>0.05**
		Brown 2015-VIVID	267	2.7	Not reported	>0.05**
**IVT-AFL (2mg, monthly) versus Laser**						
**Item**	**Follow-up**	**Study ID**	**Sample Size**	**MD**	**95% CI**	***p*** **-Value**
*Subscale (near activities) score*	1-year	Chen 2020	251	*5.78*	*−0.71 to 12.27*	0.08*
	100-week	Brown 2015-VISTA	308	2.8	Not reported	>0.05**
		Brown 2015-VIVID	268	3.4	Not reported	>0.05**
*Subscale (distance activities) score*	1-year	Chen 2020	251	2.82	*−*3.08 to 8.72	0.35
	100-week	Brown 2015-VISTA	308	*4.8*	*0.05 to 9.55*	0.05*
		Brown 2015-VIVID	268	2.4	Not reported	>0.05**
**IVR (0.5mg, 3+PRN) versus Laser**						
**Item**	**Follow-up**	**Study ID**	**Sample Size**	**MD**	**95% CI**	***p*** **-Value**
*Composite score*	1-year	Berger 2015	147	***6***	***2.7 to 9.4***	***<0.001***
		Mitchell 2011	227	***4.4***	Not reported	***0.014***
*Subscale (general vision) score*		Mitchell 2011	227	***7.8***	Not reported	***<0.001***
*Subscale (near activities) score*		Mitchell 2011	227	***7.9***	Not reported	***<0.001***
*Subscale (distance activities) score*		Mitchell 2011	227	***4.9***	Not reported	***<0.001***
**IVC (0.5mg, 1+PRN) versus Laser**						
**Item**	**Follow-up**	**Study ID**	**Sample Size**	**MD**	**95% CI**	***p*** **-Value**
*Composite score*	1-year	Liu 2021	248	***8.1***	***3.47 to 12.73***	***0.0006***

MD was calculated using RevMan 5.4 when necessary. *Significant differences were identified in the original trials. **No significant differences were identified in the original trials.

Notes: 1) general vision was defined as eyesight now (with glasses or contact lenses, if wear them). Near activities are defined as reading ordinary print in newspapers, performing work or hobbies requiring near vision, or finding something on a crowded shelf. Distance activities are defined as reading street signs or names on stores, and going downstairs, steps, or curbs. 2) 12 months or 52 weeks were considered as one year. Statistically, significance was presented in bold italic format. CI, confidence interval; IVB, intravitreal bevacizumab; IVC, intravitreal conbercept; IVR, intravitreal ranibizumab; IVT-AFL, intravitreal aflibercept; MD, mean difference; NEI VFQ-25, National Eye Institute Visual Function Questionnaire-25 item; PRN, pro re nata.

### Adverse Events

As insufficient data was identified for the 2-year follow-up, analysis of adverse events was only performed for the 1-year follow-up. At the 1-year follow-up No statistically significant difference was observed between IVT-AFL and other anti-VEGF therapies for the proportion of patients with serious adverse events (versus IVR: OR 0.93, 95% CrIs 0.66, 1.33; versus IVC: OR 1.11, 95% CrIs 0.52, 2.39) and a lower risk of incidence of ocular adverse events (mainly including intraocular pressure increases, intraocular hypertension, conjunctival or subconjunctival hemorrhage, eye pain) was found in the IVT-AFL group [versus IVR: OR 0.45, 95% CrIs 0.28, 0.7, assumed risk in the IVR group was 31.3%; versus IVC: OR 0.36, 95% CrIs 0.21, 0.63, assumed risk in IVC group was 57.6% (assumed risk in the control group was estimated as the sum of the events divided by the sum of the participants of included studies in the NMA of this outcome)]. No statistically significant differences were observed between IVT-AFL and dexamethasone implant, laser, and placebo for serious adverse events. A lower proportion of patients experienced ocular adverse events in the IVT-AFL group compared with the dexamethasone implant and laser groups (Supplementary Appendix S5). In addition, the incidence of systemic adverse events reported in included trials is summarized in Table 6. The incidence was around 63% in both the IVT-AFL and IVR groups in patients with worse baseline VA at 1-year follow-up. For a 2-year follow-up, the incidence of systemic adverse events in the IVT-AFL group was 5.36%, and 7.8% and 11.93% in the IVR and IVC groups, respectively.

**TABLE 6:** Incidence of systemic adverse events.

Study ID	Treatment	No. of participants with systemic adverse events	Sample Size	Incidence (%)
Patients with worse baseline VA at 1-year follow-up				
Chen 2020	IVT-AFL (2 mg, monthly)	79	127	62.20
Chen 2020	IVT-AFL (2 mg, bi-monthly)	81	127	63.78
Chen 2020	Laser (1 + PRN)	77	124	62.10
Li 2015	IVR (0.5 mg*)	0	34	0.00
Li 2015	Laser	0	34	0.00
Massin 2010	IVR (0.3 mg, 3 + PRN)	32	51	62.75
Massin 2010	IVR (0.5 mg, 3 + PRN)	32	51	62.75
Massin 2010	Placebo	32	49	65.31
All patients at 2-year follow-up				
Mukkamala 2017	IVT-AFL (2 mg, monthly or 1 + PRN)	12	224	5.36
Mukkamala 2017	IVB (1.25 mg, monthly or 1 + PRN)	17	218	7.80
Mukkamala 2017	IVR (0.5 mg, monthly or 1 + PRN)	26	218	11.93

*Frequency of IVR was not reported.

IVB, intravitreal bevacizumab; IVC, intravitreal conbercept; IVR, intravitreal ranibizumab; IVT-AFL, intravitreal aflibercept; No., number; PRN, pro re nata; VA, visual acuity.

### Number of Injections

Due to differences in the treatment regimens used in the included trials, we described the number of injections as an outcome without using statistical tests. Regarding the included trials in the primary outcome (BCVA mean change from baseline) at the 1-year follow-up, the mean number of IVT-AFL injections was 7.52, and 7.77 and, 9.5 injections of IVR and IVC, respectively (Table 7).

**TABLE 7:** Mean number of injections at 1-year follow-up.

Mean number of injections of included studies in the below outcomes	Mean number of injections		
	*IVT-AFL*	*IVR*	IVC*
*BCVA mean change from baseline (measured by ETDRS letters)*	7.52	7.77	9.5
*The proportion of patients with a gain of at least 15 ETDRS letters*	8.88	8.93	9.5

Bi-monthly or PRN (pro re nata) regimens were used in included trials. *Data was identified from only one RCT (Liu 2021, conbercept PRN).

BCVA, best-corrected visual acuity; ETDRS, early treatment diabetic retinopathy study; IVC, intravitreal conbercept; IVR, intravitreal ranibizumab; IVT-AFL, intravitreal aflibercept.

## Discussion

The systematic literature review identified 43 RCTs that reported outcomes from 8,234 patients. Of the 43 RCTs identified in the literature review, 21 RCTs contributed to the NMA (corresponding to 5,366 patients). Analysis of the two primary outcomes showed that patients receiving IVT-AFL experienced improved BCVA gains (a mean increase of 2.62 letters in all patients and a mean increase of 3.44 letters in the patients with worse baseline VA) and were more likely to gain at least 15 ETDRS letters compared with other anti-VEGF therapies (IVR or IVC) at 1 year after treatment. IVT-AFL had the highest probability of being the most effective option (99.9% and 99.5% in terms of the two primary outcomes, respectively). At the 2-year follow-up, when IVT-IVA was compared to IVR, numerical differences were identified (a mean increase of 0.7 letters in all patients, a mean increase of 2.2 letters in patients with worse baseline VA, and may increase the 10% of patients with a gain of at least 15 ETDRS letters), and the SUCRA results showed that IVT-AFL may be preferred over other anti-VEGF therapies in DME treatment (92.4% and 93.8% in terms of the two primary outcomes, respectively); however, they did not reach statistical significance. In addition, no published IVC RCT was found for patients with DME at 2-year follow-up as part of our search strategy.

Similar results were found in secondary outcomes in all patients. Although numerical differences were identified between IVT-AFL and IVR, they did not reach statistical significance in the proportion of patients with a gain of at least 10 ETDRS letters, or change in CRT in all patients. In patients with worse baseline VA receiving IVT-AFL, they were more likely to gain at least 10 ETDRS letters compared with IVC 1 year after treatment. And the CRT of those patients with worse baseline VA in the IVT-AFL group decreased more than that in the IVR group.

For safety outcomes, IVT-AFL showed a lower risk of incidence for ocular adverse events at the 1-year follow-up when compared with IVR or IVC. For every 1000 patients treated with IVR, 144 fewer would experience ocular adverse events if treated with IVT-AFL, and for every 1000 patients treated with IVC, 248 fewer would experience ocular adverse events if treated with IVT-AFL. No clear difference was identified in serious events at the 1-year follow-up among anti-VEGF comparisons.

This review confirms the findings from a previous review (Virgili et al., 2018) that IVT-AFL confers benefits over IVR at 1 year. Our review included studies with a broad range of characteristics in DME patients and included the latest evidence of IVC in NMA. The subgroup analysis results for patients with worse baseline VA were consistent with results for all patients, which indicates comprehensive applicability of the findings to all DME patients. Moreover, it is noteworthy that all trials included in this review were RCTs, and real-world data from observational trials was not included in our review. It has been reported that different safety results may be observed for patients with DME in real-world studies compared with those in RCTs (Ziemssen et al., 2017).

Overall, 62% of the included trials were rated as having a low or unclear risk of bias for all domains. Such uncertainty was mainly because the authors of the included trials did not provide sufficient information for reviewers to make a judgment on specific domains. We graded the certainty of the evidence for mixed estimates of primary outcomes (Figure 4). No important issue was identified for the main comparison (IVT-AFL versus IVR; IVT-AFL versus IVC). In addition, we only included published data, so there is a possibility of publication bias.

Our findings are consistent with the previous NMA that was published in 2018, reporting significant beneficial effects for patients receiving IVT-AFL compared with IVR 1 year after treatment (Virgili et al., 2018). Unlike the 2018 review, here we focused on the three major types of anti-VEGF therapies that have been approved in China and included indirect evidence between anti-VEGF and steroids (such as dexamethasone implant) to supplement the analysis. Furthermore, analyses were performed on 2-year data in this NMA.

Recently published guidelines show that IVT-AFL has superior efficacy when compared with intravitreal ranibizumab at the 1-year follow-up and IVB at both the 1- and 2-year follow-ups. Although IVT-AFL and IVR are the drugs of choice for a BCVA ETDRS letter score of less than 69, all three medications are equivalent in improving VA with a baseline BCVA of 69 or more letters (Diabetic Retinopathy Clinical Research Network et al., 2015; Schmidt-Erfurth et al., 2017). Our review provided supportive evidence for the superiority of IVT-AFL over IVR in the 1-year follow-up period, especially in DME patients with worse baseline VA (Snellen equivalent, 20/40 or worse).

## Conclusion

With moderate certainty in evidence, IVT-AFL confers clear beneficial effects in improving visual function in patients with DME compared with IVR or IVC one year after treatment, in both an all-patient group and in patients with worse baseline VA. Two years after treatment, IVT-AFL showed more beneficial effects compared to IVB, but no clear differences were identified when compared to IVR. Similar conclusions were reached in the patients with worse baseline VA as in the all-patient group. In addition, our findings showed that IVT-AFL had a lower risk of incidence of ocular adverse events compared to IVR or IVC.

For future research, more primary studies that compare the 2-year outcomes of these anti-VEGF agents are needed. In addition, more direct head-to-head trials between these anti-VEGF agents are needed to provide accurate, direct evidence to guide clinical practice. More trials comparing IVC and other anti-VEGF agents with more than 1-year follow-up are needed to provide comprehensive knowledge on anti-VEGF agents for patients with DME.

## Data Availability

The original contributions presented in the study are included in the article/Supplementary Materials; further inquiries can be directed to the corresponding author.
