# Supplementary material for: Different Anti-Vascular Endothelial Growth Factor for Patients With Diabetic Macular Edema: A Network Meta-Analysis
Source: Front Pharmacol. 2022 Jun 23;13:876386. doi: 10.3389/fphar.2022.876386 (PMC9260109; doi:10.3389/fphar.2022.876386)
Supplement: Supplementary file 1 [file DataSheet1.docx]

**ONLINE APPENDIX**

Index

[Appendix 1 Search strategies 3](#_Toc102919573)

[MEDLINE 3](#_Toc102919574)

[Embase 3](#_Toc102919575)

[Cochrane library 4](#_Toc102919576)

[Web of Science 5](#_Toc102919577)

[CBM 5](#_Toc102919578)

[Wanfang 6](#_Toc102919579)

[CNKI 6](#_Toc102919580)

[VIP 6](#_Toc102919581)

[Appendix 2 Characteristics of included RCTs 8](#_Toc102919582)

[Table S1 Characteristics of Study and Population 8](#_Toc102919583)

[Table S2 Inclusion and Exclusion of included RCTs 12](#_Toc102919584)

[Table S3 Characteristics of Interventions 18](#_Toc102919585)

[Appendix 3 Reference list of included RCTs (Citations for studies in Table 1) 22](#_Toc102919586)

[Appendix 4 Unit of analysis issues 24](#_Toc102919587)

[Mean change in best-corrected visual acuity (letters) from baseline 24](#_Toc102919588)

[Mean change in central retinal thickness (μm) from baseline 24](#_Toc102919589)

[Appendix 5 Results of network meta-analyses (NMAs) for secondary outcomes 25](#_Toc102919590)

[The Proportion of patients with a gain of at least 10 Early Treatment Diabetic Retinopathy Study (ETDRS) letters (2 ETDRS lines or 0.2 logMAR) 25](#_Toc102919591)

[Network geometry 25](#_Toc102919592)

[NMA results 25](#_Toc102919593)

[Ranking with SUCRA 26](#_Toc102919594)

[Mean change in central retinal thickness (μm) from baseline 27](#_Toc102919595)

[Network geometry 27](#_Toc102919596)

[NMA results 27](#_Toc102919597)

[Ranking with SUCRA 28](#_Toc102919598)

[Adverse events 28](#_Toc102919599)

[Network geometry 28](#_Toc102919600)

[NMA results 28](#_Toc102919601)

[Ranking with SUCRA 29](#_Toc102919602)

[Appendix 6 Overall heterogeneity, consistency, and forest plots for each comparison 30](#_Toc102919603)

[Mean change in BCVA measured by ETDRS letters from baseline 30](#_Toc102919604)

[Overall heterogeneity and consistency 30](#_Toc102919605)

[Forest plots with heterogeneity test 30](#_Toc102919606)

[The proportion of patients with a gain of at least 15 Early Treatment Diabetic Retinopathy Study (ETDRS) letters (3 ETDRS lines or 0.3 logMAR 32](#_Toc102919607)

[Overall heterogeneity and consistency 32](#_Toc102919608)

[Forest plots with heterogeneity test 33](#_Toc102919609)

[The proportion of patients with a gain of at least 10 Early Treatment Diabetic Retinopathy Study (ETDRS) letters (2 ETDRS lines or 0.2 logMAR 34](#_Toc102919610)

[Overall heterogeneity and consistency 34](#_Toc102919611)

[Forest plots with heterogeneity test 35](#_Toc102919612)

[Mean change in central retinal thickness (μm) from baseline 37](#_Toc102919613)

[Overall heterogeneity and consistency 37](#_Toc102919614)

[Forest plots with heterogeneity test 37](#_Toc102919615)

[Adverse events 39](#_Toc102919616)

[Overall heterogeneity and consistency 39](#_Toc102919617)

[Forest plots with heterogeneity test 39](#_Toc102919618)

[Appendix 7 Node-split plot, trace plot and density plot 40](#_Toc102919619)

[Mean change in BCVA measured by ETDRS letters from baseline 40](#_Toc102919620)

[The proportion of patients with a gain of at least 15 Early Treatment Diabetic Retinopathy Study (ETDRS) letters (3 ETDRS lines or 0.3 logMAR) 42](#_Toc102919621)

[The proportion of patients with a gain of at least 10 Early Treatment Diabetic Retinopathy Study (ETDRS) letters (2 ETDRS lines or 0.2 logMAR) 44](#_Toc102919622)

[Mean change in central retinal thickness (μm) from baseline 46](#_Toc102919623)

[Adverse events 48](#_Toc102919624)

# Appendix 1 Search strategies

## MEDLINE

#1. TS=(diabet* NEAR/2 (macula OR macular) NEAR/2 (edema* OR oedema*) OR DME)

#2. MH:exp=(Macular Edema) OR TS=((macula OR macular) NEAR/1 (edema* OR oedema*))

#3. MH:exp=(Diabetes Mellitus) OR TS=(diabet*)

#4. #1 OR (#2 AND #3) 8006

#5. MH:exp=(Bevacizumab OR Ranibizumab) OR TS=("Anti-VEGF" OR "Anti-vascular-endothelial-growth-factor*" OR VEGF NEAR/2 (antagonist* OR inhibitor*) OR ("vascular-endothelial-growth-factor*" NEAR/4 (antagonist* OR inhibitor*))) OR TS=(conbercept OR faricimab OR RG7716 OR ro6867461 OR "ro-6867461" OR "rg-7716" OR rg7716 OR ranibizumab OR Lucentis OR lucentis OR "rhufab-v2" OR bevacizumab OR Avastin OR "abp-215" OR abp215 OR ainex OR altuzan OR askb1202 OR "bcd-021" OR bevax OR "bevz-92" OR "bi-695502" OR "fkb-238" OR "pf-06439535" OR kyomarc OR mvasi OR "mil 60" OR "nsc-704865" OR nsc704865 OR "sb 8" OR "stc 103" OR Aflibercept OR "ave-0005" OR ave0005 OR eylea OR "vascular-endothelial-growth-factor-trap" OR "vasculotropin-trap" OR "VEGF-Trap" OR zaltrap OR ramucirumab OR "imc 1121 b" OR "imc 1121b")

#6. MH:exp=(Laser Therapy OR Lasers OR Dexamethasone) OR TS=(Laser OR Dexamethasone OR Methylfluorprednisolone OR Hexadecadrol OR cortastat OR cortidexason OR decaderm OR decadron OR Decameth OR Decaspray OR dexamonozon OR dexmethsone OR dexona OR dextenza OR Dexasone OR Dexpak OR Maxidex OR Millicorten OR Oradexon OR Decaject OR Hexadrol OR Ozurdex OR posurdex OR solurex OR visumetazone)

#7. #5 OR #6

#8. #4 AND #7 3358

#9. (MH:exp=(Controlled Clinical Trials as Topic OR Randomized Controlled Trials as Topic OR Double-Blind Method OR single-blind method OR Control Groups OR Random Allocation OR cross-over studies OR Observational Studies as Topic OR Cohort Studies OR Case-Control Studies OR Cross-Sectional Studies) OR TS=(random* OR placebo OR trial OR groups OR crossover OR "cross-over" OR Cohort OR "Follow-Up" OR Longitudinal* OR Prospectiv* OR Retrospectiv* OR Case-Control OR "Cross-Sectional") OR DOCUMENT TYPES:(Controlled Clinical Trial OR Randomized Controlled Trial OR Observational Study)) AND MH:exp=(Humans)

#10. #8 AND #9 1835

## Embase

#1. 'diabetic macular edema'/exp OR (diabet* NEAR/2 (macula OR macular) NEAR/2 (edema* OR oedema*) OR DME):ab,ti,kw

#2. 'macular edema'/exp OR ((macula OR macular) NEAR/1 (edema* OR oedema*)):ab,ti,kw

#3. 'Diabetes Mellitus'/exp OR diabet*:ab,ti,kw

#4. #1 OR (#2 AND #3) 13667

#5. 'bevacizumab'/exp OR 'ranibizumab'/exp OR 'faricimab'/exp OR 'aflibercept'/exp OR 'Ramucirumab'/exp OR 'conbercept'/exp OR Anti-VEGF:ab,ti,kw OR Anti-vascular-endothelial-growth-factor*:ab,ti,kw OR (VEGF NEAR/1 (antagonist* OR inhibitor*)):ab,ti,kw OR (vascular-endothelial-growth-factor* NEAR/4 (antagonist* OR inhibitor*)):ab,ti,kw OR (conbercept OR faricimab OR RG7716 OR ro6867461 OR "ro-6867461" OR "rg-7716" OR rg7716 OR ranibizumab OR Lucentis OR lucentis OR "rhufab-v2" OR bevacizumab OR Avastin OR "abp-215" OR abp215 OR ainex OR altuzan OR askb1202 OR "bcd-021" OR bevax OR "bevz-92" OR "bi-695502" OR "fkb-238" OR "pf-06439535" OR kyomarc OR mvasi OR "mil 60" OR "nsc-704865" OR nsc704865 OR "sb 8" OR "stc 103" OR Aflibercept OR "ave-0005" OR ave0005 OR eylea OR "vascular-endothelial-growth-factor-trap" OR "vasculotropin-trap" OR "VEGF-Trap" OR zaltrap OR ramucirumab OR "imc 1121 b" OR "imc 1121b"):ab,ti,kw

#6. 'Laser'/exp OR 'low level laser therapy'/exp OR 'Dexamethasone'/exp OR (Laser OR Dexamethasone OR Methylfluorprednisolone OR Hexadecadrol OR cortastat OR cortidexason OR decaderm OR decadron OR Decameth OR Decaspray OR dexamonozon OR dexmethsone OR dexona OR dextenza OR Dexasone OR Dexpak OR Maxidex OR Millicorten OR Oradexon OR Decaject OR Hexadrol OR Ozurdex OR posurdex OR solurex OR visumetazone):ab,ti,kw

#7. #5 OR #6

#8. #4 AND #7 6089

#9. (('controlled clinical trial'/exp OR 'Controlled Clinical Trial (Topic)'/exp OR 'double blind procedure'/de OR 'control group'/de OR 'crossover procedure'/de OR 'single blind procedure'/de OR 'triple blind procedure'/de OR 'placebo'/de OR 'randomization'/exp OR 'observational study'/exp OR 'cohort analysis'/exp OR 'cross-sectional study'/exp OR 'case control study'/exp OR 'case report'/exp) OR (random* OR trial OR groups OR placebo* OR crossover OR "cross-over" OR "observational stud*" OR Cohort OR "Follow-Up" OR Longitudinal* OR Prospectiv* OR Retrospectiv* OR "Case-Control" OR "Cross-Sectional" OR "case-series"):ab,ti,kw) AND 'human'/exp

#10. #8 AND #9 4037

## Cochrane library

#1 (diabet* NEAR/2 (macula OR macular) NEAR/2 (edema* OR oedema*) OR DME):ti,ab,kw 2409

#2 MeSH descriptor: [Macular Edema] explode all trees 1121

#3 ((macula OR macular) NEAR/1 (edema* OR oedema*)):ti,ab,kw 3299

#4 #2 or #3 3299

#5 MeSH descriptor: [Diabetes Mellitus] explode all trees 31368

#6 (diabet*):ti,ab,kw 93410

#7 #5 or #6 93641

#8 #4 and #7 2091

#9 #1 or #8 2880

#10 MeSH descriptor: [Bevacizumab] explode all trees 1917

#11 MeSH descriptor: [Ranibizumab] explode all trees 815

#12 ("Anti-VEGF" OR "Anti-vascular-endothelial-growth-factor*" OR VEGF NEAR/2 (antagonist* OR inhibitor*) OR ("vascular-endothelial-growth-factor*" NEAR/4 (antagonist* OR inhibitor*))):ti,ab,kw 97195

#13 (conbercept OR faricimab OR RG7716 OR ro6867461 OR "ro-6867461" OR "rg-7716" OR rg7716 OR ranibizumab OR Lucentis OR lucentis OR "rhufab-v2" OR bevacizumab OR Avastin OR "abp-215" OR abp215 OR ainex OR altuzan OR askb1202 OR "bcd-021" OR bevax OR "bevz-92" OR "bi-695502" OR "fkb-238" OR "pf-06439535" OR kyomarc OR mvasi OR "mil 60" OR "nsc-704865" OR nsc704865 OR "sb 8" OR "stc 103" OR Aflibercept OR "ave-0005" OR ave0005 OR eylea OR "vascular-endothelial-growth-factor-trap" OR "vasculotropin-trap" OR "VEGF-Trap" OR zaltrap OR ramucirumab OR "imc 1121 b" OR "imc 1121b"):ti,ab,kw 8858

#14 MeSH descriptor: [Laser Therapy] explode all trees 4112

#15 MeSH descriptor: [Lasers] explode all trees 2136

#16 MeSH descriptor: [Dexamethasone] explode all trees 4558

#17 (Laser OR Dexamethasone OR Methylfluorprednisolone OR Hexadecadrol OR cortastat OR cortidexason OR decaderm OR decadron OR Decameth OR Decaspray OR dexamonozon OR dexmethsone OR dexona OR dextenza OR Dexasone OR Dexpak OR Maxidex OR Millicorten OR Oradexon OR Decaject OR Hexadrol OR Ozurdex OR posurdex OR solurex OR visumetazone):ti,ab,kw 30399

#18 #10 or #11 or #12 or #13 or #14 or #15 or #16 or #17 132023

#19 #9 and #18 1686

## Web of Science

#1. TS=(diabet* NEAR/2 (macula OR macular) NEAR/2 (edema* OR oedema*) OR DME)

#2. TS=((macula OR macular) NEAR/1 (edema* OR oedema*)) AND TS=(diabet*)

#3. #1 OR #2 16649

#4. TS=("Anti-VEGF" OR "Anti-vascular-endothelial-growth-factor*" OR VEGF NEAR/2 (antagonist* OR inhibitor*) OR ("vascular-endothelial-growth-factor*" NEAR/4 (antagonist* OR inhibitor*))) OR TS=(conbercept OR faricimab OR RG7716 OR ro6867461 OR "ro-6867461" OR "rg-7716" OR rg7716 OR ranibizumab OR Lucentis OR lucentis OR "rhufab-v2" OR bevacizumab OR Avastin OR "abp-215" OR abp215 OR ainex OR altuzan OR askb1202 OR "bcd-021" OR bevax OR "bevz-92" OR "bi-695502" OR "fkb-238" OR "pf-06439535" OR kyomarc OR mvasi OR "mil 60" OR "nsc-704865" OR nsc704865 OR "sb 8" OR "stc 103" OR Aflibercept OR "ave-0005" OR ave0005 OR eylea OR "vascular-endothelial-growth-factor-trap" OR "vasculotropin-trap" OR "VEGF-Trap" OR zaltrap OR ramucirumab OR "imc 1121 b" OR "imc 1121b")

#5. TS=(Laser OR Dexamethasone OR Methylfluorprednisolone OR Hexadecadrol OR cortastat OR cortidexason OR decaderm OR decadron OR Decameth OR Decaspray OR dexamonozon OR dexmethsone OR dexona OR dextenza OR Dexasone OR Dexpak OR Maxidex OR Millicorten OR Oradexon OR Decaject OR Hexadrol OR Ozurdex OR posurdex OR solurex OR visumetazone)

#6. #4 OR #5

#7. #3 AND #6 4591

#8. TS=(Controlled NEAR/1 (Trial OR study) OR "Double-Blind" OR "single-blind" OR random* OR placebo OR trial OR groups OR crossover OR "cross-over" OR ("case control" OR family OR longitudinal OR retrospective OR prospective OR cohort OR "follow up" OR "follow-up" OR observational OR epidemiological OR "cross sectional" OR "cross-sectional") NEAR/1 (study or studies))

#9. #7 AND #8 2271

## CBM

#1. "糖尿病性黄斑水肿"[常用字段:智能] OR "糖尿病黄斑水肿"[常用字段:智能] OR "DME"[常用字段:智能] OR (("黄斑水肿"[不加权:扩展] OR "黄斑水肿"[常用字段:智能]) AND ("糖尿病"[不加权:扩展] OR "糖尿病"[常用字段:智能]))

#2. "Avastin"[常用字段:智能] OR "Bevacizumab"[常用字段:智能] OR "安维汀"[常用字段:智能] OR "贝伐珠单抗"[常用字段:智能] OR "贝伐单抗"[常用字段:智能] OR "阿瓦斯汀"[常用字段:智能] OR "阿瓦斯丁"[常用字段:智能] OR "Ranibizumab"[常用字段:智能] OR "兰尼单抗"[常用字段:智能] OR "雷珠单抗"[常用字段:智能] OR "诺适得"[常用字段:智能] OR "Lucentis"[常用字段:智能] OR "Aflibercept"[常用字段:智能] OR "阿柏西普"[常用字段:智能] OR "阿普西柏"[常用字段:智能] OR "采视明"[常用字段:智能] OR "conbercept"[常用字段:智能] OR "康博西普"[常用字段:智能] OR "康柏西普"[常用字段:智能] OR "雷莫芦单抗"[常用字段:智能] OR "雷莫卢单抗"[常用字段:智能] OR "VEGF单抗"[常用字段:智能] OR "抗VEGF"[常用字段:智能] OR "血管内皮生长因子单抗"[常用字段:智能] OR "抗血管内皮生长因子"[常用字段:智能] OR "激光"[常用字段:智能] OR "镭射"[常用字段:智能] OR "laser"[常用字段:智能] OR "地塞米松"[常用字段:智能] OR "傲迪适"[常用字段:智能]

#3. "临床试验"[常用字段:智能] OR "临床研究"[常用字段:智能] OR "临床评估"[常用字段:智能] OR "临床评价"[常用字段:智能] OR "效果评价"[常用字段:智能] OR "干预研究"[常用字段:智能] OR "疗效"[常用字段:智能] OR "随机"[常用字段:智能] OR "盲法"[常用字段:智能] OR "双盲"[常用字段:智能] OR "单盲"[常用字段:智能] OR "对照"[常用字段:智能] OR "三盲"[常用字段:智能] OR "交叉"[常用字段:智能] OR "RCT"[常用字段:智能] OR "观察性研究"[常用字段:智能] OR "病例对照"[常用字段:智能] OR "队列"[常用字段:智能] OR "横断面"[常用字段:智能] OR "纵向"[常用字段:智能] OR "前瞻"[常用字段:智能] OR "回顾"[常用字段:智能] OR "追踪调查"[常用字段:智能] OR "调查追踪"[常用字段:智能] OR "跟踪调查"[常用字段:智能] OR "随访"[常用字段:智能] OR "病例"[中文标题:智能] OR "临床"[中文标题:智能] OR "疗效"[中文标题:智能]

#4. #1 AND #2 AND #3 976

## Wanfang

主题:(糖尿病性黄斑水肿+糖尿病黄斑水肿+DME*黄斑+糖尿病*黄斑水肿)*(Avastin+Bevacizumab+安维汀+贝伐珠单抗+贝伐单抗+阿瓦斯汀+阿瓦斯丁+Ranibizumab+兰尼单抗+雷珠单抗+诺适得+Lucentis+Aflibercept+阿柏西普+阿普西柏+采视明+conbercept+康博西普+康柏西普+雷莫芦单抗+雷莫卢单抗+VEGF单抗+抗VEGF+血管内皮生长因子单抗+抗血管内皮生长因子+激光+镭射+laser+地塞米松+傲迪适)*(临床试验+临床研究+临床评估+临床评价+效果评价+干预研究+疗效+随机+盲法+双盲+单盲+对照+三盲+交叉+RCT+观察性研究+病例对照+队列+横断面+纵向+前瞻+回顾+追踪调查+调查追踪+跟踪调查+随访) 1528

## CNKI

(SU%=糖尿病性黄斑水肿+糖尿病黄斑水肿+DME*黄斑+糖尿病*黄斑水肿 OR TKA='糖尿病 /NEAR 2 黄斑水肿'+DME*黄斑) AND (SU%=Avastin+Bevacizumab+安维汀+贝伐珠单抗+贝伐单抗+阿瓦斯汀+阿瓦斯丁+Ranibizumab+兰尼单抗+雷珠单抗+诺适得+Lucentis+Aflibercept+阿柏西普+阿普西柏+采视明+conbercept+康博西普+康柏西普+雷莫芦单抗+雷莫卢单抗+VEGF单抗+抗VEGF+血管内皮生长因子单抗+抗血管内皮生长因子+激光+镭射+laser+地塞米松+傲迪适 OR TKA=Avastin+Bevacizumab+安维汀+贝伐珠单抗+贝伐单抗+阿瓦斯汀+阿瓦斯丁+Ranibizumab+兰尼单抗+雷珠单抗+诺适得+Lucentis+Aflibercept+阿柏西普+阿普西柏+采视明+conbercept+康博西普+康柏西普+雷莫芦单抗+雷莫卢单抗+VEGF单抗+抗VEGF+血管内皮生长因子单抗+抗血管内皮生长因子+激光+镭射+laser+地塞米松+傲迪适) AND (TKA=临床试验+临床研究+临床评估+临床评价+效果评价+干预研究+疗效+随机+盲法+双盲+单盲+对照+三盲+交叉+RCT+观察性研究+病例对照+队列+横断面+纵向+前瞻+回顾+追踪调查+调查追踪+跟踪调查+随访 OR TI=病例+临床+疗效 OR SU%=临床试验+临床研究+临床评估+临床评价+效果评价+干预研究+疗效+随机+盲法+双盲+单盲+对照+三盲+交叉+RCT+观察性研究+病例对照+队列+横断面+纵向+前瞻+回顾+追踪调查+调查追踪+跟踪调查+随访) 1480

## VIP

(M=糖尿病性黄斑水肿+糖尿病黄斑水肿+DME*黄斑+糖尿病*黄斑水肿 OR R=糖尿病性黄斑水肿+糖尿病黄斑水肿+DME*黄斑+糖尿病*黄斑水肿) AND (M=Avastin+Bevacizumab+安维汀+贝伐珠单抗+贝伐单抗+阿瓦斯汀+阿瓦斯丁+Ranibizumab+兰尼单抗+雷珠单抗+诺适得+Lucentis+Aflibercept+阿柏西普+阿普西柏+采视明+conbercept+康博西普+康柏西普+雷莫芦单抗+雷莫卢单抗+VEGF单抗+抗VEGF+血管内皮生长因子单抗+抗血管内皮生长因子+激光+镭射+laser+地塞米松+傲迪适 OR R=Avastin+Bevacizumab+安维汀+贝伐珠单抗+贝伐单抗+阿瓦斯汀+阿瓦斯丁+Ranibizumab+兰尼单抗+雷珠单抗+诺适得+Lucentis+Aflibercept+阿柏西普+阿普西柏+采视明+conbercept+康博西普+康柏西普+雷莫芦单抗+雷莫卢单抗+VEGF单抗+抗VEGF+血管内皮生长因子单抗+抗血管内皮生长因子+激光+镭射+laser+地塞米松+傲迪适) AND (R=临床试验+临床研究+临床评估+临床评价+效果评价+干预研究+疗效+随机+盲法+双盲+单盲+对照+三盲+交叉+RCT+观察性研究+病例对照+队列+横断面+纵向+前瞻+回顾+追踪调查+调查追踪+跟踪调查+随访 OR T=病例+临床+疗效 OR M=临床试验+临床研究+临床评估+临床评价+效果评价+干预研究+疗效+随机+盲法+双盲+单盲+对照+三盲+交叉+RCT+观察性研究+病例对照+队列+横断面+纵向+前瞻+回顾+追踪调查+调查追踪+跟踪调查+随访) 1059

# Appendix 2 Characteristics of included RCTs

## Table S1 Characteristics of Study and Population

| **Study ID** | **Trial No. (abbreviation/no.)** | **Country /region** | **Multi-center (yes/no)** | **Sample size enrollment (total patients)** | **Sample size enrollment (total eyes)** | **Description of intervention** | **Gender (male/female)** | **Age, years mean (SD)** | **Number of  (VA <20/40)** | **Randomization stratified by worse baseline vision or not? (yes/no)** | **Diabetes type** | **DME type** | **DME stage or history of DME** | **Baseline VA mean (SD)** | **Baseline CRT, µm mean (SD)** | **Duration of diabetes, year mean (SD)** |
| --- | --- | --- | --- | --- | --- | --- | --- | --- | --- | --- | --- | --- | --- | --- | --- | --- |
| Azad 2012 | NR | India | Yes | 40 | 40 | IVB | 12/8 | 53.6 | NR | NA | Type 2 | Refractory diffuse DME | NR | 20/160 | 456.7 | 13.3 |
|  |  |  |  |  |  | Laser | 10/10 | 56.4 | NR | NA | Type 2 | Refractory diffuse DME | NR | 20/200 | 357.3 | 14.9 |
| Baker 2019* | NCT01909791 | US, Canada | Yes | 466 | 466 | IVA | 131/95 | Median (IQR): 59 (52–65) | 0 | No | Type 1 or 2 | Center-involved DME | NR | 20/20 | 306 (55) | Median (IQR): 15 (10–21) |
|  |  |  |  |  |  | Laser | 158/82 | Median (IQR): 60 (53–66) | 0 | No | Type 1 or 2 | Center-involved DME | NR | 20/20 | 314 (52) | Median (IQR): 15 (10–20) |
| Berger 2015* | NCT01135914 | Canada | Yes | 147 | 147 | IVR | 42/33 | 61.5 (9.9) | NR | No | Type 1 or 2 | Diffuse/focal DME | Mean (SD), y 1.6 (2.3) | 63.1 (10.6) | 448.5 (136.6) | 16.5 (9.0) |
|  |  |  |  |  |  | Laser | 43/29 | 62.8 (9.4) | NR | No | Type 1 or 2 | Diffuse/focal DME | Mean (SD), y 1.7 (2.9) | 61.9 (10.6) | 458.0 (133.1) | 16.6 (10.7) |
| Boyer 2014 | MEAD (NCT00168337 and NCT00168389) | US, Brazil, Canada, Colombia, France, Hungary, India, Italy, Korea, New Zealand, Poland, Singapore, Taiwan, UK, Australia, Czech, Germany, Israel, The Philippines, Portugal, South Africa, Spain | Yes | 1048 | NR | Dex | 213/138 | 62.5(8.3) | NR | NA | Type 1 or 2 | NR | NR | 56.1 (9.9) | 463.0 (157.1) | 16.5 (9.0) |
|  |  |  |  |  |  | Dex | 206/141 | 62.3(9.2) | NR | NA | Type 1 or 2 | NR | NR | 55.5 (9.7) | 466.8 (159.5) | 15.8 (9.4) |
|  |  |  |  |  |  | Sham | 217/133 | 62.5 (9.5) | NR | NA | Type 1 or 2 | NR | NR | 56.9 (8.7) | 460.9 (132.6) | 15.9 (9.1) |
| Boyer 2014 | MEAD (NCT00168337 and NCT00168389) | US, Brazil, Canada, Colombia, France, Hungary, India, Italy, Korea, New Zealand, Poland, Singapore, Taiwan, UK, Australia, Czech, Germany, Israel, The Philippines, Portugal, South Africa, Spain | Yes | 508 | 508 | Dex | 150/97 | 63 (8.3) | NR | NA | Type 2: 220 | NR | NR | 55.2 (9.6) | 478 (153) | 16.4 (8.7) |
|  |  |  |  |  |  | Sham | 168/93 | 63 (9.1) | NR | NA | Type 2: 238 | NR | NR | 56.1 (9.1) | 472 (131) | 16.2 (9.7) |
| Brown 2013*-RIDE | RIDE (NCT00473382) | US, South America | Yes | 382 | 382 | Sham | 66/64 | 63.5 (10.8) | NR | NA | Type 1 or 2 | NR | NR | 57.3 (11.2) | 447.4 (154.4) | 16.6 (10.6) |
|  |  |  |  |  |  | IVR | 73/52 | 62.7 (11.1) | NR | NA | Type 1 or 2 | NR | NR | 57.5 (11.6) | 482.6 (149.3) | 16.0 (9.8) |
|  |  |  |  |  |  | IVR | 80/47 | 61.8 (10.1) | NR | NA | Type 1 or 2 | NR | NR | 56.9 (11.8) | 463.8 (175.5) | 15.3 (10.1) |
| Brown 2013*-RISE | RISE (NCT00473330) | US, South America | Yes | 377 | 377 | Sham | 74/53 | 61.8 (9.8) | NR | NA | Type 1 or 2 | NR | NR | 57.2 (11.1) | 467.3 (152.0) | 14.5 (9.9) |
|  |  |  |  |  |  | IVR | 73/52 | 61.7 (8.9) | NR | NA | Type 1 or 2 | NR | NR | 54.7 (12.6) | 474.5 (174.8) | 15.9 (9.9) |
|  |  |  |  |  |  | IVR | 65/60 | 62.8 (10.0) | NR | NA | Type 1 or 2 | NR | NR | 56.9 (11.6) | 463.8 (144.0) | 16.3 (8.5) |
| Brown 2015*-VISTA | VISTA (NCT01363440) | US | Yes | 466 | 466 | IVA | 87/67 | 62.0 (11.2) | NR | NA | Type 1 or 2 | NR | NR | 58.9 (10.8) | 485 (157) | 16.5 (9.9) |
|  |  |  |  |  |  | IVA | 78/73 | 63.1 (9.4) | NR | NA | Type 1 or 2 | NR | NR | 59.4 (10.9) | 479 (154) | 17.6 (11.5) |
|  |  |  |  |  |  | Laser | 85/69 | 61.7 (8.7) | NR | NA | Type 1 or 2 | NR | NR | 59.7 (10.9) | 483 (153) | 17.2 (9.5) |
| Brown 2015*-VIVID | VIVID (NCT01331681) | Europe, Japan, Australia | Yes | 406 | 406 | IVA | 83/53 | 62.6 (8.6) | NR | NA | Type 1 or 2 | NR | NR | 60.8 (10.7) | 502 (144) | 14.3 (9.2) |
|  |  |  |  |  |  | IVA | 88/47 | 64.2 (7.8) | NR | NA | Type 1 or 2 | NR | NR | 58.8 (11.2) | 518 (147) | 14.1 (8.9) |
|  |  |  |  |  |  | Laser | 78/54 | 63.9 (8.6) | NR | NA | Type 1 or 2 | NR | NR | 60.8 (10.6) | 540 (152) | 14.5 (9.8) |
| Callanan 2016* | NCT01492400 | US, Belgium, Denmark, France, Germany, Israel, Italy, The Netherlands, Portugal, South Africa, Spain, UK | Yes | 363 | 363 | Dex | 112/69 | 63.4 (9.39) | 181 | No | NR | Center-involved DME | Mean (SD), m 36.3 (58.1) | 60.2 (9.74) | 465 (136) | NR |
|  |  |  |  |  |  | IVR | 116/66 | 63.7 (10.05) | 182 | No | NR | Center-involved DME | Mean (SD), m 29.7 (33.3) | 60.4 (9.34) | 471 (140) | NR |
| Chatzirallis 2020 | NR | Greece | No | 112 | 112 | IVR | 28/26 | 64.4 (9.3) | NR | NA | Type 2 | Center-involved DME | NR | 56.3 (6.2) | 424.2 (62.3) | 11.1 (4.9) |
|  |  |  |  |  |  | IVA | 33/25 | 64.8 (8.5) | NR | NA | Type 2 | Center-involved DME | NR | 58.9 (9.3) | 429.5 (64.1) | 12.1 (4.2) |
| Chen 2020* | VIVID-East (NCT01783886) | China, Hong Kong, Korea, Russia | Yes | 381 | 381 | IVA | 59/68 based on full/safety analysis set (n=378) | 59.3 ( 10.3) | 127 | No | Type 1 or 2 | Clinically significant center-involved DME | NR | 55.6 (12.1) | 526.3 (164.4) | 12.9 (7.7) |
|  |  |  |  |  |  | IVA | 67/60 based on full/safety analysis set (n=378) | 57.6 (10.1) | 127 | No | Type 1 or 2 | Clinically significant center-involved DME | NR | 57.1 (12.5) | 520.3 (154.8) | 11.5 (7.9) |
|  |  |  |  |  |  | Laser | 64/60 based on full/safety analysis set (n=378) | 58.8 (10.5) | 124 | No | Type 1 or 2 | Clinically significant center-involved DME | NR | 55.1 (14.2) | 527.7 (170.5) | 12.6 (7.8) |
| Comyn 2014* | LUCIDATE (NCT01223612) | UK | No | 37 | 37 | IVR | 15/7 Completers | Median (IQR): 64.9 (58.4–71.0) | NR | NA | Type 1 or 2 | Center-involved DME | Median (IQR): 21 (14–27) | 70.4 (4.9) | 455.4 (78.7) | median (IQR): 18.5 (10–26) |
|  |  |  |  |  |  | Laser | 6/5 Completers | Median (IQR): 67.4 (62.8–74.6) | NR | NA | Type 1 or 2 | Center-involved DME | Median (IQR): 32 (15–60) | 63.8 (5.7) | 488.2 (96.3) | median (IQR): 18 (14–25) |
| Do 2013* | READ-2 (NCT00407381) | US | Yes | 84 | NR | IVR | NR | NR | 28 | No | Type 1 or 2 | NR | NR | NR | 414 | NR |
|  |  |  |  |  |  | Laser | NR | NR | 22 | No | Type 1 or 2 | NR | NR | NR | 458 | NR |
| Du 2017 | NR | China | NR | 80 | 80 | IVR | 21/19 | 47.45(9.24) | NR | NA | Type 2 | Severe NPDR macular edema | NR | 0.67 (0.28) | 219.57 (51.24) (Choroidal thickness under the fovea) | NR |
|  |  |  |  |  |  | Laser | 23/17 | 48.15(10.85) | NR | NA | Type 2 | Severe NPDR macular edema | NR | 0.64 (0.30) | 217.56 (50.36) (Choroidal thickness under the fovea) | NR |
| Fan 2019 | NR | China | No | 46 | 46 | IVC | 12/11 | 53.7 (10.6) | NR | NA | NR | NR | NR | Decimal 0.21 (0.08) | 451.9 (85.4) | NR |
|  |  |  |  |  |  | Laser | 13/10 | 54.1 (10.3) | NR | NA | NR | NR | NR | Decimal 0.22 (0.07) | 451.3 (85.2) | NR |
| Fouda 2017* | NR | Egypt | Yes | 42 | 70 | IVA | NR | 55.05 (4.7) | NR | NA | Type 1 or 2 | DME | NR | 0.17 (0.05) | 465.29 (33.7) | NR |
|  |  |  |  |  |  | IVR | NR | 56.64 (5.8) | NR | NA | Type 1 or 2 | DME | NR | 0.18 (0.04) | 471.5 (34.4) | NR |
| Gillies 2014* | BEVORDEX (NCT01298076) | Australia | Yes | 61 | 88 | IVB | 26/16 | 62.2 (10.5) | NR | NA | NR | NR | NR | 56.3 (11.9) | 503 (140.9) | 16.7 (10.7) |
|  |  |  |  |  |  | Dex | 30/16 | 61.4 (9.0) | NR | NA | NR | NR | NR | 55.5 (12.5) | 474.3 (95.9) | 16.7 (10.3) |
| Gu 2015 | NR | China | No | 82 | 82 | Laser | 49/33 (total) | 60.51 (10.27) | NR | NA | Type 1 | NR | NR | 0.09 (0.041) | 519.09 (138.77) | NR |
|  |  |  |  |  |  | IVR | 49/33 (total) | 50.9 (6.94) | NR | NA | Type 1 | NR | NR | 0.1 (0.043) | 575.09 (137.12) | NR |
| Jiang 2015 | NR | China | No | 48 | 54 | Laser | NR | NR | NR | NA | NR | NR | NR | 0.33 (0.25) | NR | NR |
|  |  |  |  |  |  | IVR | NR | NR | NR | NA | NR | NR | NR | 0.32 (0.38) | NR | NR |
| Li 2015* | NR | China | No | 68 | 68 | Laser | 19/15 | 58.13 (10.86) | NR | NA | NR | NR | NR | 0.26 (0.09) | 404.8 (54.12) | NR |
|  |  |  |  |  |  | IVR | 22/12 | 56.33 (12.84) | NR | NA | NR | NR | NR | 0.27 (0.1) | 398.1 (41.07) | NR |
| Li 2019* | REFINE (NCT02259088) | China | No | 384 | 384 | IVR | 139/168 | 58.6 (8.70) | NR | NA | Type 1 or 2 | Focal or diffuse DME | Mean (SD), y 1.31 (2.01) | 59.6 (10.53) | 473.4 (166.13) | NR |
|  |  |  |  |  |  | Laser | 39/38 | 59.0 (9.19) | NR | NA | Type 1 or 2 | Focal or diffuse DME | Mean (SD), y 1.10 (1.47) | 58.2 (9.43) | 475.0 (161.52) | NR |
| Liu 2021* | NCT02194634 | China | Yes | 251 | 251 | IVC | 66/59 | 58.9 (8.5) | 125 | No | Type 1 or 2 | DME involving the central fovea | NR | 56.6 (11.5) | 480.0 (180.0) | 12.3 (7.0) |
|  |  |  |  |  |  | Laser | 60/63 | 58.7 (8.8) | 123 | No | Type 1 or 2 | DME involving the central fovea | NR | 57.6 (11.5) | 470.0 (160.0) | 11.0 (6.3) |
| Massin 2010* | RESOLVE (NCT00284050) | NR | Yes | 151 | 151 | IVR | 29/22 | Mean (range): 63.2 (37–85) | NR | No | Type 1 or 2 | DME | Mean (range), y 1.2 (0.0–7.2) | 59.2 (10.2) | 459.5 (109.1) | Mean (range): 14.4 (1.4–36.0) |
|  |  |  |  |  |  | IVR | 27/24 | Mean (range): 62.8 (32–84) | NR | No | Type 1 or 2 | DME | Mean (range), y 1.1 (0.0–7.2) | 61.2 (9.5) | 451.3 (120.1) | Mean (range): 13.9 (0.7–46.0) |
|  |  |  |  |  |  | Sham | 25/24 | Mean (range): 65.0 (41–82) | NR | No | Type 1 or 2 | Center-involved DME | Mean (range), y 1.4 (0.0–19.8) | 61.1 (9.0) | 448.9 (102.8) | Mean (range): 15.1 (2.1–45.8) |
| Mitchell 2011* | RESTORE (NCT00687804) and extension (NCT00906464) | Turkey, Canada, Australia | Yes | 227 | 227 | IVR | 73/43 | 62.9 (9.29) | NR | No | Type 1/2: 13/103 | Focal: 64; Diffuse: 45; Missing: 7 | NR | 64.8 (10.11) | 46.6 (118.01) | 15.23 (9.91) |
|  |  |  |  |  |  | Laser | 58/53 | 63.5 (8.81) | NR | No | Type 1/2: 13/97 | Focal: 53; Diffuse: 52; Missing: 6 | NR | 62.4 (11.11) | 412.4 (123.95) | 12.93 (9.02) |
| Morioka 2018 | UMIN000025769 | Japan | No | 80 | 80 | IVR | 13/7 | 57.5 (2.51) | NR | No | Type 2 | DME | NR | NR | NR | NR |
|  |  |  |  |  |  | IVA | 12/8 | 54.2 (3.31) | NR | No | Type 2 | DME | NR | NR | NR | NR |
| Mukkamala 2017* | DRCR.net Protocol T (NCT01627249) | US | Yes | 660 | 660 | IVR | 124/94 | 60 (11) | 109 | No | Type 1/2/uncertain: 16/196/6 | Center-involved DME | NR | Median (IQR): 68 (73, 58), 20/50 (20/40, 20/80) | Median (IQR): 390 (310, 493) | Median (IQR): 16 (11, 23) |
|  |  |  |  |  |  | IVA | 114/110 | 60 (10) | 112 | No | Type 1/2/uncertain: 22/196/6 | Center-involved DME | NR | Median (IQR): 69 (74, 59), 20/40 (20/32, 20/63) | Median (IQR): 387 (310, 483) | Median (IQR): 15 (8, 21) |
|  |  |  |  |  |  | IVB | 115/103 | 62 (10) | 109 | No | Type 1/2/uncertain: 12/205/1 | Center-involved DME | NR | Median (IQR): 69 (72, 60), 20/40 (20/40, 20/63) | Median (IQR): 376 (305, 477) | Median (IQR): 17 (11, 24) |
| Nepomuceno 2013* | NCT01487629 | Brazil | Yes | 45 | 60 | IVR | 14/14 | 63.7 (9.0) | NR | No | NR | NR | NR | 0.63 (0.06) | 421.9 (23.1) | 15.9 (8.0) |
|  |  |  |  |  |  | IVB | 13/19 | 63.8 (8.8) | NR | No | NR | NR | NR | 0.60 (0.05) | 451.7 (22.3) | 16.2 (8.0) |
| Ozsaygili 2019* | NR | NR | No | 62 | 98 | Dex | 15/14 | 64.8 (7.9) | NR | No | NR | NR | NR | 46.3 (4.4) | 615.2 (150.4) | 10.4 (2.8) |
|  |  |  |  |  |  | IVA | 20/13 | 6.4 (2.0) | NR | No | NR | NR | NR | 47.5 (3.1) | 576.5 (75.3) | 10.2 (2.5) |
| Podkowinski 2020 | NR | Austria | NR | 18 | 18 | IVR | NR | 66.89 (8.80) | NR | No | NR | NR | NR | 74.78 (14.85) | 440.89 (144.47) | NR |
|  |  |  |  |  |  | Dex | NR | 64.56 (9.0) | NR | No | NR | NR | NR | 67.22 (10.52) | 471.33 (122.60) | NR |
| Qin 2020 | NR | China | No | 116 | 116 | IVR | 31/27 | 61.5 (8.56) | NR | NA | NR | NR | NR | 1.62 (0.12) | 396.18 (85.63) | 12.6 (3.15) |
|  |  |  |  |  |  | IVC | 33/25 | 61.2 (8.25) | NR | NA | NR | NR | NR | 1.62 (0.13) | 395.24 (90.12) | 12.5 (2.56) |
|  |  |  |  |  |  | IVR | 73/52 | 62.7 (11.1) | NR | NA | Type 1 or 2 | NR | NR | 57.5 (11.6) | 482.6 (149.3) | 16.0 (9.8) |
|  |  |  |  |  |  | IVR | 80/47 | 61.8 (10.1) | NR | NA | Type 1 or 2 | NR | NR | 56.9 (11.8) | 463.8 (175.5) | 15.3 (10.1) |
|  |  |  |  |  |  | IVR | 73/52 | 61.7 (8.9) | NR | NA | Type 1 or 2 | NR | NR | 54.7 (12.6) | 474.5 (174.8) | 15.9 (9.9) |
|  |  |  |  |  |  | IVR | 65/60 | 62.8 (10.0) | NR | NA | Type 1 or 2 | NR | NR | 56.9 (11.6) | 463.8 (144.0) | 16.3 (8.5) |
| Sarda 2020 | NR | France | No | 24 | 24 | IVA | 16/8 (total sex ratio 2.14) | 62.1 | NR | No | Type 2: 11 | NR | NR | 64.5 | 204.5 (Choroidal thickness) | NR |
|  |  |  |  |  |  | IVR | 16/8 (total sex ratio 2.14) | 60 | NR | No | Type 2: 10 | NR | NR | 67.1 | 270.5 (Choroidal thickness) | NR |
| Scott 2007 | NCT00336323 | NR | Yes | 87 | 87 | Laser | 10/9 | Median (quartiles): 64 (57, 72) | NR | NA | Type 1/2: 1/18 | Typical/predominantly focal: 6; neither predominantly focal or diffuse: 4; typical/predominantly diffuse: 9 | NR | Median (quartiles): 64 (50, 70) | CST (µm); median (quartiles): 441 (354, 512) | Median (quartiles): 17 (13, 22) |
|  |  |  |  |  |  | IVB | 16/6 | Median (quartiles): 63 (54, 73) | NR | NA | Type 1/2: 1/21 | Typical/predominantly focal: 5; neither predominantly focal or diffuse: 5; typical/predominantly diffuse: 12 | NR | Median (quartiles): 65 (60, 70) | CST (µm); median (quartiles): 397 (320, 538) | Median (quartiles): 15 (8, 22) |
|  |  |  |  |  |  | IVB | 15/9 | Median (quartiles): 68 (59, 75) | NR | NA | Type 1/2: 3/21 | Typical/predominantly focal: 2; neither predominantly focal or diffuse: 5; typical/predominantly diffuse: 17 | NR | Median (quartiles): 63 (57, 71) | CST (µm); median (quartiles): 446 (342, 543) | Median (quartiles): 18 (12, 22) |
|  |  |  |  |  |  | IVB | 13/9 | Median (quartiles): 60 (54, 75) | NR | NA | Type 1/2: 2/20 | Typical/predominantly focal: 3; neither predominantly focal or diffuse: 6; typical/predominantly diffuse: 13 | NR | Median (quartiles): 64 (52, 68) | CST (µm); median (quartiles): 406 (353, 520) | Median (quartiles): 17 (11, 25) |
| Soheilian 2009* | NCT00370669 | Iran | NR | 100 | 129 | IVB | 27/23 | 60.5 (5.9) | NR | NA | NR | NR | NR | logMAR: 0.71 (0.28) | CMT: 341 (149) | 10.5 (3.2) |
|  |  |  |  |  |  | Laser | 22/28 | 61.0 (5.3) | NR | NA | NR | NR | NR | logMAR: 0.55 (0.26) | CMT: 300 (118) | 10.5 (2.9) |
| Solaiman 2010 | NR | Saudi Arabia | Yes | 31 | 40 | Laser | 11/8 | 57 (49–69) | NR | NA | NR | NR | NR | logMAR: 0.84 (0.15) | 477.14 (39.11) | 17.62 (2.73) |
|  |  |  |  |  |  | IVB | 11/10 | 56 (43–72) | NR | NA | NR | NR | NR | logMAR : 0.84 (0.74) | 482.11 (40.15) | 19.34 (3.06) |
| Vader 2020 | NCT01635790/NTR3247 | The Netherlands | Yes | 170 | NR | IVB | 44/40 | 63.9 (11.6) | NR | No | Type 1/2: 10/74 | NR | NR | 69.0 (1.0) | 450.2 (91.9) | 15.4 (8.8) |
|  |  |  |  |  |  | IVR | 57/25 | 64.9 (11.6) | NR | No | Type 1/2: 12/71 | NR | NR | 68.5 (10.2) | 465.9 (104.6) | 17.5 (13.4) |
| Wiley 2016 | NCT01610557 | US, UK | Yes | 56 | 62 | IVB | 38/24 (total) | NR | NR | No | Type 1/2: 10/83 | NR | NR | NR | NR | NR |
|  |  |  |  |  |  | IVR | 38/24 (total) | NR | NR | No | Type 1/2: 11/82 | NR | NR | NR | NR | NR |
| Wu 2020 | NR | China | No | 42 | 49 | IVR | 12/9 | 61.32 (2013) | NR | NA | Type 2 | NR | Severe DME | 0.138 (0.023) | 485.34 (16.82) | NR |
|  |  |  |  |  |  | Laser | 11/10 | 60.37 (2.53) | NR | NA | Type 2 | NR | Severe DME | 0.135 (0.021) | 486.52 (15.97) | NR |
| Xiang 2018 | NR | China | No | 60 | NR | IVC | 14/16 | 58.97 (6.48) | NR | NA | NR | Diffuse | NR | 0.08 (0.05) | 412.1 (81.5) | NR |
|  |  |  |  |  |  | IVR | 13/17 | 61.03 (7.12) | NR | NA | NR | Diffuse | NR | 0.07 (0.04) | 415.7 (90.4) | NR |
| Yan 2017 | NR | China | No | 96 | 96 | IVC | 51/45 (total) | 58.5 (42–73) (total) | NR | NA | NR | NR | NR | Decimal 0.06 (0.01) | 665.45 (154.4) | NR |
|  |  |  |  |  |  | Laser | 51/45 (total) | 58.5 (42–73) (total) | NR | NA | NR | NR | NR | Decimal 0.06 (0.02) | 583.36 (170.41) | NR |
| Yang 2018* | NR | China | No | 100 | 103 | Laser | 27/23 | 58.54 (9.87) | NR | No | NR | NR | NR | 0.84 (0.17) | 474.31 (93.83) | 7.42 (1.27) |
|  |  |  |  |  |  | IVR | 29/21 | 58.66 (9.90) | NR | No | NR | NR | NR | 0.78 (0.16) | 468.99 (92.25) | 7.37 (1.24) |
| Yang 2020 | NR | China | No | 82 | 82 | IVR | 21/20 | 61.42 (5.28) | NR | NA | NR | NR | Mild: 11; moderate: 18; severe: 12 | 1.62 (0.26) | 475.14 (72.08) | NR |
|  |  |  |  |  |  | IVC | 24/17 | 61.37 (5.3) | NR | NA | NR | NR | Mild: 10; moderate: 17; severe: 14 | 1.57 (0.3) | 475.26 (71.84) | NR |
| Yu 2018 | NR | China | No | 42 | 40 | IVR | 34/26 (total) | 55.73 (8.39) (total) | NR | NA | Type 2 | NR | NR | 0.41 (0.17) | 487.19 (117.78) | 9.6 (total) |
|  |  |  |  |  |  | Laser | 34/26 (total) | 55.73 (8.39) (total) | NR | NA | Type 2 | NR | NR | 0.43 (0.16) | 469.95 (121.49) | 9.6 (total) |
| Zheng 2017* | NR | China | No | 106 | 106 | IVR | 30/27 | 55.12 (11.34) | NR | NA | Type 2 | Focal DME, CSME | NR | 0.25 (0.1) | 387.12 (34. 16) | NR |
|  |  |  |  |  |  | Laser | 25/24 | 56.20 (10.24) | NR | NA | Type 2 | Focal DME, CSME | NR | 0.26 (0.09) | 410.75 (38. 67) | NR |

* represents study included in the network meta-analysis. CRT, central retinal thickness; CSME, clinically significant macular edema; CST, central subfield thickness; Dex, dexamethasone implant; DME, diabetic macular edema; IQR, interquartile range; IVA, aflibercept; IVB, bevacizumab; IVC, conbercept; IVR, ranibizumab; NA, not available; NPDR, nonproliferative diabetic retinopathy; NR, not reported; RCT, randomized controlled trial; SD, standard deviation; Sham, placebo; VA, visual acuity

## Table S2 Inclusion and Exclusion of included RCTs

| **Study ID** | **Inclusion Criteria** | **Exclusion Criteria** |
| --- | --- | --- |
| Azad 2012 | 1) A written, informed consent was obtained from all the patients prior to enrolment in the study; 2) Diffuse diabetic macular edema (DME) on fundus fluorescein angiography (FFA) refractory to at least two prior sessions of macular laser photocoagulation; 3) Central macular thickness (CMT) greater than 250 microns on time domain-optical coherence tomography (TD-OCT) without any evidence of vitreo-retinal traction and having good metabolic control (glycated hemoglobin [HbA_1c_] <7.0%) | 1) Having received prior intraocular, peribulbar or systemic steroids or prior anti-VEGF (vascular endothelial growth factor) therapy; 2) Having uncontrolled diabetes mellitus, diabetic nephropathy, uncontrolled hypertension; 3) With a history of myocardial infarction (MI), stroke or other thromboembolic episode; 4) Patients with one eye; 5) Patients who were not available for a follow-up duration of at least 6 months |
| Baker 2019 | 1) ≥18 years old with type 1 or 2 diabetes; 2) Central-involved DME involving the center of the macula on ophthalmoscopic examination and confirmed on OCT as central subfield thickening at two consecutive visits 1 to 28 days apart (screening and randomization); 3) Best-corrected visual acuity (BCVA) letter score was at least 79 (Snellen equivalent of 20/25 or better) at screening and randomization; 4) Only one eye per participant was included. If both eyes were eligible, the investigator and participant selected the eye to be enrolled | 1) Laser photocoagulation or intravitreous treatment for DME in the past 12 months; 2) More than 1 laser photocoagulation or 4 intraocular injections at any time |
| Berger 2015 | 1) ≥18 years of age; 2) A diagnosis of type 1 or 2 diabetes mellitus as per the American Diabetes Association (ADA) or World Health Organization (WHO) guidelines, considered to be stable and with HbA_1c_ ≤10%; 3) Visual impairment caused by focal or diffuse DME with a BCVA score between 78 and 39 letters, based on Early Treatment Diabetic Retinopathy Study (ETDRS)-like testing charts at a testing distance of 4 m | 1) Presence of a concomitant condition in the study eye that could, in the opinion of the investigator, prevent the improvement of visual acuity (VA) while on study treatment; 2) Active intraocular inflammation or active infection in either eye; 3) History of uveitis in either eye;  4) Uncontrolled glaucoma or neovascularization of the iris in either eye; 5) Evidence of vitreo-macular traction or active proliferative diabetic retinopathy (DR) in the study eye; 6) Pan-retinal laser photocoagulation (within 6 months) or focal/grid laser photocoagulation (within 3 months); 7) History of vitrectomy or intraocular surgery (within 3 months) in the study eye; 8) History of a stroke or renal failure; 9) Blood pressure (BP) readings of systolic greater than 160 mmHg or diastolic greater than 100 mmHg, untreated hypertension, or change in antihypertensive treatment within 3 months before baseline |
| Boyer 2014 | 1) Patients ≥18 years of age diagnosed with type 1 or 2 diabetes mellitus;  2) Had fovea-involved macular edema that was associated with DR and had been previously treated with medical or laser therapy;  3) Treatment-naïve patients who had refused laser treatment or who, in the opinion of the investigator, would not benefit from laser treatment | 1) Intravitreal anti-VEGF treatment within 3 months of study entry, history of intravitreal steroid other than triamcinolone acetonide, intravitreal triamcinolone acetonide or periocular depot of steroid within 6 months of study entry;  2) Intraocular laser or incisional surgery within 90 days of study entry;  3) Glaucoma, ocular hypertension (untreated intraocular pressure [IOP] >23 mmHg, IOP >21 mmHg treated with 1 antiglaucoma medication, or use of ≥2 antiglaucoma medications);  4) Aphakia or an anterior chamber intraocular lens, history of pars plana vitrectomy, and active iris or retinal neovascularization |
| Brown 2013-RIDE | 1) Patients aged ≥18 years; 2) With diabetes mellitus (type 1 or 2); 3) Vision loss due to DME (BCVA 20/40–20/320 Snellen equivalent) and macular edema | 1) Prior vitreoretinal surgery, or a recent history (within 3 months of screening) of pan-retinal or macular laser in the study eye, intraocular corticosteroids, or anti-angiogenic drugs  2) Patients with uncontrolled hypertension, un- controlled diabetes (HbA_1c_ >12%), or recent (within 3 months) cerebrovascular accident (CVA), or MI |
| Brown 2013-RISE | 1) Patients aged ≥18 years; 2) With diabetes mellitus (type 1 or 2); 3) Vision loss due to DME (BCVA 20/40–20/320 Snellen equivalent) and macular edema | 1) Prior vitreoretinal surgery, or a recent history (within 3 months of screening) of pan-retinal or macular laser in the study eye, intraocular corticosteroids, or anti-angiogenic drugs  2) Patients with uncontrolled hypertension, un- controlled diabetes (HbA_1c_ >12%), or recent (within 3 months) CVA, or MI |
| Brown 2015-VISTA | 1) Adult patients with type 1 or type 2 diabetes mellitus who presented with DME with central involvement;  2) BCVA was between 73 and 24 letters (20/40–20/320 Snellen equivalent) | NR |
| Brown 2015-VIVID | 1) Adult patients with type 1 or type 2 diabetes mellitus who presented with DME with central involvement;  2) BCVA was between 73 and 24 letters (20/40–20/320 Snellen equivalent) | NR |
| Callanan 2016 | 1) ≥18 years old; 2) BCVA >34 and <70 ETDRS letters (approximately 20/200–20/40 Snellen equivalent); 3) The VA decrease was due to DME involving the center of the macula with mean central retinal thickness (CRT) in the 1-mm central macular subfield by spectral-domain OCT (SD-OCT) ≥300 um with Spectralis (Heidelberg) or ≥275 um with Cirrus (Zeiss), as determined by the investigator at the screening visit; 4) If both eyes were eligible for the study, the eye with better BCVA was selected as the study eye | 1) HbA_1c_ >12% at baseline; 2) IOP >22 mmHg at screening or on Day 1; 3) A diagnosis of glaucoma; 4) A history of laser treatment within 3 months prior to screening; 5) Use of anti-VEGF treatment within 3 months prior to screening; 6) Use of intravitreal triamcinolone acetonide within 6 months prior to screening; 7) A history of vitrectomy |
| Chatzirallis 2020 | 1) Type 2 diabetes mellitus; 2) Center-involved DME with CRT ≥320 um; 3) In patients with bilateral DME, one eye was randomly chosen per patient | 1) Age-related macular degeneration (AMD), retinal vein occlusion, other retinal diseases except for DME; 2) Vitreo-macular traction, intraocular inflammation, cornea disorders, media opacities, uncontrolled glaucoma, high myopia >6D, previous trauma; 3) Intraocular surgery within the last 6 months; 4) Lost to follow-up |
| Chen 2020 | 1) ≥18 years old; 2) Type 1 or 2 diabetes mellitus; 3) Clinically significant DME involving the center of the macula, defined as the area of the center subfield of OCT; 4) CRT ≥300 um assessed by OCT; 5) BCVA ETDRS letter score between 73 and 24 (20/40–20/320 Snellen equivalent); 6) Only 1 eye per patient was enrolled in the study | 1) Ocular condition with a poorer prognosis in the fellow eye than in the study eye; 2) Any surgical interventions or laser photocoagulation in the study eye within 120 and 90 days of Day 1, respectively; 3) Any treatments with corticosteroids or anti-angiogenic drugs in either eye within 90 days of Day 1; 4) Active proliferative DR in the study eye; 5) A history of idiopathic or autoimmune uveitis in the study eye |
| Comyn 2014 | 1) Adult patients with type 1 or 2 diabetes; 2) BCVA of 55–79 ETDRS letters (20/30–20/80 Snellen equivalent) resulting from center-involved DME; 3) Spectralis OCT (Heidelberg Engineering GmbH, Heidelberg, Germany) central subfield thickness (CST) of 300 mm or more in the study eye; 4) If both eyes were eligible, the eye with worse VA became the study eye | 1) Uncontrolled glaucoma; 2) Aphakia; 3) Cataract precluding fundus photography; 4) External ocular infections; 5) Previous anti-VEGF or laser treatment in the preceding 3 months in both eyes; 6) Angiographic evidence of macular ischemia defined as foveal avascular zone greatest linear dimension of more than 1000 mm or severe perifoveal capillary loss; 7) Other causes of macular edema; for example, after cataract surgery; 8) Other causes of visual loss in the study eye; 9) Other diseases that may affect the course of macular edema in the study eye; 10) Proliferative DR, either active or treated within the previous 3 months |
| Do 2013 | 1) ≥18 years or older with type 1 or type 2 diabetes mellitus; 2) DME was eligible if they had reduction in VA between 20/40 and 20/320; 3) Foveal thickness measured by TD-OCT at 250 μm or greater; 4) HbA_1c_ level of 6% or greater within 12 months before randomization (to convert HbA_1c_ to a proportion of 1, multiply by 0.01); 5) No potential contributing causes to reduced VA other than DME; 6) Reasonable expectation that scatter laser photocoagulation would not be required for the next 6 months; 7) If both eyes were eligible, the eye with the greater CST was entered | 1) Received focal/grid laser treatment within 3 months; 2) Received intraocular injection of a corticosteroid within 3 months, or intraocular injection of a VEGF antagonist within 2 months |
| Du 2017 | 1) The patient meets the pathologic diagnostic criteria for type 2 diabetes; 2) The patient meets the international clinical grading standard of DME for moderate to severe diagnosis; 3) Age >18 years old, no gender limit, voluntary participation in this study, with the consent of the patient and his family | 1) Exclude patients with type 1 diabetes; 2) Exclude patients with severe glaucoma and cataracts and other eye diseases; 3) Exclude patients with acute metabolic disorders in the past 6 months; 4) Exclude patients with major organ diseases, such as cardiovascular, liver and kidney damage |
| Fan 2019 | 1) DME diagnosed by OCT, FFA, and fundus examination | 1) Macular edema caused by other reasons; 2) History of ophthalmic surgery, such as intra-ball injection, fundus laser, turbid refractive media, macular capillary non-perfusion, and severe optic neuropathy |
| Fouda 2017 | 1) Type 1 or 2 diabetes; 2) DME diagnosed clinically and with OCT; 3) BCVA ranged from 0.1 to 0.25 (moderate visual loss); 4) Edema affecting the central 1 mm of the macula (detected by OCT) | 1) Eyes with vascular retinal disorders other than DR (e.g. choroidal neovascularization); 2) Eyes that received previous intravitreal injection of any agents; 3) Eyes with previous intraocular surgery or with previous laser treatment; 4) Eyes with proliferative DR |
| Gillies 2014 | 1) Eyes with DME affecting the central fovea at least 3 months after at least 1 session of laser treatment, or for whom the investigator believed that laser treatment would be unhelpful, with BCVA of 17–72 logarithm of the minimum angle of resolution letters (20/400–20/40 Snellen equivalent) were eligible and identified consecutively as they were seen in the clinics | 1) Uncontrolled glaucoma or glaucoma controlled with more than 1 medication, loss of vision due to other causes, intercurrent severe systemic disease, or any condition affecting follow-up or documentation |
| Gu 2015 | 1) Uyghur patients diagnosed with type 2 diabetes and diagnosed with DR by fundus fluoroscopy; 2) Vision ≤0.5; 3) Onset time ≤1–6 months; 4) OCT and FFA showed macular edema, CMT ≥300 μm; 5) No other treatments; 6) Good compliance with follow-up after treatment and can cooperate and complete the follow-up | 1) History of uveitis or active uveitis; 2) History of AMD; 3) With pathologic myopia; 4) History of glaucoma or a family history of glaucoma; 5) Cataracts or other eye diseases that may affect vision due to turbid refractive media; 6) Obvious vitreous hemorrhage, organization, and traction retinal detachment; 7) Received retinal photocoagulation or intravitreal injection; 8) Other systemic diseases, such as liver and kidney failure, heart failure, and blood diseases; 9) Cannot cooperate to complete treatment and follow-up due to other reasons |
| Jiang 2015 | 1) Meets the diagnostic criteria for diabetes; 2) Ophthalmoscope, FFA and OCT examinations are in line with the characteristics of DME; 3) Patients with diabetes have thickening or hard exudation of the retina within one optic disc diameter from the center of the macula; 4) Receiving ranibizumab treatment and signing informed consent | 1) Other eye diseases such as glaucoma, chronic dacryocystitis, AMD, and cataract; 2) Severe proliferative retinopathy or vitreous hemorrhage; premacular membrane or macular ischemia; 3) Eye trauma, and patients with a history of intraocular surgery, accompanied by other vitreous and retinopathy patients; 4) Primary hypertension and poor BP control, severe heart, liver, and renal insufficiency, and patients who have also used traditional Chinese medicine; 5) No treatment/treatment: those who were lost to follow-up before the end |
| Li 2015 | 1) Diabetes was diagnosed by the Department of Endocrinology; 2) VA ≤0.5; 3) FFA and OCT confirmed that the VA caused by DME decreased and the macula CMT ≥250 μm | 1) Undergone retinal photocoagulation in the past 4 months or plans to undergo pan-retinal photocoagulation in the next 6 months; 2) Have a history of intraocular surgery within the past 4 months; 3) IOP ≥25 mmHg (1 kPa = 7.5 mmHg); 4) Other eye or systemic diseases that affect macular edema; 5) Systolic BP >180 mmHg or diastolic BP >110 mmHg; 6) History of MI or CVA included in the study |
| Li 2019 | 1) Chinese male and female ≥18 years old; 2) Type 1 or 2 diabetes mellitus; 3) HbA_1c_ ≤10.0% at screening; 4) Visual impairment due to focal or diffuse DME with a BCVA score between 78 and 39 letters (inclusively, approximately 20/32–20/160 Snellen equivalent), as measured by ETDRS-like charts at 4 m; 5) If both eyes were eligible, the eye with the worse VA at screening or baseline visits was selected as the study eye, unless the eye with the better VA was deemed to be more appropriate for study by the investigator based on medical reasons | 1) Patients with any type of systemic disease, including those who had received treatment for it or any medical condition (controlled or uncontrolled) that could be expected to progress, recur, or change to an extent that it might influence the assessment of the clinical status of the patient to a significant degree or put the patient at special risk; 2) Uncontrolled systolic BP of >160 mmHg or diastolic BP of >100 mmHg; 3) Laser (pan-retinal, focal, grid) photocoagulation within 3 months prior to baseline visit (study eye) |
| Liu 2021 | 1) >18 years of age; 2) type I or II diabetes mellitus; 3) haemoglobin A1c (HbA1c) of <10% 4) the study eye had to meet the following criteria: (i) DME involving the central fovea, (ii) ETDRS best-corrected visual acuity (BCVA) between 73 and 24 letters (Snellen equivalent of 20/40–20/320), (iii) central retinal thickness (CRT) of >300 μm according to optical coherence tomography (OCT) imaging, (iv) clear ocular media and adequate pupil dilation for examination and imaging and (5) the ETDRS BCVA of the subject’s non-target eye of ≥24 letters (equivalent to 20/320 of the Snellen vision). | 1) active eye infection in either eye; 2) any ophthalmic conditions leading to macular oedema or alterations in vision other than diabetic retinopathy; 3) panretinal photocoagulation within 6 months prior to screening or local/grid retinal photocoagulation within 3 months prior to screening;  4) treatment with anti-VEGF drugs (eg, aflibercept, pegaptanib sodium, ranibizumab, bevacizumab, etc) within 6 months prior to screening; 5) any type of intraocular surgery (eg, cataract surgery, yttrium aluminium garnet (YAG) posterior capsulotomy, etc) within 3 months prior to screening;  6) uncontrolled hypertension; 7) stroke, transient ischaemic attack, myocardial infarction or acute congestive heart failure within 6 months prior to screening. |
| Massin 2010 | 1) ≥18 years old with type 1 or 2 diabetes; 2) VA between 20/40 and 20/160, CRT ≥300 um, HbA_1c_ ≤12%, decreased vision attributed to foveal thickening from DME, that was not explained by any other cause; 3) Clinically significant DME in at least one eye confirmed by a central reading center using stereoscopic fundus photographs, fluorescein angiography, and OCT; 4) In the judgment of the investigator, laser photocoagulation could be safely withheld in the study eye for at least 3 months after random assignment | 1) Unstable medical status, including glycemic control and BP; 2) Pan-retinal laser photocoagulation performed within 6 months before study entry; 3) Grid/central laser photocoagulation was excluded except for patients with only mild laser burns at least 1,000 um from the center of the fovea performed >6 months preceding Day 1 |
| Mitchell 2011 | 1) ≥18 years of age with either type 1 or 2 diabetes mellitus (as per ADA or WHO guidelines), HbA_1c_ ≤10%, and visual impairment due to DME; 2) Stable medication for the management of diabetes within 3 months before randomization and expected to remain stable during the study; 3) Visual impairment due to focal or diffuse DME in at least 1 eye that was eligible for laser treatment in the opinion of the investigator; 4) BCVA letter score between 78 and 39, both inclusive, based on ETDRS-like VA testing charts administered at a starting distance of 4 m (20/32–20/160 approximate Snellen equivalent); 5) Decreased vision due to DME and not other causes | 1) Concomitant conditions in the study eye that could prevent the improvement in VA on the study treatment in the investigator’s opinion; 2) Active intraocular inflammation or infection in either eye; 3) Uncontrolled glaucoma in either eye; 4) Pan-retinal laser photocoagulation (within 6 months) or focal/grid laser photocoagulation (within 3 months) before study entry; 5) Treatment with anti-angiogenic drugs in the study eye within 3 months before randomization; 6) History of stroke;  7) Systolic BP >160 mmHg or diastolic BP >≥100 mmHg, untreated hypertension, or change in antihypertensive treatment within 3 months preceding baseline |
| Morioka 2018 | 1) Type 2 diabetes; 2) CRT of ≥300 um in the central subfield based on Cirrus OCT due to DME; 3) Leakage from capillary retinal vessels and microaneurysms, corresponding to macular edema, was identified by fluorescein angiography | 1) <20 years of age; 2) Focal/grid photocoagulation or pan-retinal photocoagulation within the previous 6 months; 3) Active intraocular inflammation or infection in either eye; 4) Uncontrolled glaucoma in either eye; 5) A history of intravitreal injections of anti-angiogenic drug or steroids within 3 months of enrollment; 6) A history of stroke; 7) A systolic BP >160 mmHg, a diastolic BP >100 mmHg or untreated hypertension; 8) HbA_1c_ ≥10% |
| Mukkamala 2017 | 1) Age ≥18 years. Diagnosis of type 1 or type 2 diabetes; 2) BCVA ETDRS letter score ≤78 (20/32 or worse) and ≥24 (20/320 or better); 3) Definite retinal thickening on clinical exam due to DME involving the center of the macula; 4) Media clarity, pupillary dilation, and individual cooperation sufficient for adequate fundus photographs; 5) CST on OCT ≥250 µm on Zeiss Stratus; ≥320 if male or ≥305 if female on Heidelberg Spectralis; ≥305 if male or ≥290 if female on Zeiss Cirrus | 1) Significant renal disease, defined as a history of chronic renal failure requiring dialysis or kidney transplant; 2) A condition that, in the opinion of the investigator, would preclude participation (e.g. unstable medical status, including BP, cardiovascular disease, and glycemic control); 3) Participation in an investigational trial within 30 days of randomization that involved treatment with any drug that has not received regulatory approval for the indication being studied at the time of study entry; 4) Known allergy to any component of the study drug; 5) BP >180/110 (systolic above 180 OR diastolic above 110); 6) MI, other acute cardiac event requiring hospitalization, stroke, transient ischemic attack, or treatment for acute congestive heart failure within 4 months prior to randomization; 7) Systemic anti-VEGF or pro-VEGF treatment within 4 months prior to randomization or anticipated use during the study; 8) For women of child-bearing potential: pregnant or lactating or intending to become pregnant within the next 24 months; 9) Individual is expecting to move out of the area of the clinical center to an area not covered by another clinical center during the first 12 months of the study; 10) Macular edema is considered to be due to a cause other than DME; 11) An ocular condition is present such that, in the opinion of the investigator, VA loss would not improve from resolution of macular edema (e.g. foveal atrophy, pigment abnormalities, dense subfoveal hard exudates, or non-retinal condition); 12) An ocular condition is present (other than diabetes) that might affect macular edema or alter VA during the course of the study (e.g. vein occlusion, uveitis or other ocular inflammatory disease, neovascular glaucoma, etc.); 13) A substantial cataract is present that is likely to be decreasing VA by three lines or more (i.e. cataract would be reducing VA to 20/40 or worse if eye was otherwise normal); 14) History of an anti-VEGF treatment for DME in the past 12 months or history of any other treatment for DME at any time in the past 4 months (such as focal/grid macular photocoagulation, intravitreal or peribulbar corticosteroids); 15) History of pan-retinal photocoagulation within 4 months prior to randomization or anticipated need for pan-retinal photocoagulation in the 6 months following randomization; 16) History of anti-VEGF treatment for a disease other than DME in the past 12 months; 17) History of major ocular surgery (including vitrectomy, cataract extraction, scleral buckle, any intraocular surgery, etc.) within prior 4 months or anticipated within the next 6 months following randomization; 18) History of YAG capsulotomy performed within 2 months prior to randomization; 19) Aphakia; 20) Exam evidence of external ocular infection, including conjunctivitis, chalazion, or significant blepharitis |
| Nepomuceno 2013 | 1) Center-involved DME, defined as a CST >300 mm on SD-OCT, despite at least 1 session of macular laser photocoagulation performed at least 3 months previously;  2) BCVA ETDRS measurement between 0.3 logMAR (20/40 Snellen equivalent) and 1.6 logMAR (20/800 Snellen equivalent);  3) Signed informed consent | 1) Vitreo-macular traction on SD-OCT;  2) Proliferative DR needing pan-retinal photocoagulation (PRP) or anticipated to need PRP in the next 12 months;  3) Macular capillary dropout on fluorescein angiography;  4) History of glaucoma or ocular hypertension (defined as an IOP higher than 22 mmHg);  5) An ocular condition (other than diabetes) that, in the opinion of the investigator, might affect macular edema or alter VA during the course of the study (e.g. retinal vein occlusion, uveitis or other ocular inflammatory disease, neovascular glaucoma, etc.);  6) Systemic corticosteroid therapy; 7) Any condition that, in the opinion of the investigator, might preclude follow-up throughout the study period |
| Ozsaygili 2019 | 1) Patients older than 18 years of age diagnosed with type 1 or 2 treatment-naïve DME; 2) Treatment-naïve DME with serous retinal detachment and hyper-reflective foci documented by SD-OCT;  3) BCVA letter score between 73 and 34 (20/40–20/200 Snellen equivalent);  4) The CRT obtained from the 1-mm central macular subfield of the study eye was required to be greater than 450 mm by SD-OCT | 1) Previous history of intraocular anti-VEGF or steroid injection;  2) Evidence of macular ischemia defined by FFA;  3) Any other ocular pathologies causing visual impairment (neovascular AMD, choroidal neovascularization, retinal vein occlusion, uveitis, and recent intraocular surgery);  4) Recent (within 3 months) serious cardiovascular or cerebrovascular events;  5) IOP over 23 mmHg without treatment or IOP over 21 mmHg with one antiglaucoma medication;  6) Presence of vitreo-macular interface abnormalities;  7) Aphakia or an anterior chamber intraocular lens;  8) Active proliferative DR |
| Podkowinski 2020 | 1) Patients from the outpatient clinic presenting clinically significant macular edema secondary to diabetes; 2) Therapeutic intravitreal treatment within the last 3 months or other retinal diseases such as epiretinal membrane, vitreo-macular traction syndrome, retinal atrophy, or glaucoma. Patients were screened for recent cardiovascular events (3 months prior to treatment) and active infectious disease by medical records | NR |
| Qin 2020 | 1) Diagnosed for diabetes;  2) Diagnosed as macular edema by FFA, slit lamp, and OCT, single eye disease, with obvious retinal thickening within 500 mm of the macular center;  3) The patient informed consent | 1) Macular center caused by other reasons, history of eye surgery, history of glaucoma, severe heart, brain and kidney dysfunction, incomplete clinical data, and loss to follow-up |
| Sarda 2020 | 1) Age ≥18 years old; 2) With type 1 or 2 diabetes; 3) CRT ≥300 microns using swept-source OCT, and VA ≤75 letters on the ETDRS scale; 4) Naïve of treatment, without concomitant pan-retinal photocoagulation | 1) Active proliferative DR; 2) Intravitreous hemorrhage or tractional retinal detachment; 3) Ischemic maculopathy, defined by a 2-time enlargement of the foveal avascular zone assessed by fluorescein angiography; 4) Any other ocular pathology contributing to the decrease in VA |
| Scott 2007 | 1) Age ≥18 years; 2) Diagnosis of diabetes mellitus (type 1 or type 2); 3) At least one eye meets the study eye criteria listed below; 4) Fellow eye meets criteria listed below | 1) Significant renal disease, defined as a history of chronic renal failure requiring dialysis or kidney transplant; 2) A condition that, in the opinion of the investigator, would preclude participation in the study (e.g. unstable medical status, including BP, cardiovascular disease, and glycemic control); 3) Participation in an investigational trial within 30 days of randomization that involved treatment with any drug that has not received regulatory approval at the time of study entry; 4) Known allergy to any component of the study drug; 5) BP >180/110 (systolic above 180 OR diastolic above 110); 6) Major surgery within 28 days prior to randomization or major surgery planned during the next 6 months; 7) MI, other cardiac event requiring hospitalization, stroke, transient ischemic attack, or treatment for acute congestive heart failure within 6 months prior to randomization; 8) Systemic anti-VEGF or pro-VEGF treatment within 3 months prior to randomization; 9) For women of child-bearing potential: pregnant or lactating or intending to become pregnant within the next 6 months; 10) Subject is expecting to move out of the area of the clinical center to an area not covered by another clinical center during the first 6 months of the study |
| Soheilian 2009 | 1) Patients with clinically significant DME based on ETDRS criteria | 1) Previous pan-retinal or focal laser photocoagulation, prior intraocular surgery or injection, history of glaucoma or ocular hypertension; 2) VA of 20/40 or better or worse than 20/300;  3) Presence of iris neovascularization, high-risk proliferative DR, and significant media opacity; 4) Monocularity, pregnancy, serum creatinine ≥3 mg/dL, and uncontrolled diabetes mellitus |
| Solaiman 2010 | 1) Have diffuse DME with fluorescein angiographic evidence and OCT CMT ≥350 um; 2) No history of intravitreal injection, surgical intervention, or retinal laser therapy for DR; 3) Willingness to attend follow-up visits for at least 6 months | 1) Cystoid macular edema, vitreoretinal traction involving the macular region, macular, vitreous incarceration in a previous wound or in vision, opacity of the optical media as cataract or vitreous hemorrhage; 2) History of intraocular surgery during the previous year; 3) Chronic uveitis and retinal vein occlusion involving the macular region |
| Vader 2020 | 1) Age ≥18 years old; 2) With type 1 or 2 diabetes; 3) With HbA_1c_ <12%; 4) Central area thickness on OCT >325 um; 5) BCVA of at least 24 letters and less than 79 letters on standardized ETDRS charts; 6) Has given written informed consent | 1) Women of child-bearing potential, pregnant or nursing women; 2) Active intraocular inflammation or any active infection in either eye; 3) Structural damage within 600 µm of the center of the macula in the study eye likely to preclude VA improvement following resolution of the macular edema;  4) Uncontrolled glaucoma, neovascularization of the iris, vitreo-macular traction, active untreated proliferative DR in the study eye; 5) Any intraocular surgery, focal/grid laser photocoagulation, pan-retinal laser photocoagulation, planned medical or surgical intervention in the study eye prior to or during the study; 6) History of vitrectomy in the study eye;  7) Treatment with anti-angiogenic drugs, use of other investigational drugs in the study eye prior to study;  8) History of intravitreal or subconjunctival corticosteroids in phakic eye within 18 months  prior to screening or in post-cataract surgery study eye within 43 months prior to screening; 9) Ocular conditions in the study eye that require chronic concomitant therapy with topical  ocular or systemically administered corticosteroids; 10) History of stroke or transient ischemic attack within 6 months prior to screening; 11) Renal failure requiring dialysis or renal transplant or renal insufficiency with creatinine  levels >2.0 mg/dL at screening;  12) BP systolic >165 mmHg or diastolic >105 mmHg; 13) Hypertension or change in antihypertensive treatment within 1 month preceding  screening; 14) Current use of or likely need for systemic medications known to be toxic to the lens,  retina or optic nerve;  15) Known hypersensitivity to fluorescein, ranibizumab or bevacizumab or any component  thereof or drugs of similar chemical classes; 16) Any type of advanced, severe or unstable disease or its treatment, that may interfere  with primary and/or secondary variable evaluations; 17) Concomitant conditions in the study eye that would prevent the improvement of VA on study treatment; 18) Ocular disorders in the study eye that may confound interpretation of study results,  compromise VA or require medical or surgical intervention during the 6-month  study period |
| Wiley 2016 | 1) Patients with type 1 or type 2 diabetes mellitus; 2) Were at least 18 years old; 3) Presence of DME involving the center of the macula;  4) BCVA ETDRS letter score 78–24 (20/32–20/400 Snellen equivalent);  5) Mean CST of ≥330 μm on Cirrus (Carl Zeiss Meditec) OCT | 1) Presence of factors or other conditions judged to impact the course of edema or preclude possible improvement in vision with treatment;  2) Pan-retinal photocoagulation, focal/grid laser photocoagulation, or depot corticosteroid injection within the previous 3 months;  3) Ocular injection with an anti-VEGF agent within the previous 2 months;  4) More than 4 injections with an anti-VEGF agent within the previous year;  5) Prior vitrectomy; 6) History of renal failure (requiring hemodialysis or renal transplant) and for a measured systolic BP of >180 mmHg or diastolic BP of >110 mmHg |
| Wu 2020 | 1) Diagnosed with type 2 diabetes after clinical diagnosis. Slit-lamp microscopy showed macular edema, and diagnosed with severe DME; 2) Complete examination and medical history data of the patient can be collected; 3) The research was approved by the hospital medical ethics committee | 1) History of glaucoma and severe hypertension; 2) Received eye surgery within 6 months before treatment; 3) Other ocular diseases or diseases affecting the study; 4) Adverse reactions to the method of this study or those who are unwell; 5) Do not comply with, cooperate with, and refuse to participate in this study |
| Xiang 2018 | NR | 1) Cardiovascular and cerebrovascular diseases, immune diseases, and mental health diseases |
| Yan 2017 | Diagnosed as retinopathy with macular edema by routine eye examination, fundus photography, ophthalmology B-mode ultrasound, macular OCT and FFA | 1) Other macular diseases, such as the premacular membrane, glaucoma, etc. |
| Yang 2018 | 1) History of diabetes;  2) VA <0.05;  3) DME resulted in decreased vision and increased fovea thickness | 1) Laser photocoagulation was performed in the past 6 months;  2) IOP ≥25 mmHg;  3) Other causes leading to macular edema;  4) Systolic BP escape ≥180 mmHg or diastolic BP escape ≥110 mmHg;  5) Previous history of MI or CVA;  6) Cardiac, cerebral, liver and kidney dysfunction;  7) Mental health diseases;  8) Pregnant and lactating women;  9) Incomplete clinical data |
| Yang 2020 | NR | NR |
| Yu 2018 | 1) Those who are willing to perform according to the requirements of the clinical trial method; 2) OCT examination of the CRT ≥250 μm; 3) Stage III DR patients who have not received macular, retinal photocoagulation, or intravitreal injection of drugs; 4) The refractive interstitium is transparent and the examination is fully cooperative | Other causes of macular edema except DME |
| Zheng 2017 | 1) Type 2 diabetes, which meets the diagnostic criteria in the Diabetes Diagnosis and Treatment Guidelines; 2) Meets the focal DME in the Bresnick classification and clinically significant DME in the ETDRS classification; 3) In the follow-up, remove the missing cases; 4) HbA_1c_ <6%; 5) The vision is between 0.06 and 0.5 | 1) High myopia greater than or equal to 6D; 2) AMD; 3) IOP ≥25 mmHg; 4) Patients with intraocular surgery in the past 3 months; 5) Other reasons; 6) Retinal photocoagulation in the past 4 months or retinal photocoagulation in the next 6 months; 7) Combined with other diseases of the eye and systemic system, such as MI, CVA, etc. |

## Table S3 Characteristics of Interventions

| **Study ID** | **Subgroup or not at baseline? (Worse baseline VA/Non-worse baseline VA/Treatment naïve/Formerly treated)** | **Description of Intervention** | **Dosage of Intervention** | **Frequency of Intervention** | **PRN or not** | **Timepoint:**  **Number of treatments received, mean (SD)** |
| --- | --- | --- | --- | --- | --- | --- |
| Azad 2012 | All patients were formerly treated | IVB | 1.25 mg | Baseline, then PRN | Yes | 6 m: 2.7 (0.4) |
| Azad 2012 | All patients were formerly treated | Laser | NR | Baseline, then PRN | Yes | 6 m: 1.8 (0) |
| Baker 2019 | All patients were non-worse baseline VA | IVA | 2 mg | Baseline, then PRN | Yes | 1 y: 6 (2.5) |
| Baker 2019 | All patients were non-worse baseline VA | IVA | 2 mg | Baseline, then PRN | Yes | 2 y: 2.4 (2.7) |
| Baker 2019 | All patients were non-worse baseline VA | Laser | NR | Baseline, then PRN | Yes | 1 y: 0.7 (2.1) |
| Baker 2019 | All patients were non-worse baseline VA | Laser | NR | Baseline, then PRN | Yes | 2 y: 1.3 (2.7) |
| Berger 2015 | No | IVR | 0.5 mg | 3-monthly loading, then PRN | Yes | 1 y: 9.2 (2.8) |
| Berger 2015 | No | Laser | NR | Baseline, then PRN | Yes | 1 y: 2.6 (2.1) |
| Boyer 2014 | All patients were worse baseline VA | Dex | 0.7 mg | Every 1.5 months during the first year and every 3 months during Years 2 and 3 | No | 3 y: 4.1 (2) |
| Boyer 2014 | All patients were worse baseline VA | Dex | 0.35 mg | Every 1.5 months during the first year and every 3 months during Years 2 and 3 | No | 3 y: 4.4 (1.9) |
| Boyer 2014 | All patients were worse baseline VA | Sham | NR | Every 1.5 months during the first year and every 3 months during Years 2 and 3 | No | 3 y: 3.3 (2.2) |
| Boyer 2014 | Formerly treated and worse baseline VA | Dex | 0.7 mg | Every 1.5 months during the first year and every 3 months during Years 2 and 3 | No | NR |
| Boyer 2014 | Formerly treated and worse baseline VA | Sham | NR | Every 1.5 months during the first year and every 3 months during Years 2 and 3 | No | NR |
| Brown 2013-RIDE | All patients were worse baseline VA | Sham | NA | Monthly | No | NR |
| Brown 2013-RIDE | All patients were worse baseline VA | IVR | 0.3 mg | Monthly | No | NR |
| Brown 2013-RIDE | All patients were worse baseline VA | IVR | 0.5 mg | Monthly | No | NR |
| Brown 2013-RISE | All patients were worse baseline VA | Sham | NA | Monthly | No | NR |
| Brown 2013-RISE | All patients were worse baseline VA | IVR | 0.3 mg | Monthly | No | NR |
| Brown 2013-RISE | All patients were worse baseline VA | IVR | 0.5 mg | Monthly | No | NR |
| Brown 2015-VISTA | All patients were worse baseline VA | IVA | 2 mg | Monthly | No | 1 y: 11.8 (2.6) |
| Brown 2015-VISTA | All patients were worse baseline VA | IVA | 2 mg | 5-monthly loading, then bi-monthly | No | 1 y: 8.4 (1.3) |
| Brown 2015-VISTA | All patients were worse baseline VA | Laser | NR | Baseline, then PRN | Yes | 1 y: 2.7 (1.1) |
| Brown 2015-VISTA | All patients were worse baseline VA | IVA | 2 mg | Monthly | No | 2 y: 21.3 (5.8) |
| Brown 2015-VISTA | All patients were worse baseline VA | IVA | 2 mg | 5-monthly loading, then bi-monthly | No | 2 y: 13.5 (2.9) |
| Brown 2015-VISTA | All patients were worse baseline VA | Laser | NR | Baseline, then PRN | Yes | 2 y: 3.5 (2) |
| Brown 2015-VIVID | All patients were worse baseline VA | IVA | 2 mg | Monthly | No | 1 y: 12.2 (2.6) |
| Brown 2015-VIVID | All patients were worse baseline VA | IVA | 2 mg | 5-monthly loading, then bi-monthly | No | 1 y: 8.7 (1.2) |
| Brown 2015-VIVID | All patients were worse baseline VA | Laser | NR | Baseline, then PRN | Yes | 1 y: 2.1 (1.1) |
| Brown 2015-VIVID | All patients were worse baseline VA | IVA | 2 mg | Monthly | No | 2 y: 22.6 (5.8) |
| Brown 2015-VIVID | All patients were worse baseline VA | IVA | 2 mg | 5-monthly loading, then bi-monthly | No | 2 y: 13.6 (2.9) |
| Brown 2015-VIVID | All patients were worse baseline VA | Laser | NR | Baseline, then PRN | Yes | 2 y: 2.4 (1.6) |
| Callanan 2016 | All patients were worse baseline VA | Dex | 0.7 mg | Every 5 months | No | Median (range) 1 y: 3 (1–3) |
| Callanan 2016 | All patients were worse baseline VA | IVR | 0.5 mg | 3-monthly loading, then PRN | Yes | Median (range) 1 y: 9 (2–12) |
| Chatzirallis 2020 | All patients were treatment naïve | IVR | 0.5 mg | 3-monthly loading, then PRN | Yes | 1.5 y: 9.2 (2.3) |
| Chatzirallis 2020 | All patients were treatment naïve | IVA | 2 mg | 3-monthly loading, then PRN | Yes | 1.5 y: 7.6 (2.1) |
| Chen 2020 | All patients were worse baseline VA | IVA | 2 mg | Monthly | No | 1 y: 12.6 (1.9) |
| Chen 2020 | All patients were worse baseline VA | IVA | 2 mg | 5-monthly, then bi-monthly | No | 1 y: 8.7 (1.1) |
| Chen 2020 | All patients were worse baseline VA | Laser | NR | Baseline, then PRN | Yes | 1 y: 2.4 (1.1) |
| Comyn 2014 | No | IVR | 0.5 mg | 3-monthly loading, then PRN | Yes | 1 y: 9 |
| Comyn 2014 | No | Laser | NR | PRN | Yes | 1 y: 2.6 |
| Do 2013 | All patients were worse baseline VA | IVR | 0.5 mg | Bi-monthly, then PRN | Yes | 3 y: 5.4 (IVR injections in Year 3) |
| Do 2013 | All patients were worse baseline VA | Laser | NR | Baseline, then PRN | Yes | 3 y: 2.3 |
| Du 2017 | No | IVR | 0.5 mg | Monthly | No | NR |
| Du 2017 | No | Laser | Laser or grid photocoagulation | NR | NR | NR |
| Fan 2019 | All patients were treatment naïve | IVC | 0.05 mL | 3-monthly | No | NR |
| Fan 2019 | All patients were treatment naïve | Laser | 100 μm | Baseline, then PRN | Yes | NR |
| Fouda 2017 | No | IVA | 2 mg | 3-monthly loading, then PRN | Yes | 1 y: 2.62 (0.68) |
| Fouda 2017 | No | IVR | 0.5 mg | 3-monthly loading, then PRN | Yes | 1 y: 3.03 (0.95) |
| Gillies 2014 | All patients were worse baseline VA | IVB | 1.25 mg | Baseline, then monthly PRN | Yes | 1 y: 8.6 (mean) |
| Gillies 2014 | All patients were worse baseline VA | Dex | 0.7 mg | Baseline, then 4-monthly PRN | Yes | 1 y: 2.3 (mean) |
| Gu 2015 | All patients were treatment naïve and worse baseline VA | Laser | 500 μm, “C”-shaped macular grid photocoagulation wavelength of 577 nm | Baseline | No | NR |
| Gu 2015 | All patients were treatment naïve and worse baseline VA | IVR | 1.25 mg | Baseline | No | NR |
| Jiang 2015 | No | Laser | 100 μm, 150–200 mW, 0.1 ms | Baseline | No | NR |
| Jiang 2015 | No | IVR | 0.5 mg | Baseline | No | NR |
| Li 2015 | All patients were worse baseline VA | Laser | 568 nm, 50 μm, 0.1–0.15 s, 100–230 mW | Baseline | No | NR |
| Li 2015 | All patients were worse baseline VA | IVR | 10 g/L | Baseline | No | NR |
| Li 2019 | No | IVR | 0.5 mg | 3-monthly loading, then PRN | Yes | 1 y: 7.9 (2.82) |
| Li 2019 | No | Laser | NR | Baseline, then PRN | Yes | 1 y: 2.1 (1.08) |
| Liu 2019 | All patients were worse baseline VA | IVC | 0.5 mg | Baseline, then PRN | Yes | 1y: 9.5 |
| Liu 2019 | All patients were worse baseline VA | Laser | NR | PRN | Yes | 1y: 2.7 |
| Massin 2010 | All patients were worse baseline VA | IVR | 0.3 mg | 3-monthly loading, then PRN | Yes | 1 y: 10.2 (2.5) |
| Massin 2010 | All patients were worse baseline VA | IVR | 0.5 mg | 3-monthly loading, then PRN | Yes | 1 y: 10.2 (2.5) |
| Massin 2010 | All patients were worse baseline VA | Sham | 0.05 mL | 3-monthly loading, then PRN | Yes | 1 y: 8.9 (3.5) |
| Mitchell 2011 | No | IVR | 0.5 mg | 3-monthly loading, then PRN | Yes | 1 y: 7 (2.81) |
| Mitchell 2011 | No | Laser | NR | Baseline, then PRN | Yes | 1 y: 2.1 (1.04) |
| Mitchell 2011 | No | IVR | 0.5 mg | 3-monthly loading, then PRN | Yes | 3 y: 14.2 (7.9) |
| Mitchell 2011 | No | Laser | NR | Baseline, then PRN | Yes | 3 y: 2.3 (1.5) |
| Mitchell 2011 | Worse baseline VA | IVR | NR | Baseline, then PRN | Yes | NR |
| Mitchell 2011 | Worse baseline VA | Laser | NR | Baseline, then PRN | Yes | NR |
| Mitchell 2011 | Non-worse baseline VA | IVR | NR | Baseline, then PRN | Yes | NR |
| Mitchell 2011 | Non-worse baseline VA | Laser | NR | Baseline, then PRN | Yes | NR |
| Morioka 2018 | Phakic | IVR | 0.5 mg | Baseline | No | 3 m (total): 1 |
| Morioka 2018 | Phakic | IVA | 2 mg | Baseline | No | 3 m (total): 1.025 |
| Morioka 2018 | Pseudophakic | IVR | 0.5 mg | Baseline | No | 3 m (total): 1 |
| Morioka 2018 | Pseudophakic | IVA | 2 mg | Baseline | No | 3 m (total): 1.025 |
| Mukkamala 2017 | No | IVR | 0.3 mg | Baseline, then monthly or PRN | Yes | 1 y: 9.4 (2.1) |
| Mukkamala 2017 | No | IVA | 2 mg | Baseline, then monthly or PRN | Yes | 1 y: 9.2 (2.0) |
| Mukkamala 2017 | No | IVB | 1.25 mg | Baseline, then monthly or PRN | Yes | 1 y: 9.7 (2.3) |
| Mukkamala 2017 | No | IVR | 0.3 mg | Baseline, then monthly or PRN | Yes | 2 y (injections in Year 2), median (IQR): 6 (2–9) |
| Mukkamala 2017 | No | IVA | 2 mg | Baseline, then monthly or PRN | Yes | 2 y (injections in Year 2), median (IQR): 5 (2–7) |
| Mukkamala 2017 | No | IVB | 1.25 mg | Baseline, then monthly or PRN | Yes | 2 y (injections in Year 2), median (IQR): 6 (2–9) |
| Mukkamala 2017 | Worse baseline VA | IVR | 0.3 mg | Baseline, then monthly or PRN | Yes | 1 y: 9.7 (1.9) |
| Mukkamala 2017 | Worse baseline VA | IVA | 2 mg | Baseline, then monthly or PRN | Yes | 1 y: 9.6 (2.1) |
| Mukkamala 2017 | Worse baseline VA | IVB | 1.25 mg | Baseline, then monthly or PRN | Yes | 1 y: 10.4 (2.0) |
| Mukkamala 2017 | Non-worse baseline VA | IVR | 0.3 mg | Baseline, then monthly or PRN | Yes | 1 y: 9.1 (2.3) |
| Mukkamala 2017 | Non-worse baseline VA | IVA | 2 mg | Baseline, then monthly or PRN | Yes | 1 y: 8.7 (1.9) |
| Mukkamala 2017 | Non-worse baseline VA | IVB | 1.25 mg | Baseline, then monthly or PRN | Yes | 1 y: 9 (2.5) |
| Mukkamala 2017 | Worse baseline VA | IVA | 2 mg | Baseline, then monthly or PRN | Yes | 2 y: 15.2 (4.7) |
| Mukkamala 2017 | Worse baseline VA | IVB | 1.25 mg | Baseline, then monthly or PRN | Yes | 2 y: 17 (4.8) |
| Mukkamala 2017 | Worse baseline VA | IVR | 0.3 mg | Baseline, then monthly or PRN | Yes | 2 y: 15.9 (4.7) |
| Mukkamala 2017 | Non-worse baseline VA | IVR | 0.3 mg | Baseline, then monthly or PRN | Yes | 2 y: 13.8 (5.2) |
| Mukkamala 2017 | Non-worse baseline VA | IVA | 2 mg | Baseline, then monthly or PRN | Yes | 2 y: 13.2 (4.2) |
| Mukkamala 2017 | Non-worse baseline VA | IVB | 1.25 mg | Baseline, then monthly or PRN | Yes | 2 y: 13.5 (5.3) |
| Nepomuceno 2013 | All patients were worse baseline VA and formerly treated | IVR | 0.5 mg | Baseline, then PRN | Yes | 1 y: 7.67 (0.6) |
| Nepomuceno 2013 | All patients were worse baseline VA and formerly treated | IVB | 1.5 mg | Baseline, then PRN | Yes | 1 y: 9.84 (0.55) |
| Ozsaygili 2019 | All patients were treatment naïve | Dex | 0.7 mg | 3-monthly loading, then PRN | Yes | 1 y: 2.6 |
| Ozsaygili 2019 | All patients were treatment naïve | IVA | 2 mg | 3-monthly loading, then PRN | Yes | 1 y: 7.2 |
| Podkowinski 2020 | No | IVR | 0.5 mg | 4-monthly loading, then PRN | Yes | NR |
| Podkowinski 2020 | No | Dex | NR | Baseline | No | NR |
| Qin 2020 | All patients were worse baseline VA | IVR | 0.02 mL | 3-monthly | No | NR |
| Qin 2020 | All patients were worse baseline VA | IVC | 0.02 mL | 3-monthly | No | NR |
| Sarda 2020 | All patients were treatment naïve | IVA | 2 mg | 5-monthly | No | NR |
| Sarda 2020 | All patients were treatment naïve | IVR | 0.5 mg | 5-monthly | No | NR |
| Scott 2007 | No | Laser | Focal photocoagulation | Baseline, then PRN after 12 weeks | Yes | NR |
| Scott 2007 | No | IVB | 1.25 mg | Baseline and 6 weeks, then PRN after 12 weeks | Yes | NR |
| Scott 2007 | No | IVB | 2.5 mg | Baseline and 6 weeks, then PRN after 12 weeks | Yes | NR |
| Scott 2007 | No | IVB | 1.25 mg | Baseline, then PRN after 12 weeks | Yes | NR |
| Soheilian 2009 | All patients were treatment naïve and worse baseline VA | IVB | 1.25 mg | Baseline, then PRN at 12-week intervals | Yes | 2 y: 3.1 (1.6) |
| Soheilian 2009 | All patients were treatment naïve and worse baseline VA | Laser | Standard focal or modified grid laser was performed | Baseline, then PRN at 12-week intervals | Yes | 2 y: 1 (0.1) |
| Solaiman 2010 | All patients were treatment naïve | Laser | 2–3 row of 100 μm spots, 100 μm apart in the parafoveal region; 150–200 μm sports, 200 μm apart to the remaining areas of retinal thickening and capillary nonperfusion; 100–150 μm sports in focal leaks outside or within the zones of diffuse leakage | Baseline | No | NR |
| Solaiman 2010 | All patients were treatment naïve | IVB | 1.25 mg | Baseline | No | NR |
| Vader 2020 | No | IVB | 1.25 mg | 6-monthly | No | 6 m: 5.95 (0.03) |
| Vader 2020 | No | IVR | 0.5 mg | 6-monthly | No | 6 m: 5.98 (0.02) |
| Vader 2020 | Worse baseline VA | IVB | 1.25 mg | 6-monthly | No | NR |
| Vader 2020 | Worse baseline VA | IVR | 0.5 mg | 6-monthly | No | NR |
| Vader 2020 | Non-worse baseline VA | IVB | 1.25 mg | 6-monthly | No | NR |
| Vader 2020 | Non-worse baseline VA | IVR | 0.5 mg | 6-monthly | No | NR |
| Wiley 2016 | No | IVB | 1.25 mg | 3-monthly loading, then PRN | Yes | NR |
| Wiley 2016 | No | IVR | 0.3 mg | 3-monthly loading, then PRN | Yes | NR |
| Wiley 2016 | Worse baseline VA | IVB | 1.25 mg | 12-week treatment | No | NR |
| Wiley 2016 | Worse baseline VA | IVR | 0.3 mg | 12-week treatment | No | NR |
| Wiley 2016 | Non-worse baseline VA | IVB | 1.25 mg | 12-week treatment | No | NR |
| Wiley 2016 | Non-worse baseline VA | IVR | 0.3 mg | 12-week treatment | No | NR |
| Wu 2020 | No | IVR | 0.05 mL | 2-monthly | No | NR |
| Wu 2020 | No | Laser | 577 nm | Baseline | No | NR |
| Xiang 2018 | No | IVC | 0.5 mg | 3-monthly | No | NR |
| Xiang 2018 | No | IVR | 0.5 mg | 3-monthly | No | NR |
| Yan 2017 | All patients were worse baseline VA | IVC | 0.5 mg | Baseline | No | NR |
| Yan 2017 | All patients were worse baseline VA | Laser | 532 nm PRP | Baseline | No | NR |
| Yang 2018 | All patients were worse baseline VA | Laser | 568 nm | Baseline | No | NR |
| Yang 2018 | All patients were worse baseline VA | IVR | 0.2 mL | 3-monthly then PRN | Yes | NR |
| Yang 2020 | No | IVR | 0.5 mg | 3-monthly loading, then PRN | Yes | NR |
| Yang 2020 | No | IVC | 0.5 mg | 3-monthly loading, then PRN | Yes | NR |
| Yu 2018 | All patients were treatment naïve | IVR | NR | 2-monthly | No | 1 y: 5.7 (1.23) |
| Yu 2018 | All patients were treatment naïve | Laser | PRP | Baseline | No | 1 y: 3 (1) |
| Zheng 2017 | All patients were worse baseline VA | IVR | 0.5 mg | Baseline | No | NR |
| Zheng 2017 | All patients were worse baseline VA | Laser | 100 μm grid photocoagulation | Baseline | No | NR |

Dex, dexamethasone implant; IVA, aflibercept; IVB, bevacizumab; IVC, conbercept; IVR, ranibizumab; NA, not available; NR, not reported; PRN, pro re nata; PRP, pan-retinal photocoagulation; SD, standard deviation; Sham, placebo

# Appendix 3 Reference list of included RCTs (Citations for studies in Table 1)

**Azad 2012**

Azad, R., et al., Comparison of IVB, intravitreal triamcinolone acetonide, and macular grid augmentation in refractory diffuse diabetic macular edema: a prospective, randomized study. Oman Journal of Ophthalmology, 2012. 5(3): p. 166-170.

**Baker 2019**

Baker, C.W., et al., Effect of initial management with aflibercept vs laser photocoagulation vs observation on vision loss among patients with diabetic macular edema involving the center of the macula and good visual acuity: a randomized clinical trial. JAMA, 2019. 321(19): p. 1880-1894.

**Berger 2015**

Berger, A., et al., Efficacy/safety of ranibizumab monotherapy or with laser versus laser monotherapy in DME. Canadian Journal of Ophthalmology-Journal Canadien D Ophtalmologie, 2015. 50(3): p. 209-216.

**Boyer 2014**

Boyer, D.S., et al., Three-year, randomized, sham-controlled trial of dexamethasone intravitreal implant in patients with diabetic macular edema. Ophthalmology, 2014. 121(10): p. 1904-14.

**Brown-RIDE and RISE**

Brown, D.M., et al., Long-term outcomes of ranibizumab therapy for diabetic macular edema: the 36-month results from two phase III trials: RISE and RIDE. Ophthalmology, 2013. 120(10): p. 2013-22.

**Brown-VISTA and VIVID**

Brown, D.M., et al., IVT-AFL for diabetic macular edema: 100-week results from the VISTA and VIVID studies. Ophthalmology, 2015. 122(10): p. 2044-52.

**Callanan 2017**

Callanan, D.G., et al., A multicenter, 12-month randomized study comparing dexamethasone intravitreal implant with ranibizumab in patients with diabetic macular edema. Graefe's Archive for Clinical and Experimental Ophthalmology = Albrecht von Graefes Archiv fur klinische und experimentelle Ophthalmologie, 2017. 255(3): p. 463-473.

**Chatzirallis 2020**

Chatzirallis, A., et al., Ranibizumab versus aflibercept for diabetic macular edema: 18-month results of a comparative, prospective, randomized study and multivariate analysis of visual outcome predictors. Cutaneous and Ocular Toxicology, 2020 Dec;39(4):317-322.

**Chen 2020**

Chen, Y.X., et al., IVT-AFL versus laser photocoagulation in Asian patients with diabetic macular edema: the VIVID-East Study. Clinical Ophthalmology, 2020. 14: p. 741-750.

**Comyn 2014**

Comyn, O., et al., A randomized trial to assess functional and structural effects of ranibizumab versus laser in diabetic macular edema (the LUCIDATE study). American Journal of Ophthalmology, 2014. 157(5): p. 960-70.

**Do 2013**

Do, D.V., et al., Ranibizumab for edema of the macula in diabetes study: 3-year outcomes and the need for prolonged frequent treatment. JAMA Ophthalmology, 2013. 131(2): p. 139-45.

**Du 2017**

Du, F.Q., et al. Effect of intravitreal injection of ranibizumab on choroid in patients with severe NPDR with macular edema [玻璃体内注射雷珠单抗对重度NPDR黄斑水肿患者脉络膜的影响]. 国际眼科杂志 [International Eye Science] 2017. 17(11): p. 2097-2100.

**Fan 2019**

Fan, J.M. Clinical analysis of vitreous cavity injection of anti-VEGF drugs combined with laser for the treatment of diabetic macular oedema [玻璃体腔注射抗VEGF药物联合激光治疗糖尿病性黄斑水肿的临床分析]. 糖尿病新世界 [Diabetes New World] 2019. 22(17): p. 178-179+182.

**Fouda 2017**

Fouda, S.M. and A.M. Bahgat, IVT-AFL versus IVR for the treatment of diabetic macular edema. Clinical Ophthalmology, 2017. 11: p. 567-571.

**Gillies 2014**

Gillies, M.C., et al., A randomized clinical trial of IVB versus intravitreal dexamethasone for diabetic macular edema: the BEVORDEX study. Ophthalmology, 2014. 121(12): p. 2473-81.

**Gu 2015**

Gu, Y.X., et al., Efficacy of macular grating-like photocoagulation versus vitreous cavity injection of ranibizumab in the treatment of diabetic macular oedema in Uyghurs [黄斑区格栅样光凝与玻璃体腔注射雷珠单抗治疗维吾尔族糖尿病黄斑水肿的疗效对比]. 中国急救医学 [Chinese Journal of Critical Care Medicine] 2015. 35(z2): p. 370-372.

**Jiang 2015**

Jiang, C., et al, Clinical efficacy of ranibizumab combined with laser therapy in the treatment of elderly diabetes macular edema [雷珠单抗联合激光治疗老年糖尿病性黄斑水肿的临床效果观察]. 中国医学前沿杂志(电子版) [Chinese Journal of the Frontiers of Medical Science(Electronic Version)] 2015. 7(12): p. 98-100.

**Li 2015**

Li, S.H., et al, Efficacy of macular focal/grid photocoagulation combined with ranibizumab for diabetic macular edema [比较单独黄斑局灶/格栅样光凝术和联合雷珠单抗治疗糖尿病性黄斑水肿的疗效]. 眼科新进展 [Recent Advances in Ophthalmology] 2015. 35(06): p. 566-568+575.

**Li 2019**

Li, X., et al., Efficacy and safety of ranibizumab 0.5 mg in Chinese patients with visual impairment due to diabetic macular edema: results from the 12-month REFINE study. Graefe's Archive for Clinical and Experimental Ophthalmology = Albrecht von Graefes Archiv fur klinische und experimentelle Ophthalmologie, 2019. 257(3): p. 529-541.

**Liu 2021**

Liu K, et al. Intravitreal conbercept for diabetic macular oedema: 2-year results from a randomised controlled trial and open-label extension study. Br J Ophthalmol. 2021 May 17: bjophthalmol-2020-318690. doi: 10.1136/bjophthalmol-2020-318690. Epub ahead of print. PMID: 34001667.

**Massin 2010**

Massin, P., et al., Safety and efficacy of ranibizumab in diabetic macular edema (RESOLVE Study): a 12-month, randomized, controlled, double-masked, multicenter phase II study. Diabetes Care, 2010. 33(11): p. 2399-405.

**Mitchell 2011**

Mitchell, P., et al., The RESTORE study: ranibizumab monotherapy or combined with laser versus laser monotherapy for diabetic macular edema. Ophthalmology, 2011. 118(4): p. 615-25.

**Morioka 2018**

Morioka, M., et al., Flare levels after intravitreal injection of ranibizumab, aflibercept, or triamcinolone acetonide for diabetic macular edema. Graefe's Archive for Clinical and Experimental Ophthalmology = Albrecht von Graefes Archiv fur klinische und experimentelle Ophthalmologie, 2018. 256(12): p. 2301-2307.

**Mukkamala 2017**

Mukkamala, L., N. Bhagat, and M. Zarbin, Practical lessons from protocol T for the management of diabetic macular edema. Developments in Ophthalmology, 2017. 60: p. 109-124.

**Nepomuceno 2013**

Nepomuceno, A.B., et al., A prospective randomized trial of IVB versus ranibizumab for the management of diabetic macular edema. American Journal of Ophthalmology, 2013. 156(3): p. 502-510.e2.

**Ozsaygili 2019**

Ozsaygili, C. and N. Duru, Comparison of intravitreal dexamethasone implant and aflibercept in patients with treatment-naive diabetic macular edema with serous retinal detachment. Retina-the Journal of Retinal and Vitreous Diseases, 2020. 40(6): p. 1044-1052.

**Podkowinski 2020**

Podkowinski, D., et al., Aqueous humour cytokine changes during a loading phase of IVR or dexamethasone implant in diabetic macular oedema. Acta Ophthalmologica, 2020;98(4):e407-e415..

**Qin 2020**

Qin, S.Y., et al., Clinical study on the treatment of IVCIVC for diabetic macular edema [康柏西普玻璃体腔注射治疗糖尿病黄斑水肿的临床研究]. 临床研究 [Clinical Research] 2020. 28(05): p. 60-62.

**Sarda 2020**

Sarda, V., et al., Comparison of the effect of ranibizumab and aflibercept on changes in macular choroidal thickness in patients treated for diabetic macular edema. Journal of Ophthalmology, 2020 Aug 11;2020:5708354. doi: 10.1155/2020/5708354. eCollection 2020.

**Scott 2008**

Scott, I.U., A phase 2 randomized clinical trial of IVB for diabetic macular edema. The Macula Society, 2008: p. 228.

**Soheilian 2009**

Soheilian, M., et al., Randomized trial of IVB alone or combined with triamcinolone versus macular photocoagulation in diabetic macular edema. Ophthalmology, 2009. 116(6): p. 1142-50.

**Solaiman 2010**

Solaiman, K.A.M., M.M. Diab, and M. Abo-Elenin, IVB and/or macular photocoagulation as a primary treatment for diffuse diabetic macular edema. Retina (Philadelphia, Pa.), 2010. 30(10): p. 1638-45.

**Vader 2020**

Vader, M.J.C., et al., Comparing the efficacy of bevacizumab and ranibizumab in patients with diabetic macular edema (BRDME): the BRDME study, a randomized trial. Ophthalmology Retina, 2020. 4(8): p. 777-788.

**Wiley 2016**

Wiley, H.E., et al., A crossover design for comparative efficacy: a 36-week randomized trial of bevacizumab and ranibizumab for diabetic macular edema. Ophthalmology, 2016. 123(4): p. 841-9.

**Wu 2020**

Wu, J.Y., Clinical effect of 577 nm micro pulse laser in the treatment of patients with diabetic severe macular edema [577 nm微脉冲激光治疗糖尿病重度黄斑水肿患者的临床效果]. 中国医药指南 [Guide of China Medicine] 2020. 18(22): p. 116-117.

**Yan 2017**

Yan, Z.Y., et al., Clinical results of intravitreal injection of compazepine in the treatment of diabetic retinopathy with macular oedema [康柏西普玻璃体腔注射治疗糖尿病视网膜病变伴黄斑水肿的临床效果]. 山东医药 [Shandong Medical Journal] 2017. 57(37): p. 57-59.

**Yang 2018**

Yang, D.Y., C.Y. Wang, and X.X. Chong, Clinical study of laser photocoagulation combined with VEGF antagonists for DME. International Eye Science, 2018. 18(6): p. 1014-1017.

**Yang 2020**

Yang, X.P., et al, Effect of ralizumab and intravitreal injection of compazepine on visual acuity and macular thickness in patients with diabetic macular oedema [雷珠单抗与康柏西普玻璃体腔注射对糖尿病黄斑水肿患者视力及黄斑厚度的影响]. 哈尔滨医药 [Harbin Medical Journal] 2020. 40(03): p. 259-260.

**Yu 2018**

Yu, X.L., et al, Clinical effect of treatment with a combination of Lucentis intravitreal injection and whole retinal and macular photo coagulation in patients with diabetic macular Edema [雷珠单抗玻璃体内注射、全视网膜光凝和黄斑光凝联合治疗糖尿病黄斑水肿的临床疗效]. 中国医科大学学报 [Journal of China Medical University], 2018. 47(11): p. 1011-1014.

**Zheng 2017**

Zheng, Q.P., et al., Efficacy of ranibizumab alone or in combination with macular grating laser in diabetic macular oedema [雷珠单抗单独或联合黄斑格栅激光对糖尿病黄斑水肿的疗效]. 牡丹江医学院学报 [Journal of Mudanjiang Medical University] 2017. 38(03): p. 38-39+156.

# Appendix 4 Unit of analysis issues

## Mean change in best-corrected visual acuity (letters) from baseline

- We performed a network meta-analysis (NMA) based on change data measured by letters
- Transformations were performed as needed:
  - logMAR = log (1/decimals) = (85-letters)/50
  - ${Mean}_{change}={Mean}_{final}-{Mean}_{baseline}$
  - ${SD}_{change}=\sqrt{{SD}_{baseline}^{2}+{SD}_{final}^{2}-(2\times Corr\times{SD}_{baseline}\times{SD}_{final}}$ (Corr was estimated as 0.5)
- Clinical similarity in patient characteristics across trials were carefully assessed before analysis, and patients in one randomized controlled trial (Baker 2019) were considered to not be similar to other patients:
  - The mean visual acuity (VA) of participants in Baker 2019 was 85 letters, and the mean VA of other included patients was between 43 and 73.5 letters
  - Baker 2019 was not included in the NMA due to clinical heterogeneity

## Mean change in central retinal thickness (μm) from baseline

- We performed a NMA based on change data
- Transformations were performed as needed:
  - ${Mean}_{change}={Mean}_{final}-{Mean}_{baseline}$
  - ${SD}_{change}=\sqrt{{SD}_{baseline}^{2}+{SD}_{final}^{2}-(2\times Corr\times{SD}_{baseline}\times{SD}_{final}}$ (Corr was estimated as 0.5)

# Appendix 5 Results of network meta-analyses (NMAs) for secondary outcomes

## The Proportion of patients with a gain of at least 10 Early Treatment Diabetic Retinopathy Study (ETDRS) letters (2 ETDRS lines or 0.2 logMAR)

### Network geometry

All population populations at 1-year follow-up (a. 12 trials) and 2-year follow-up (b. 5 trials). Population with worse baseline VA at 1-year follow-up (c. 8 trials) and 2-year follow-up (d. 5 trials). Direct comparisons are represented by the black lines connecting the different interventions. Line width is proportional to the number of trials including every pair of interventions. CON, conbercept; Dex, dexamethasone implant; IVA, aflibercept; IVB, bevacizumab; IVR, ranibizumab; Laser, laser; Sham, placebo.

**
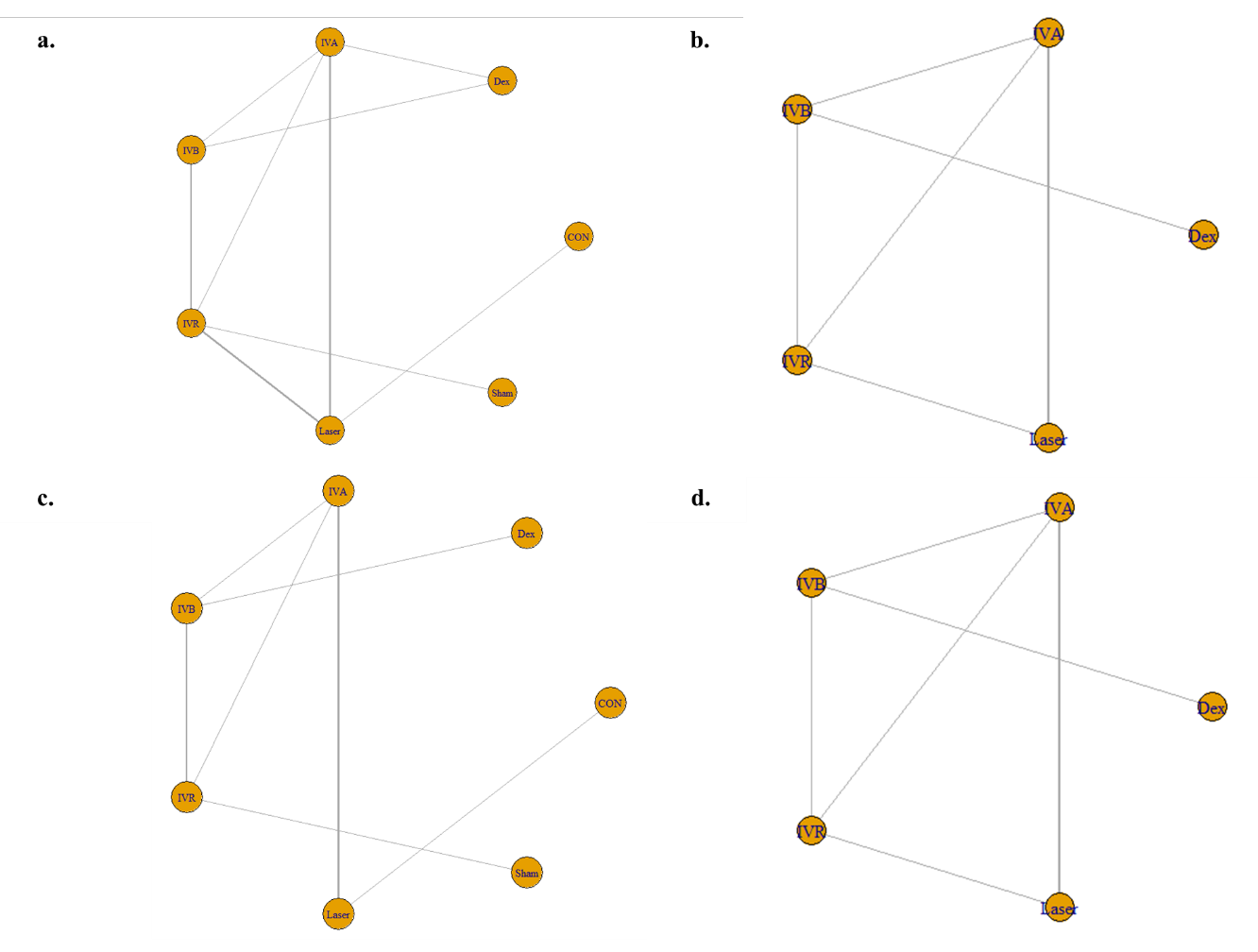
**

### NMA results

| ***At 1-year follow-up*** | | | | | | | | | | |
| --- | --- | --- | --- | --- | --- | --- | --- | --- | --- | --- |
| ***Aflibercept*** | *1.57 (0.85, 2.92)* | | ***2.26 (1.17, 4.33)*** | ***2.29 (1.26, 4.24)*** | | *2.22 (0.77, 6.35)* | | ***5.34 (4.08, 7.04)*** | | ***11.23 (4.13, 33.06)*** |
| *1.36 (0.83, 2.27)* | ***Ranibizumab*** | | *1.44 (0.59, 3.53)* | *1.46 (0.88, 2.45)* | | *1.41 (0.51, 3.85)* | | ***3.42 (1.74, 6.66)*** | | ***7.15 (3.23, 17.25)*** |
| *2.15 (0.83, 5.78)* | *1.59 (0.59, 4.25)* | | ***Conbercept*** | *1.01 (0.42, 2.49)* | | *0.98 (0.29, 3.4)* | | ***2.36 (1.32, 4.36)*** | | ***5.01 (1.48, 17.51)*** |
| *1.74 (0.97, 3.24)* | *1.29 (0.72, 2.33)* | | *0.81 (0.27, 2.41)* | ***Bevacizumab*** | | *0.97 (0.41, 2.27)* | | ***2.33 (1.2, 4.51)*** | | ***4.9 (1.9, 13.56)*** |
| *1.92 (0.87, 4.36)* | *1.42 (0.59, 3.4)* | | *0.89 (0.26, 3.07)* | *1.1 (0.49, 2.46)* | | ***Dexamethasone implant*** | | *2.41 (0.81, 7.18)* | | ***5.08 (1.39, 19.13)*** |
| ***5.13 (3.42, 7.81)*** | ***3.78 (2.4, 5.96)*** | | *2.37 (0.97, 5.74)* | ***2.94 (1.53, 5.51)*** | | ***2.66 (1.11, 6.36)*** | | ***Laser*** | | *2.1 (0.74, 6.44)* |
| ***9.81 (3.14, 32.94)*** | ***7.19 (2.6, 21.77)*** | | ***4.52 (1.08, 20.15)*** | ***5.6 (1.73, 19.47)*** | | ***5.1 (1.29, 21.03)*** | | *1.9 (0.62, 6.34)* | | ***Placebo*** |
| ***At 2-year follow-up*** | | | | | | | | | | |
| ***Aflibercept*** | | *1.2 (0.68, 2.15)* | | | *1.55 (0.84, 2.89)* | | *1.29 (0.45, 3.67)* | | ***3.89 (2.89, 5.3)*** | |
| *1.15 (0.78, 1.7)* | | ***Ranibizumab*** | | | *1.28 (2.38, 0.7)* | | *1.06 (0.38, 3.03)* | | ***3.23 (1.74, 6)*** | |
| *1.43 (0.95, 2.12)* | | *1.23 (0.82, 1.85)* | | | ***Bevacizumab*** | | *0.83 (0.35, 1.96)* | | ***2.51 (1.29, 4.96)*** | |
| *1.18 (0.46, 3.04)* | | *1.02 (0.39, 2.63)* | | | *0.83 (0.35, 1.92)* | | ***Dexamethasone implant*** | | ***3.02 (1.02, 9.06)*** | |
| ***3.88 (2.88, 5.27)*** | | ***3.37 (2.11, 5.39)*** | | | ***2.72 (1.67, 4.46)*** | | ***3.3 (1.23, 8.88)*** | | ***Laser*** | |

Results of network meta-analysis for all the population were listed in the lower triangle, and the estimation was calculated as the column-defining treatment compared with the row-defining treatment. ORs higher than 1 favor the column-defining treatment. Results of network meta-analysis for the population with worse baseline VA were listed in the upper triangle, and the estimation was calculated as the row-defining treatment compared with the column-defining treatment. ORs higher than 1 favor the row-defining treatment. Statistically significant was presented as bold italic format. To obtain ORs for comparisons in the opposite direction, reciprocals should be taken. OR, odds ratio.

### Ranking with SUCRA

| **At 1-year follow-up** | | | | |
| --- | --- | --- | --- | --- |
| **Interventions** | **All population** | | **Population with worse baseline VA** | |
|  | **Ranks** | SUCRA | **Ranks** | SUCRA |
| *Aflibercept* | 1 | 0.96293750 | 1 | 0.97405833 |
| *Ranibizumab* | 2 | 0.76037500 | 2 | 0.75756250 |
| *Conbercept* | 5 | 0.48653333 | 5 | 0.53590833 |
| *Bevacizumab* | 3 | 0.57580833 | 4 | 0.50376250 |
| *Dexamethasone implant* | 4 | 0.53344167 | 3 | 0.54916667 |
| *Laser* | 6 | 0.15444583 | 6 | 0.16363750 |
| *Placebo* | 7 | 0.02645833 | 7 | 0.01590417 |
| **At 2-year follow-up** | | | | |
| **Interventions** | **All population** | | **Population with worse baseline VA** | |
|  | **Ranks** | SUCRA | **Ranks** | SUCRA |
| *Aflibercept* | 1 | 0.83946875 | 1 | 0.8350438 |
| *Ranibizumab* | 2 | 0.65051875 | 2 | 0.6495563 |
| *Bevacizumab* | 4 | 0.38016875 | 4 | 0.4055437 |
| *Dexamethasone implant* | 3 | 0.62776250 | 3 | 0.4055437 |
| *Laser* | 5 | 0.00208125 | 5 | 0.0068375 |

SUCRA, surface under the cumulative ranking curve.

## Mean change in central retinal thickness (μm) from baseline

### Network geometry

All population populations at 1-year follow-up (a. 17 trials) and 2-year follow-up (b. 4 trials). Population with worse baseline VA at 1-year follow-up (c. 10 trials). Direct comparisons are represented by the black lines connecting the different interventions. Line width is proportional to the number of trials including every pair of interventions. CON, conbercept; Dex, dexamethasone implant; IVA, aflibercept; IVB, bevacizumab; IVR, ranibizumab; Laser, laser; Sham, placebo.


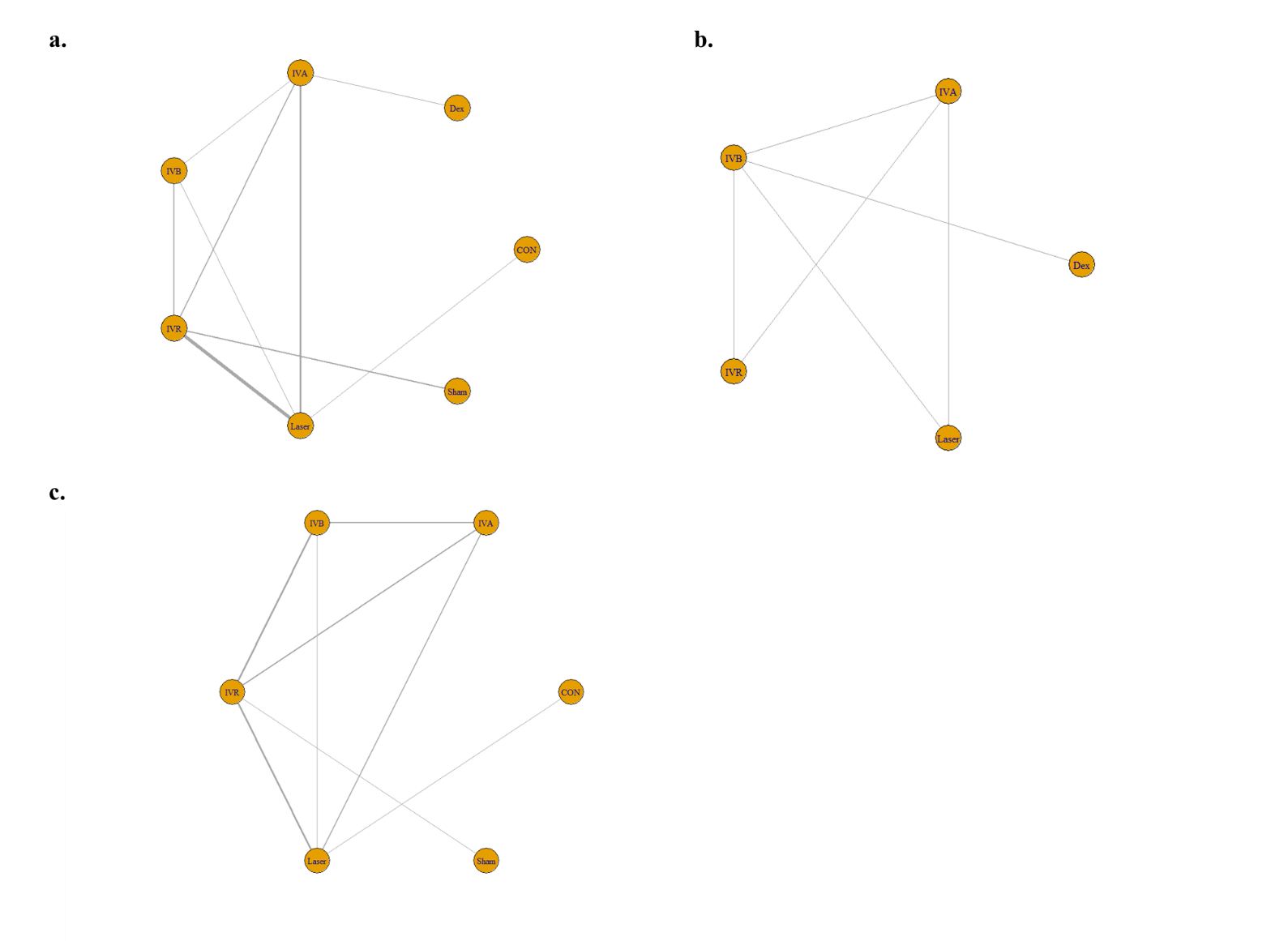


### NMA results

| **At 1-year follow-up** | | | | | | | | | |
| --- | --- | --- | --- | --- | --- | --- | --- | --- | --- |
| **Aflibercept** | ***-60.72 (-91.58, -24.69)*** | | -33.76 (-110.94, 44.79) | | ***-80.39 (-116.08, -41.67)*** | – | ***-103.7 (-137.02, -72.33)*** | | ***-206.38 (-283.14, -125.44)*** |
| -32.29 (-70.45, 5.33) | **Ranibizumab** | | 27 (-50.98, 100.46) | | -19.48 (-51.99, 9.88) | – | ***-42.89 (-73.96, -19.34)*** | | ***-145.81 (-217.92, -74.46)*** |
| -8.73 (-103.38, 84.54) | 23.58 (-68.43, 114.15) | | **Conbercept** | | -46.67 (-124.96, 33.07) | - | ***-70.25 (-141.45, -0.35)*** | | ***-172.69 (-275.19, -66.99)*** |
| ***-56.02 (-105.43, -5.9)*** | -23.61 (-66.84, 20.37) | | -47.26 (-145.8, 50.78) | | **Bevacizumab** | – | –8.04 (–19.7, 3.56) | | ***–128.82 (–179.62, –77.63)*** |
| ***108.97 (25.91, 191.53)*** | ***141.22 (50.07, 232.55)*** | | 117.45 (-6.07, 243.47) | | ***164.64 (68.07, 262.4)*** | **Dexamethasone implant** | – | | ***–*** |
| ***-78.47 (-114.87, -44.28)*** | ***-46.2 (-75.15, -19.03)*** | | -69.88 (-157.06, 16.97) | | -22.63 (-69.55, 21.95) | ***-187.66 (-277.7, -98.23)*** | **Laser** | | ***–120.86 (–170.84, –70.13)*** |
| ***-161.12 (-245.92, -70.52)*** | ***-128.56 (-205.71, -46.48)*** | | -152.41 (-269.49, -28.1) | | ***-104.87 (-193.23, -11.43)*** | ***-269.69 (-386.81, -147.03)*** | -82.31 (-163.84, 5.56) | | **Placebo** |
| **At 2-year follow-up** | | | | | | | | | |
| **Aflibercept** | | – | | – | | – | | ***–*** | |
| –14.74 (–41.55, 12.4) | | **Ranibizumab** | | – | | – | | ***–*** | |
| ***–29.47 (–54.03, –4.98)*** | | –14.73 (–42.4, 12.83) | | **Bevacizumab** | | – | | ***–*** | |
| –26.41 (–83.2, 30.87) | | –11.6 (–70.36, 47.41) | | 3.36 (–48.61, 54.59) | | **Dexamethasone implant** | | ***–*** | |
| –10.48 (–23.61, 2.63) | | 4.36 (–25.31, 33.25) | | 18.98 (–7.07, 45.14) | | 15.82 (–42.07, 73.66) | | **Laser** | |

Results of network meta-analysis for all population were listed in the lower triangle, and the estimation was calculated as the column-defining treatment compared with the row-defining treatment. MDs higher than 0 favor the column-defining treatment. Results of network meta-analysis for population with worse baseline VA were listed in the upper triangle, and the estimation was calculated as the row-defining treatment compared with the column-defining treatment. MDs higher than 0 favor the row-defining treatment. Statistically significant was presented as bold italic format. To obtain MDs for comparisons in the opposite direction, negatives should be taken.MD, mean difference.

### Ranking with SUCRA

| **At 1-year follow-up** | | | | |
| --- | --- | --- | --- | --- |
| **Interventions** | **All population** | | **Population with worse baseline VA** | |
|  | **Ranks** | SUCRA | **Ranks** | SUCRA |
| *Aflibercept* | 2 | 0.75373333 | 1 | 0.962430 |
| *Ranibizumab* | 4 | 0.53460833 | 3 | 0.630770 |
| *Bevacizumab* | 5 | 0.35850000 | 4 | 0.424955 |
| *Conbercept* | 3 | 0.65497083 | 2 | 0.761695 |
| *Dexamethasone implant* | 1 | 0.99306667 | – | – |
| *Laser* | 6 | 0.19496250 | 5 | 0.217485 |
| *Placebo* | 7 | 0.01015833 | 6 | 0.002665 |
| **At 2-year follow-up** | | | | |
| **Interventions** | **All population** | | **Population with worse baseline VA** | |
|  | **Ranks** | SUCRA | **Ranks** | SUCRA |
| *Aflibercept* | 1 | 0.9013313 | – | – |
| *Ranibizumab* | 3 | 0.5095312 | – | – |
| *Bevacizumab* | 5 | 0.1705875 | – | – |
| *Dexamethasone implant* | 4 | 0.3444312 | – | – |

SUCRA, surface under the cumulative ranking curve.

## Adverse events

### Network geometry

Serious adverse events in all population at 1-year follow-up (a. 8 trials). Ocular adverse events in all population at 1-year follow-up (b. 9 trials). Direct comparisons are represented by the black lines connecting the different interventions. Line width is proportional to the number of trials including every pair of interventions. CON, conbercept; Dex, dexamethasone implant; IVA, aflibercept; IVB, bevacizumab; IVR, ranibizumab; Laser, laser; Sham, placebo.


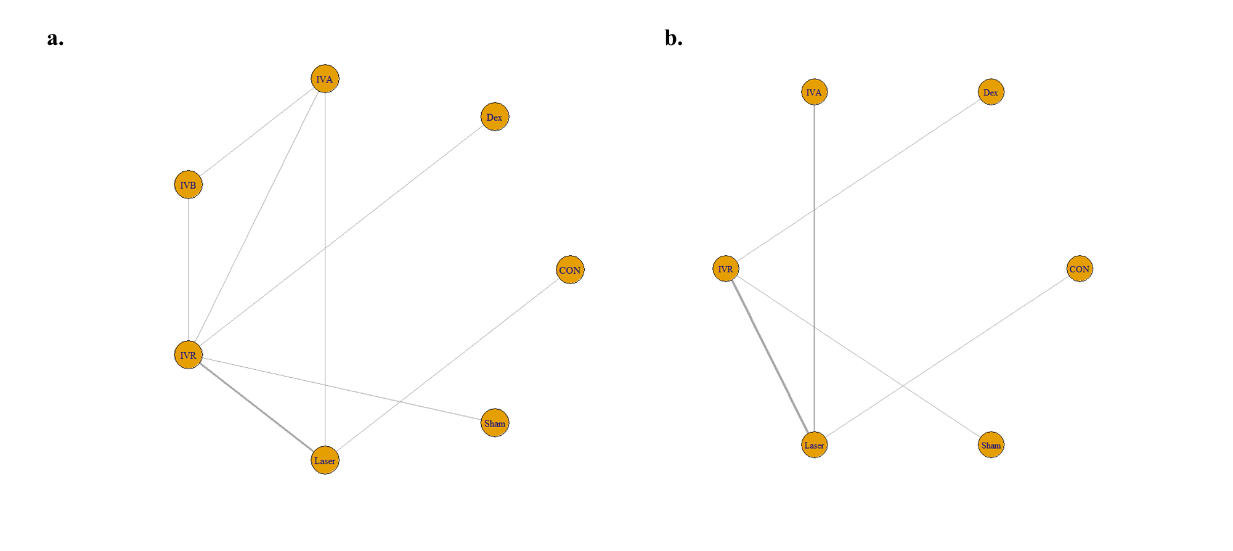


### NMA results

| **At 1-year follow-up** | | | | | | |
| --- | --- | --- | --- | --- | --- | --- |
| **Aflibercept** | ***0.45 (0.28, 0.7)*** | ***0.36 (0.21, 0.63)*** | ***–*** | ***0.04 (0.02, 0.08)*** | ***0.75 (0.59, 0.96)*** | 1.22 (0.51, 2.96) |
| 0.93 (0.66, 1.33) | **Ranibizumab** | 0.81 (0.43, 1.53) | – | ***0.08 (0.04, 0.16)*** | ***1.7 (1.16, 2.52)*** | ***2.75 (1.31, 5.82)*** |
| 1.11 (0.52, 2.39) | 1.18 (0.57, 2.51) | **Conbercept** | - | ***0.1 (0.04, 0.25)*** | ***2.09 (1.27, 3.46)*** | ***3.38 (1.27, 9.06)*** |
| 0.66 (0.46, 0.96) | 0.71 (0.49, 1.02) | 0.6 (0.27, 1.33) | **Bevacizumab** | – | ***–*** | ***–*** |
| 0.91 (0.15, 5.37) | 0.98 (0.17, 5.57) | 0.82 (0.12, 5.44) | 1.38 (0.23, 8.26) | **Dexamethasone implant** | ***20.59 (9.68, 47.2)*** | ***33.55 (12.6, 93.38)*** |
| 0.98 (0.66, 1.49) | 1.05 (0.73, 1.52) | 0.89 (0.46, 1.69) | 1.49 (0.92, 2.4) | 1.08 (0.18, 6.47) | **Laser** | 1.62 (0.69, 3.78) |
| 0.91 (0.37, 2.38) | 0.97 (0.42, 2.4) | 0.82 (0.27, 2.6) | 1.37 (0.55, 3.64) | 0.99 (0.15, 7.14) | 0.92 (0.38, 2.44) | **Placebo** |

Results of network meta-analysis for all population were listed in the lower triangle, and the estimation was calculated as the column-defining treatment compared with the row-defining treatment. ORs higher than 1 favor the column-defining treatment. Results of network meta-analysis for population with worse baseline VA were listed in the upper triangle, and the estimation was calculated as the row-defining treatment compared with the column-defining treatment. ORs higher than 1 favor the row-defining treatment. Statistically significant was presented as bold italic format. To obtain ORs for comparisons in the opposite direction, reciprocals should be taken. OR, odds ratio.

### Ranking with SUCRA

| **At 1-year follow-up** | | | | |
| --- | --- | --- | --- | --- |
| **Interventions** | **Serious adverse events** | | **Ocular adverse events** | |
|  | **Ranks** | SUCRA | **Ranks** | SUCRA |
| *Aflibercept* | 2 | 0.6148833 | 2 | 0.862625 |
| *Ranibizumab* | 4 | 0.5114292 | 4 | 0.349620 |
| *Conbercept* | 1 | 0.6708708 | 5 | 0.253770 |
| *Bevacizumab* | 7 | 0.1339250 | - | – |
| *Dexamethasone implant* | 5 | 0.549545 | 6 | 0.0000000 |
| *Laser* | 3 | 0.5822792 | 3 | 0.627170 |
| *Placebo* | 6 | 0.4901000 | 1 | 0.906815 |

SUCRA, surface under the cumulative ranking curve.

# Appendix 6 Overall heterogeneity, consistency, and forest plots for each comparison

CON, conbercept; Dex, dexamethasone implant; IVA, aflibercept; IVB, bevacizumab; IVR, ranibizumab; Laser, laser; Sham, placebo.

## Mean change in BCVA measured by ETDRS letters from baseline

### Overall heterogeneity and consistency

|  | **Overall *I^2^*** | **DIC** | Differences of DIC [should be less than 5] |
| --- | --- | --- | --- |
| **All population at 1-year follow-up** | | | |
| Fixed-effect model Consistency model | 25% | 71.99546 | 0.8624 |
| Fixed-effect model Non-consistency model | 17% | 71.13306 | – |
| **All population at 2-year follow-up** | | | |
| Fixed-effect model Consistency model | 1% | 19.109006 | 1.017727 |
| Fixed-effect model Non-consistency model | 1% | 20.126733 | – |
| **Population with worse baseline VA at 1-year follow-up** | | | |
| Fixed-effect model Consistency model | 10% | 44.87026 | 3.11727 |
| Fixed-effect model Non-consistency model | 11% | 47.98753 | – |
| **Population with worse baseline VA at 2-year follow-up** | | | |
| Fixed-effect model Consistency model | 0% | 18.391804 | 1.684222 |
| Fixed-effect model Non-consistency model | 1% | 20.076026 | – |

Note: Fixed-effect consistency model was performed for final analysis.

### Forest plots with heterogeneity test

- 1. **All population at 1-year follow-up**


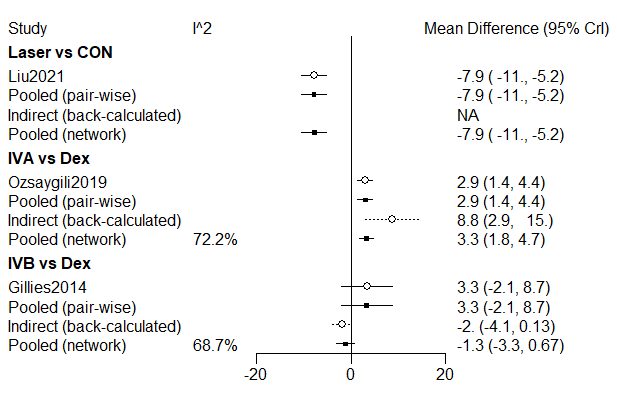

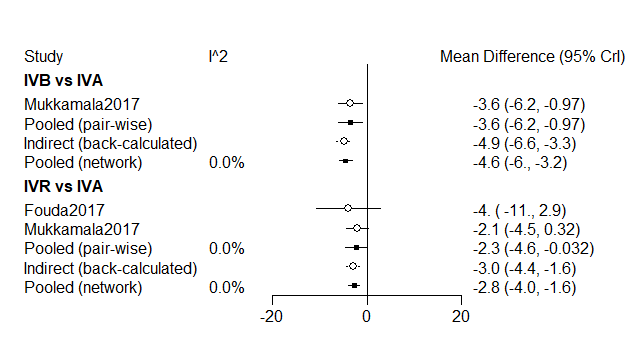


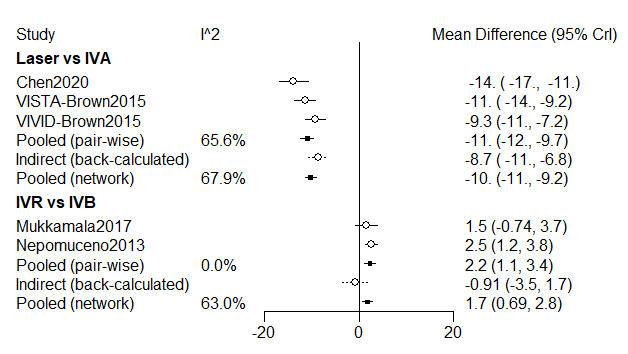

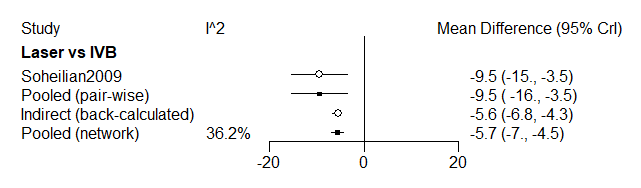


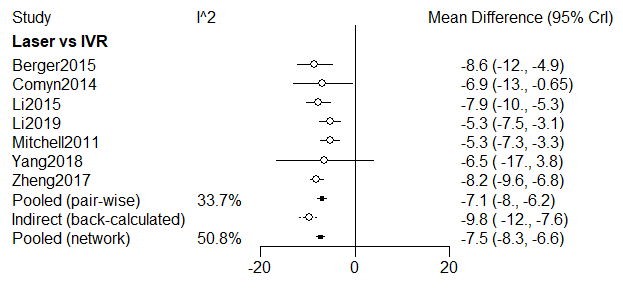

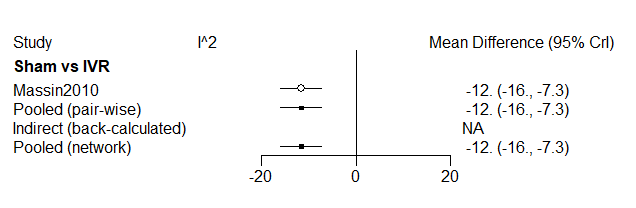


- 1. **All population at 2-year follow-up**


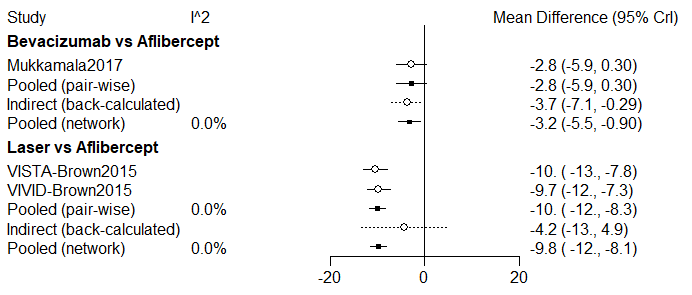

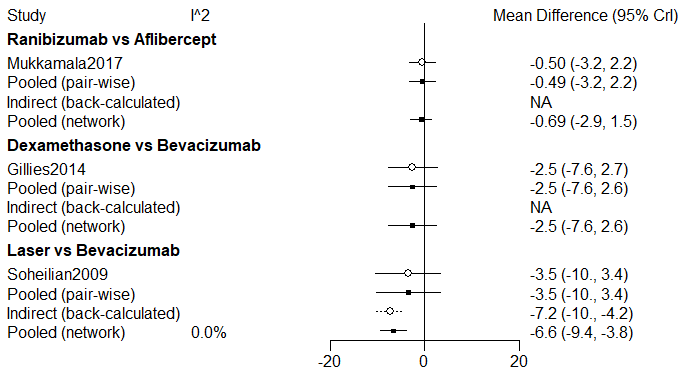


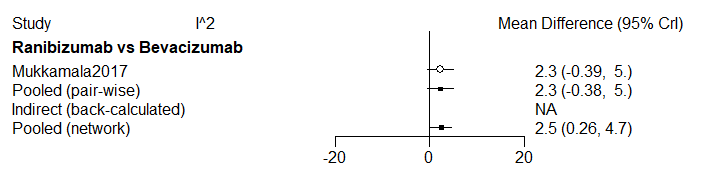


- 1. **Population with worse baseline VA at 1-year follow-up**


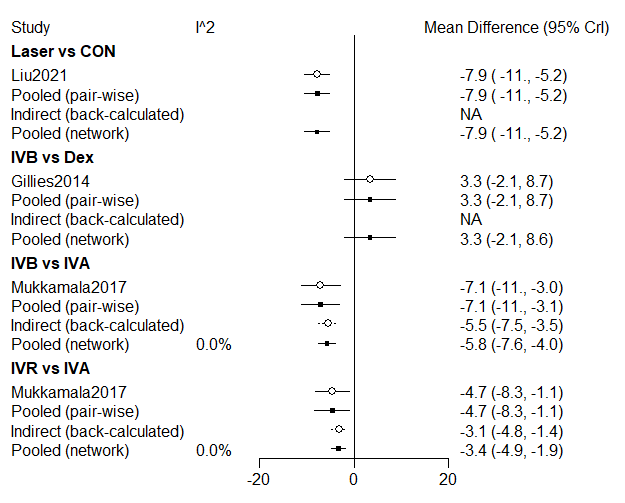

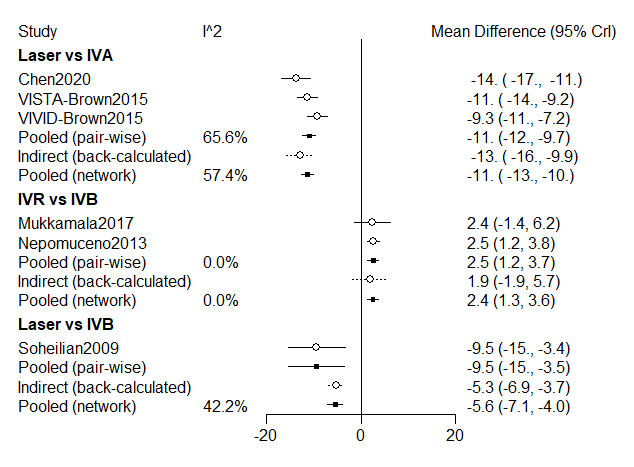

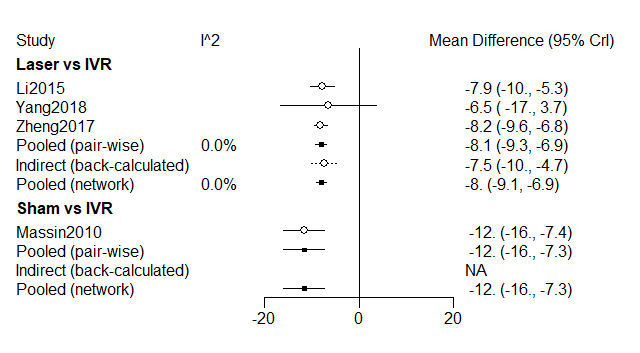


- 1. **Population with worse baseline VA at 2-year follow-up**


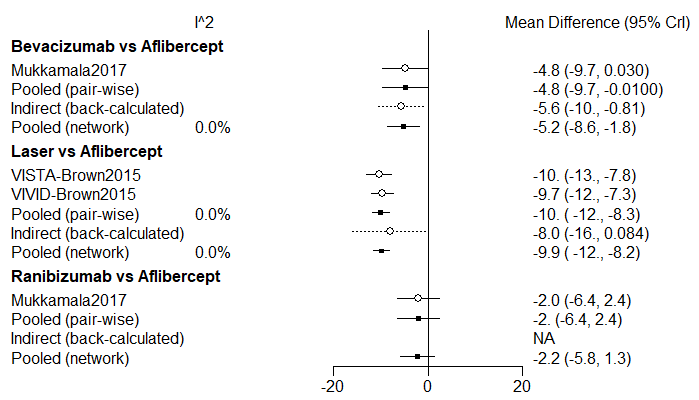

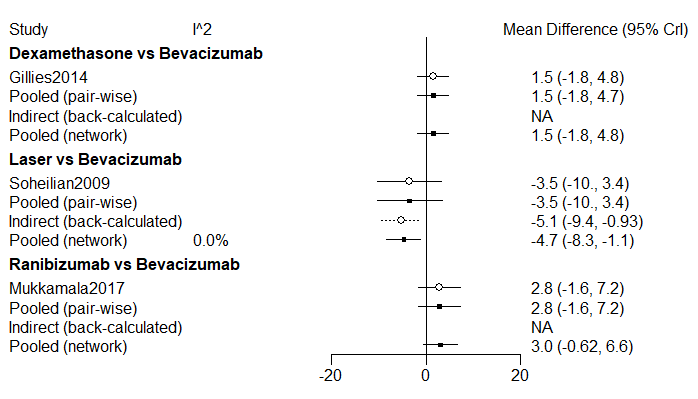


## The proportion of patients with a gain of at least 15 Early Treatment Diabetic Retinopathy Study (ETDRS) letters (3 ETDRS lines or 0.3 logMAR

### Overall heterogeneity and consistency

|  | **Overall *I^2^*** | **DIC** | Differences of DIC [should be less than 5] |
| --- | --- | --- | --- |
| **All population at 1-year follow-up** | | | |
| Fixed-effect model Consistency model | 0% | 35.66299 | 3.8232 |
| Fixed-effect model Non-consistency model | 0% | 39.48619 | – |
| **All population at 2-year follow-up** | | | |
| Fixed-effect model Consistency model | 0% | 23.07218 | 4.08108 |
| Fixed-effect model Non-consistency model | 0% | 27.15326 | – |
| **Population with worse baseline VA at 1-year follow-up** | | | |
| Fixed-effect model Consistency model | 0% | 28.9814 | 2.11698 |
| Fixed-effect model Non-consistency model | 0% | 31.09838 | – |
| **Population with worse baseline VA at 2-year follow-up** | | | |
| Fixed-effect model Consistency model | 0% | 23.04160 | 4.15913 |
| Fixed-effect model Non-consistency model | 0.1% | 27.20073 | – |

Note: Fixed-effect consistency model was performed for final analysis.

### Forest plots with heterogeneity test

- 1. **All population at 1-year follow-up**


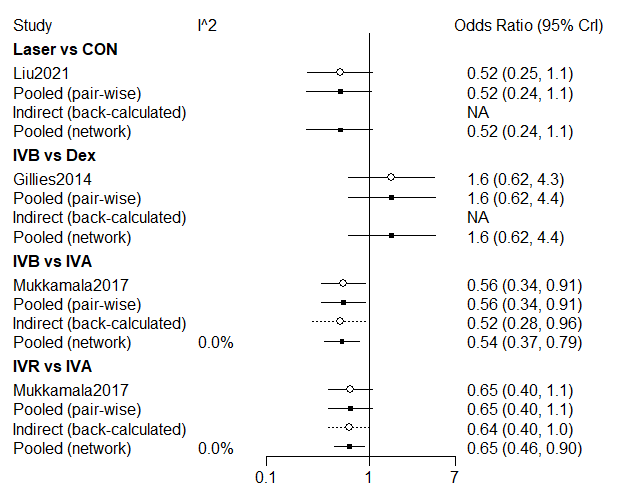

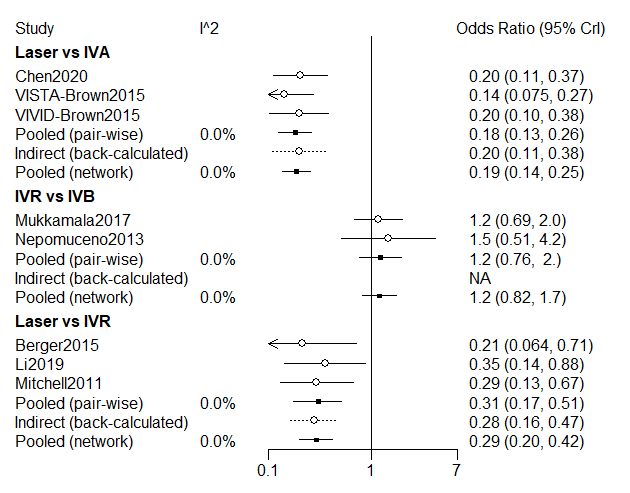

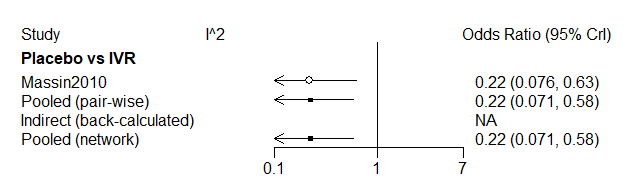


- 1. **All population at 2-year follow-up**


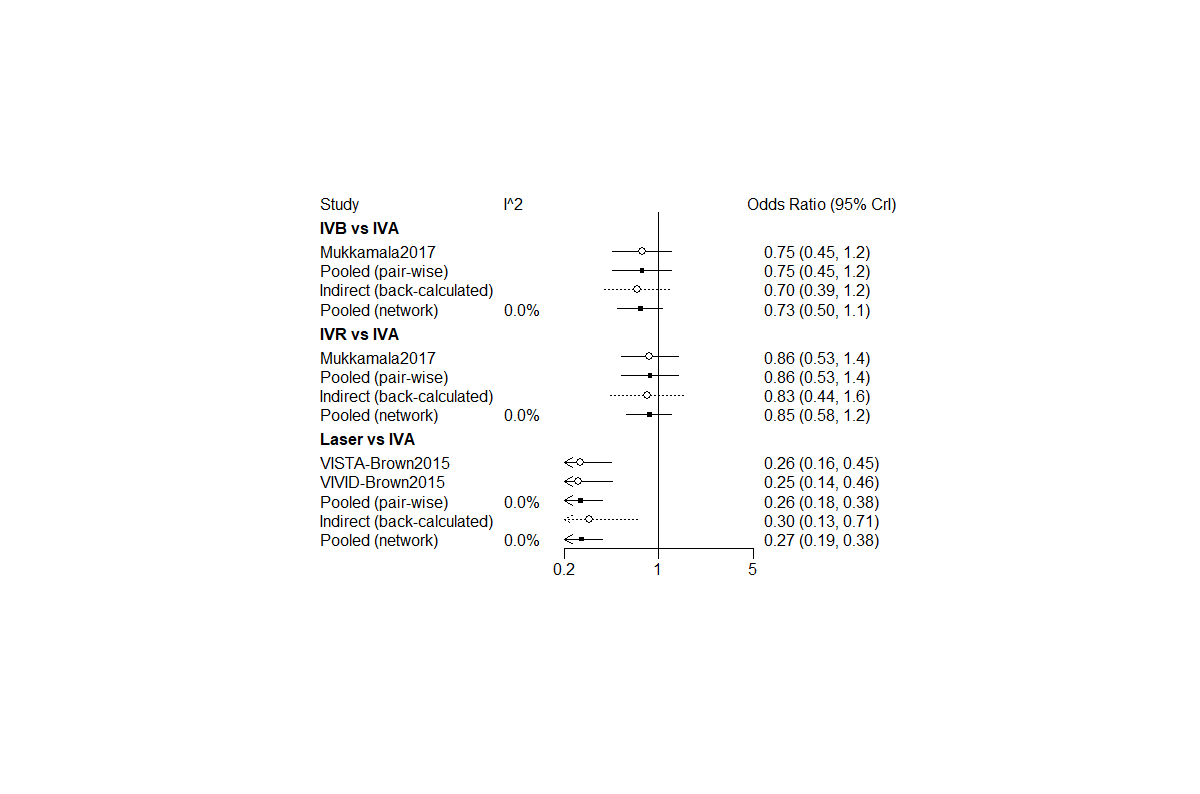

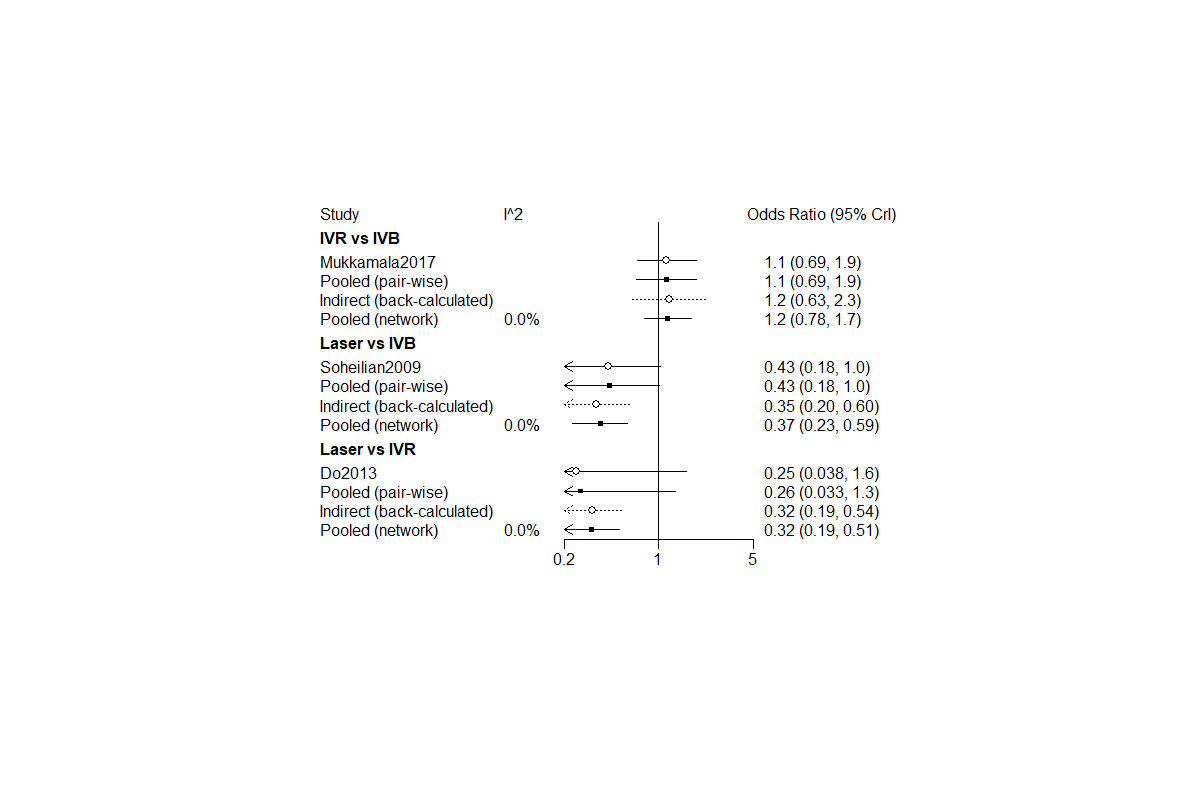

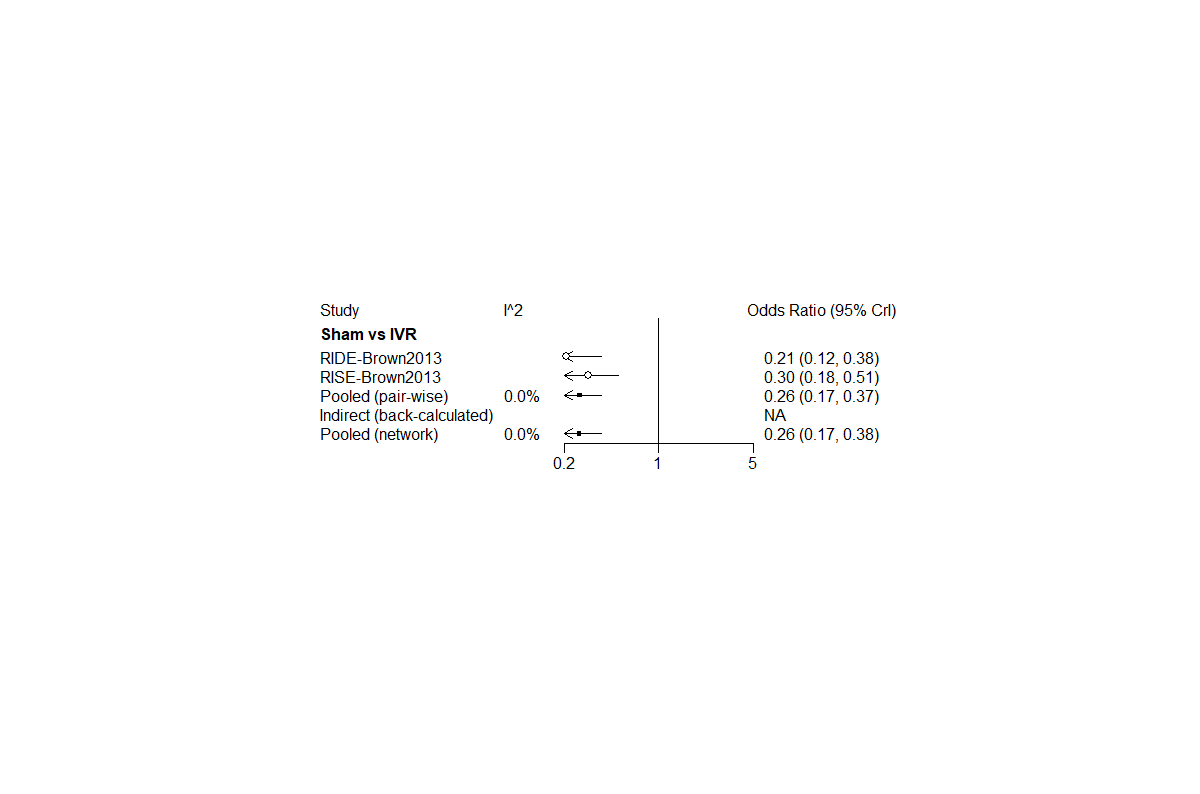


- 1. **Population with worse baseline VA at 1-year follow-up**


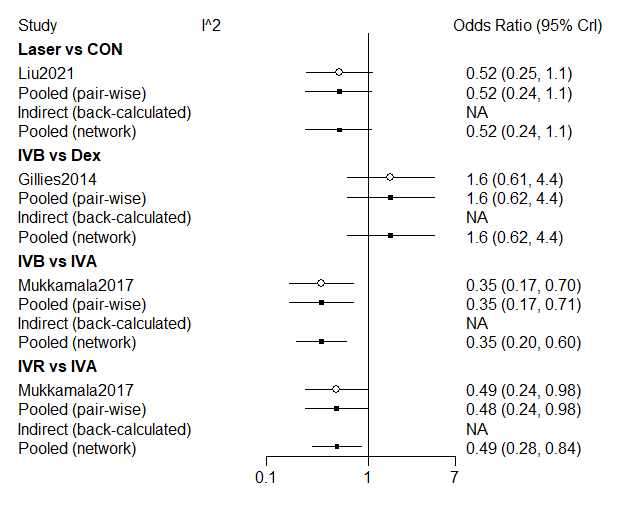

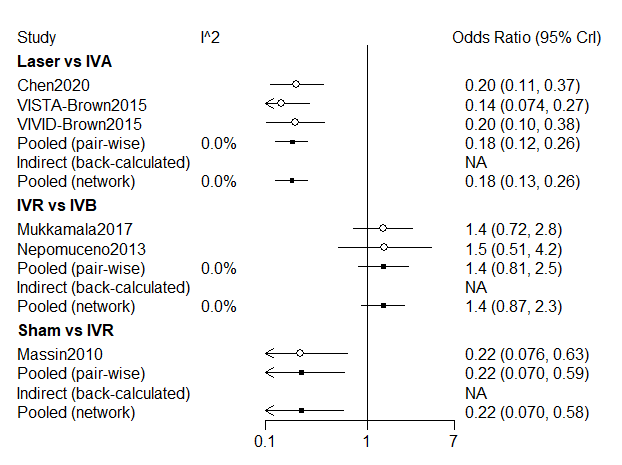


- 1. **Population with worse baseline VA at 2-year follow-up**


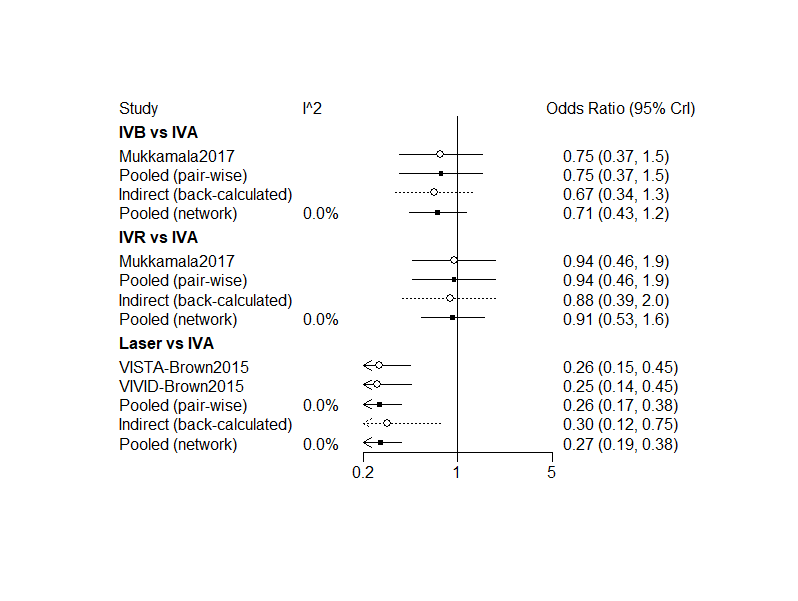

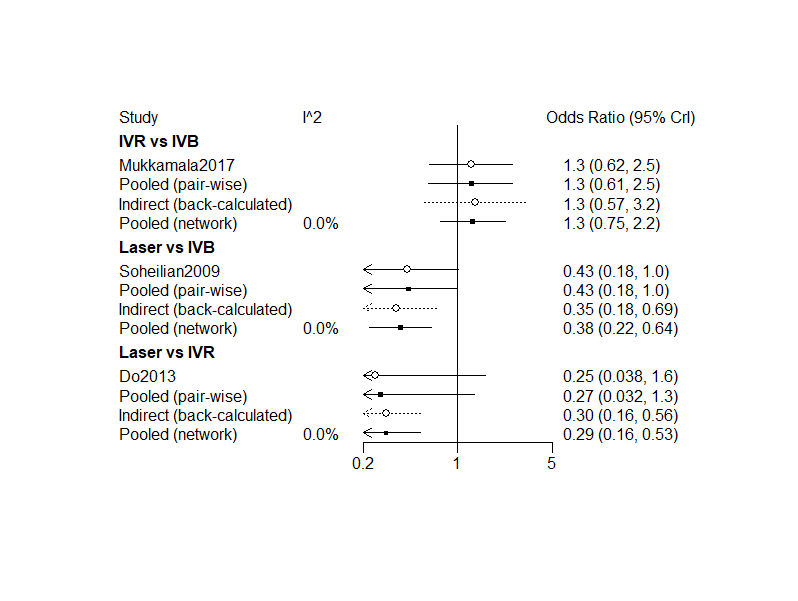

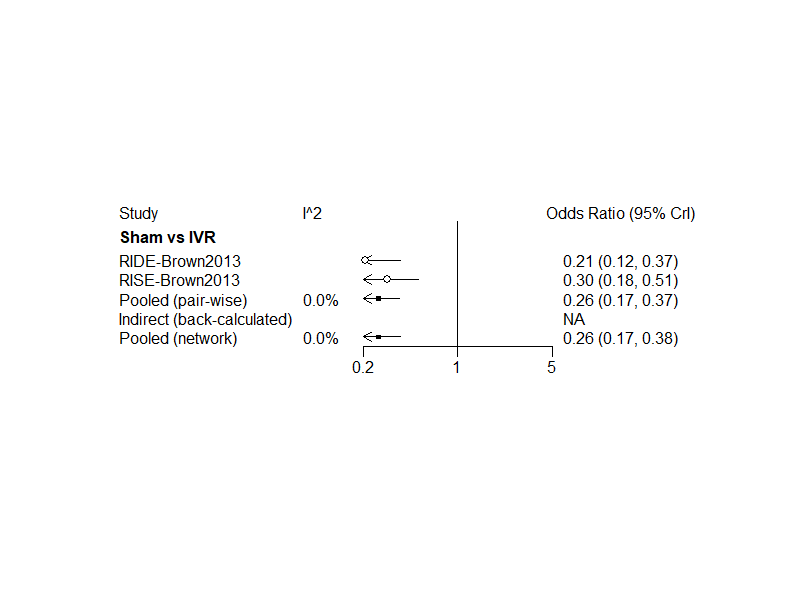


## The proportion of patients with a gain of at least 10 Early Treatment Diabetic Retinopathy Study (ETDRS) letters (2 ETDRS lines or 0.2 logMAR

### Overall heterogeneity and consistency

|  | **Overall *I^2^*** | **DIC** | Differences of DIC [should be less than 5] |
| --- | --- | --- | --- |
| **All population at 1-year follow-up** - Random-effects consistency model was performed for final analysis. | | | |
| Random-effects model Consistency model | 2% | 45.98059 | 3.83994 |
| Random-effects model Non-consistency model | 6% | 49.82053 | – |
| **All population at 2-year follow-up  -** Fixed-effect consistency model was performed for final analysis. | | | |
| Fixed-effect model Consistency model | 0% | 18.360985 | 2.097795 |
| Fixed-effect model Non-consistency model | 4% | 20.45878 | – |
| **Population with worse baseline VA at 1-year follow-up** **-** Fixed-effect consistency model was performed for final analysis. | | | |
| Fixed-effect model Consistency model | 18% | 33.71066 | 2.03758 |
| Fixed-effect model Non-consistency model | 22% | 35.74824 | – |
| **Population with worse baseline VA at 2-year follow-up -** Fixed-effect consistency model was performed for final analysis. | | | |
| Fixed-effect model Consistency model | 0% | 18.411414 | 2.114656 |
| Fixed-effect model Non-consistency model | 4% | 20.52607 | – |

### Forest plots with heterogeneity test

- 1. **All population at 1-year follow-up**


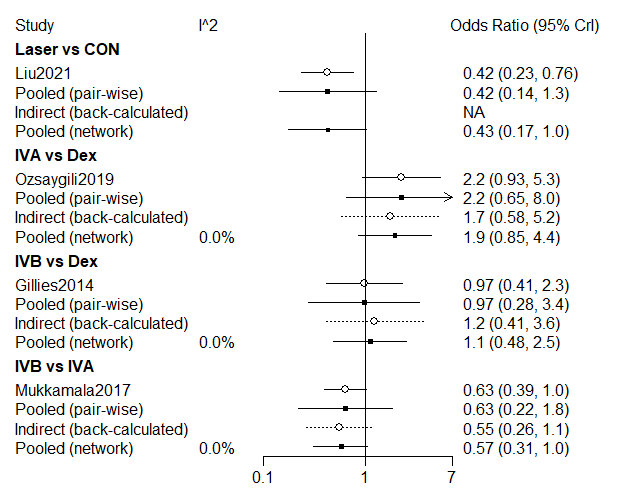

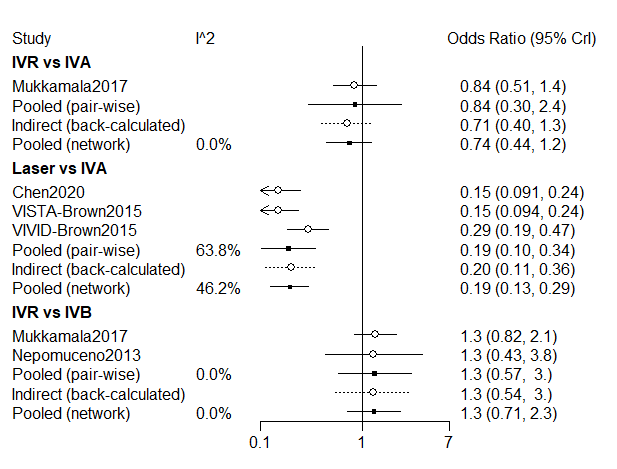

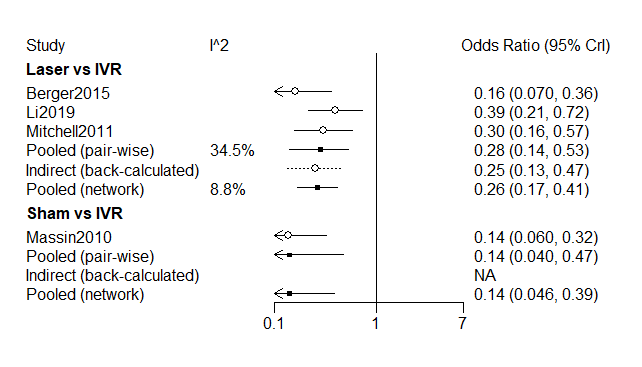


- 1. **All population at 2-year follow-up**


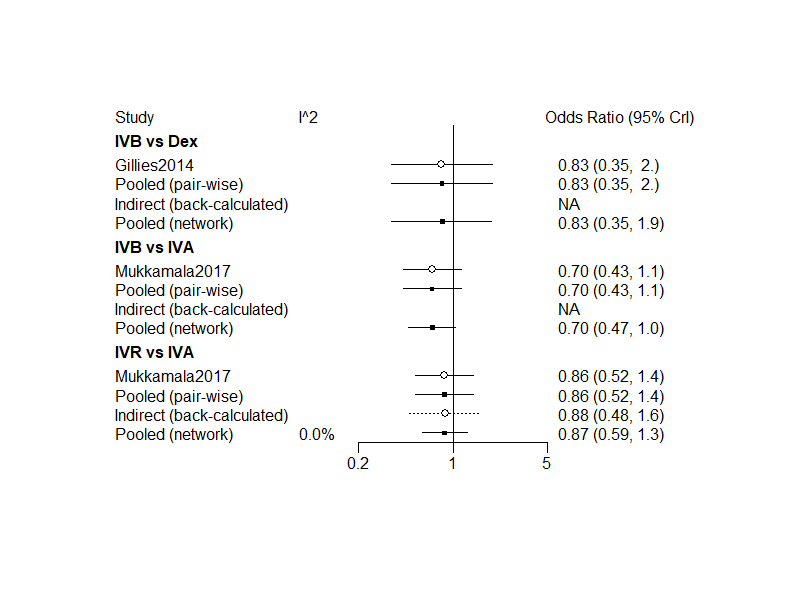

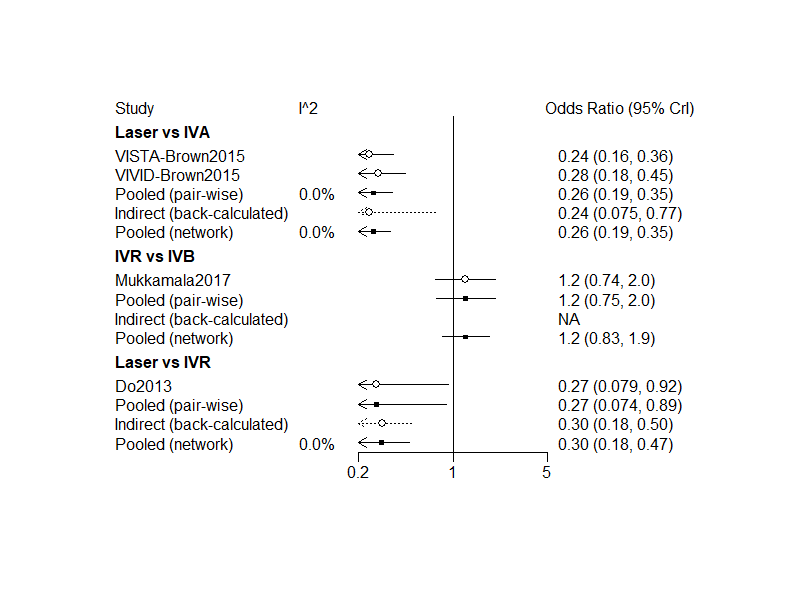


- 1. **Population with worse baseline VA at 1-year follow-up**


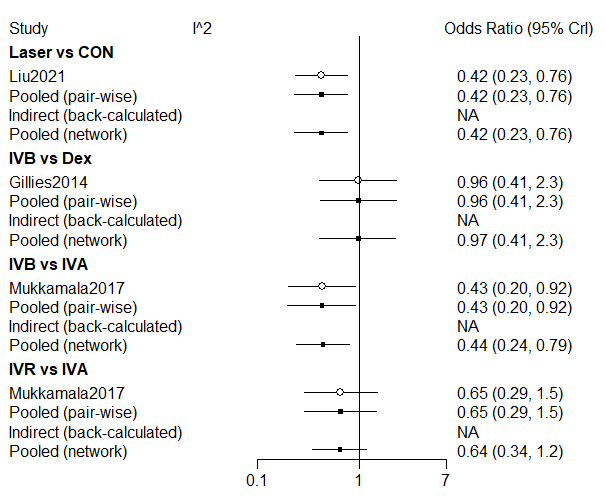

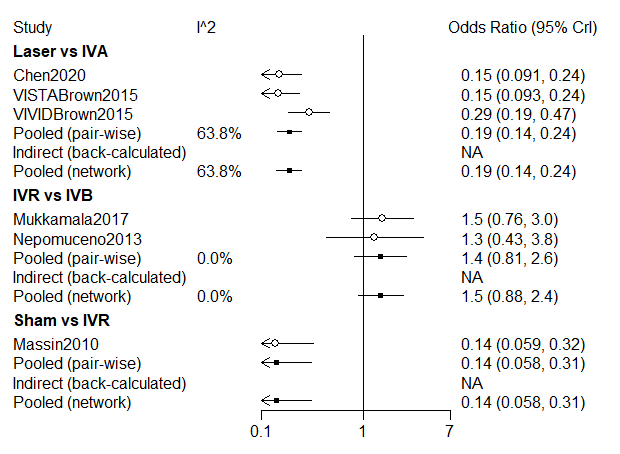


- 1. **Population with worse baseline VA at 2-year follow-up**


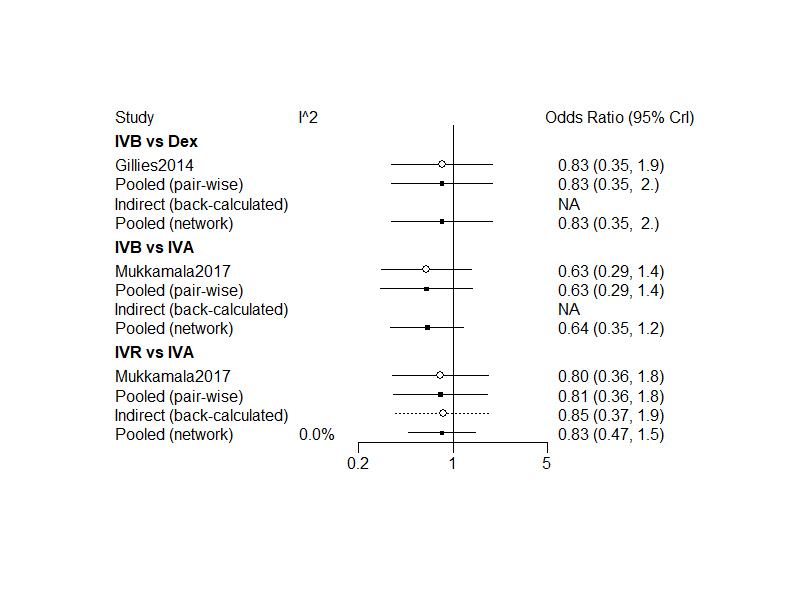

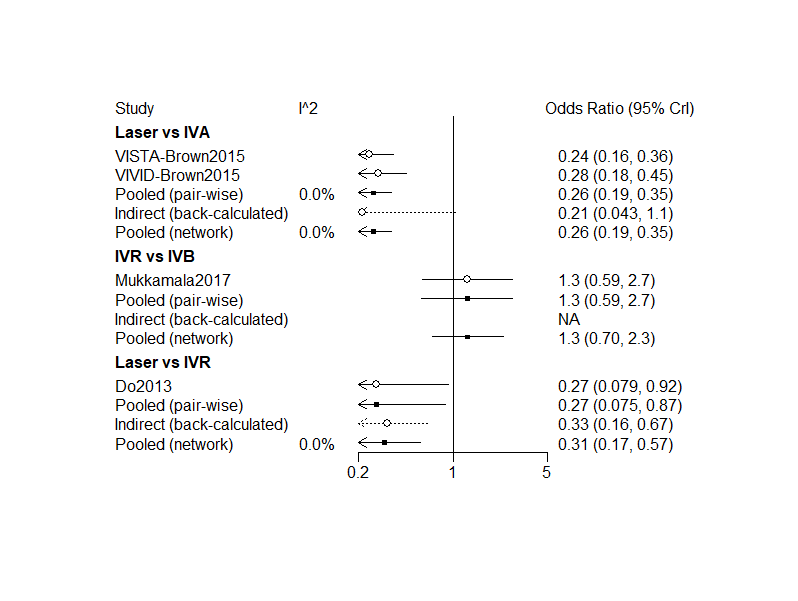


## Mean change in central retinal thickness (μm) from baseline

### Overall heterogeneity and consistency

|  | **Overall *I^2^*** | **DIC** | Differences of DIC [should be less than 5] |
| --- | --- | --- | --- |
| **All population at 1-year follow-up** - Random-effects consistency model was performed for final analysis. | | | |
| Random-effects model Consistency model | 0.9% | 66.63185 | 0.9216 |
| Random-effects model Non-consistency model | 1% | 67.55345 | – |
| **All population at 2-year follow-up  -** Fixed-effect consistency model was performed for final analysis. | | | |
| Fixed-effect model Consistency model | 37% | 20.720237 | 2.654953 |
| Fixed-effect model Non-consistency model | 11% | 18.065284 | – |
| **Population with worse baseline VA at 1-year follow-up -** Random-effects consistency model was performed for final analysis. | | | |
| Random-effects model Consistency model | 3% | 45.52968 | 0.93032 |
| Random-effects model Non-consistency model | 5% | 46.46000 | – |

### Forest plots with heterogeneity test

- 1. **All population at 1-year follow-up**


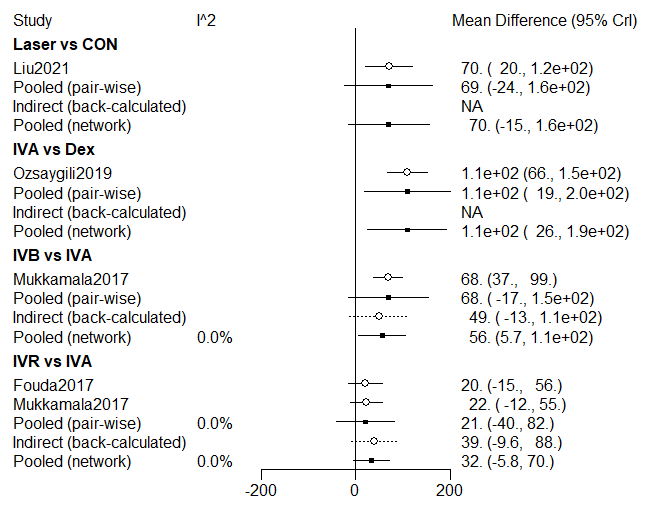

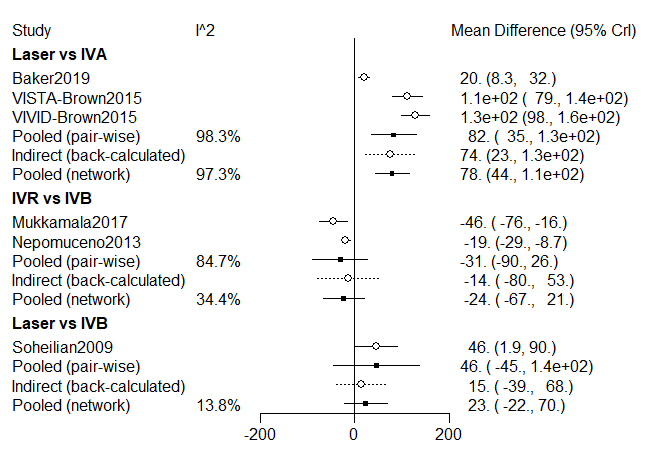

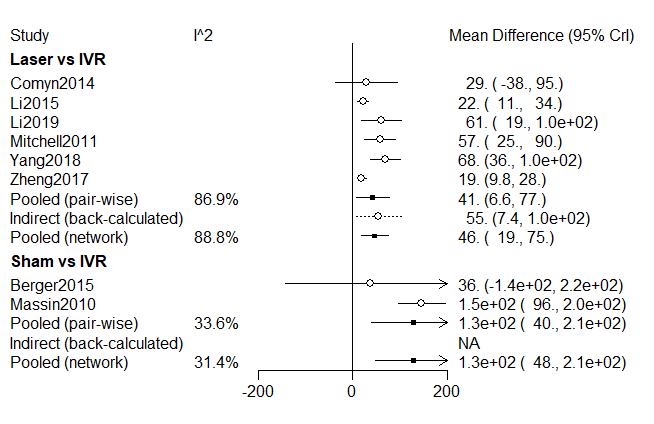


- 1. **All population at 2-year follow-up**


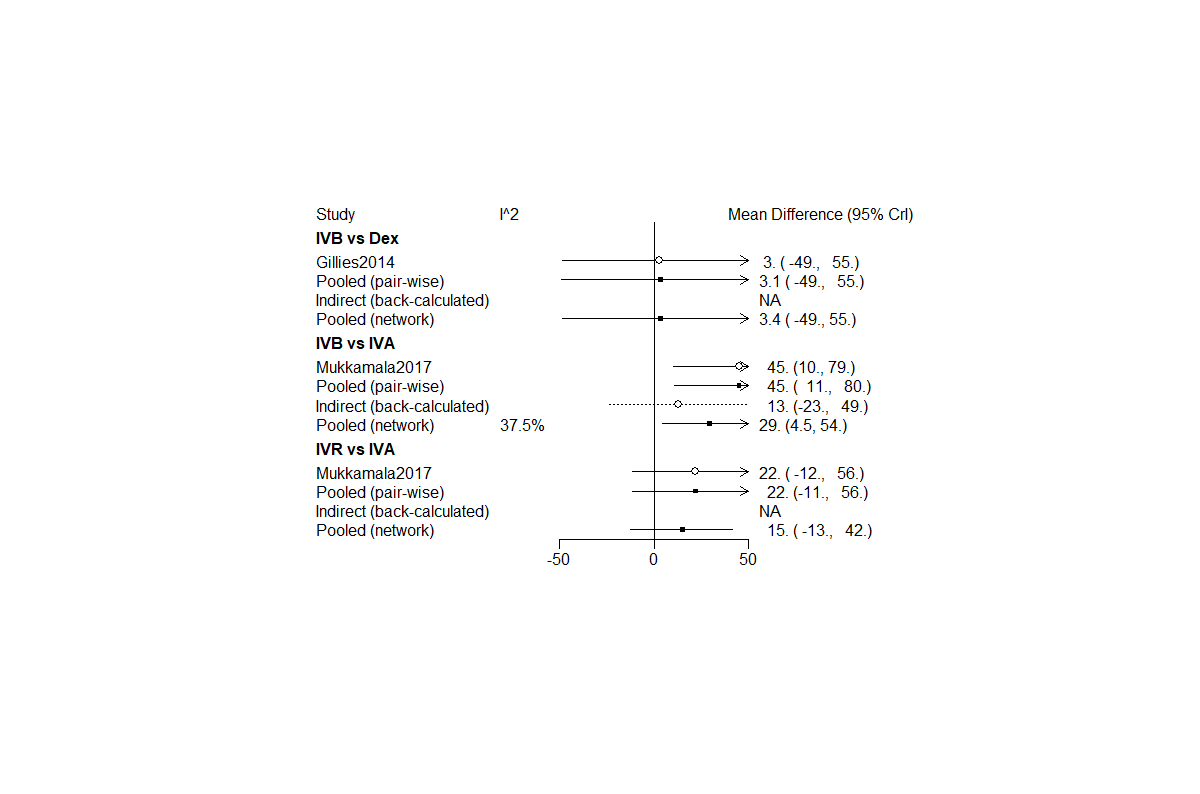

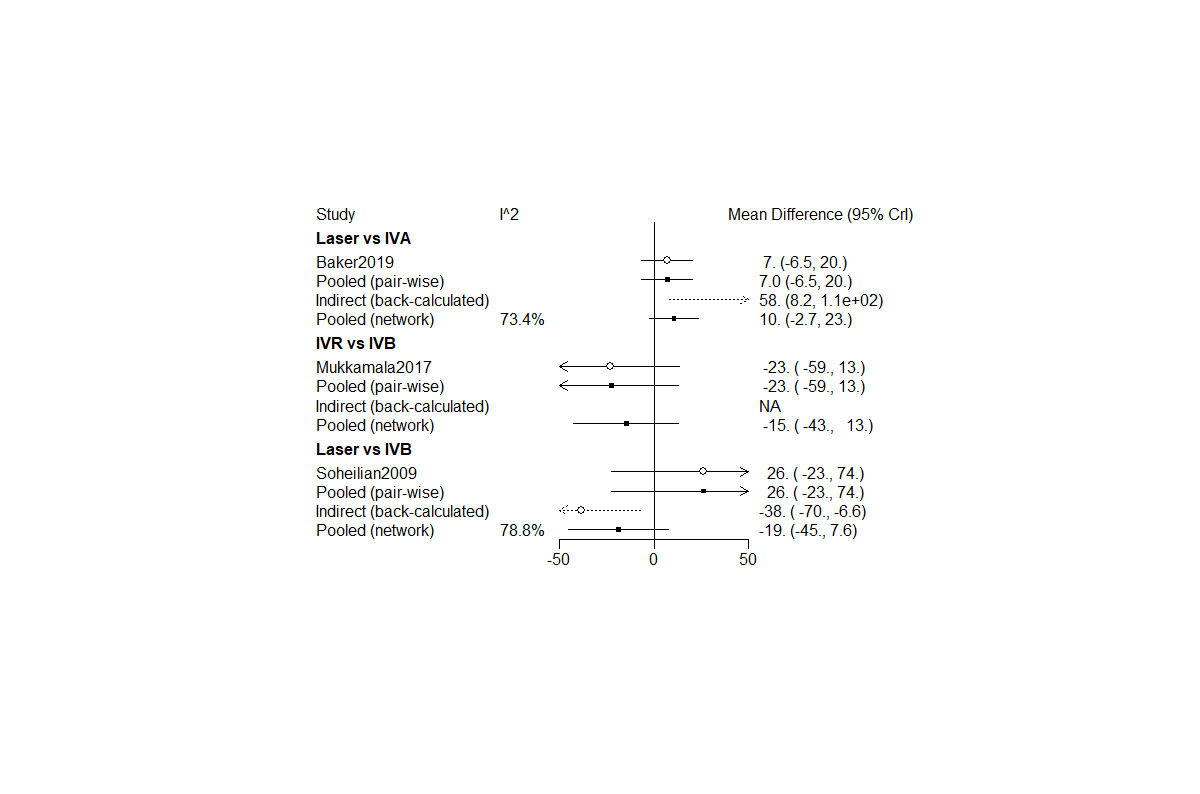


- 1. **Population with worse baseline VA at 1-year follow-up**


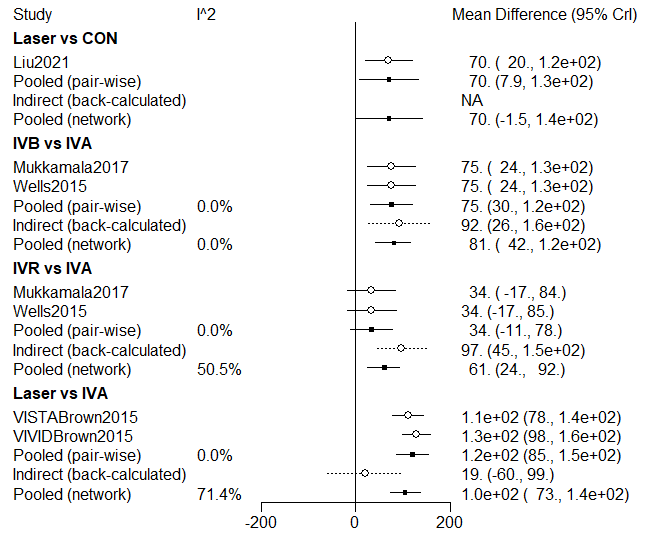

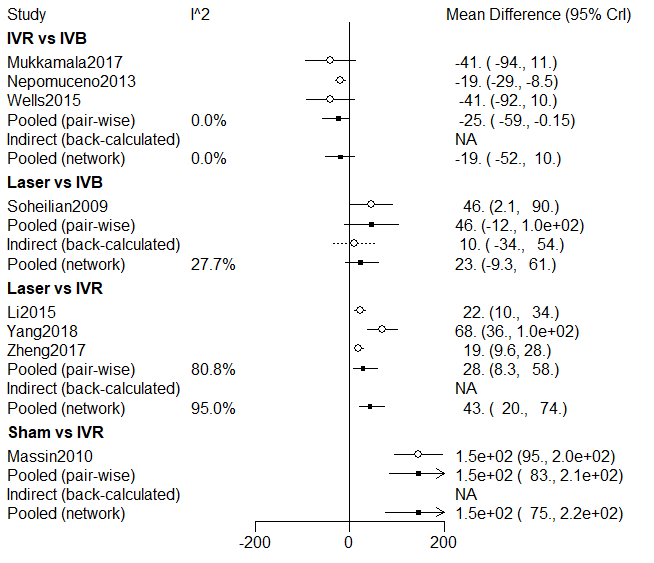


## Adverse events

### Overall heterogeneity and consistency

|  | **Overall *I^2^*** | **DIC** | Differences of DIC [should be less than 5] |
| --- | --- | --- | --- |
| **Serious adverse events in all population at 1-year follow-up** | | | |
| Fixed-effects model Consistency model | 7% | 31.46706 | 0.89877 |
| Fixed-effects model Non-consistency model | 7% | 32.36583 | – |
| **Ocular adverse events in all population at 1-year follow-up** | | | |
| Fixed-effects model Consistency model | 45% | 44.93695 | 0.06709 |
| Fixed-effects model Non-consistency model | 45% | 45.00404 | – |

Note: Fixed-effects consistency model was performed for final analysis.

### Forest plots with heterogeneity test

- 1. **Serious adverse events in all population at 1-year follow-up**


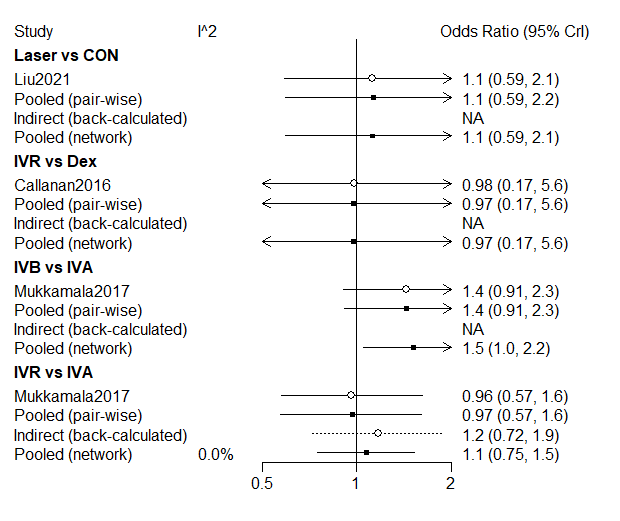

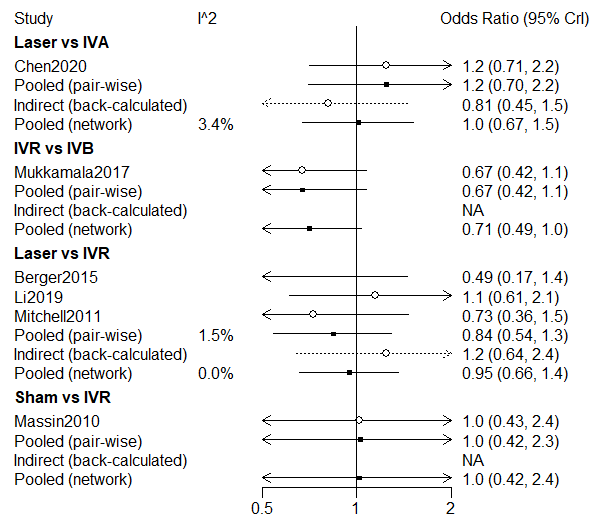


- 1. **Ocular adverse events in all population at 1-year follow-up**


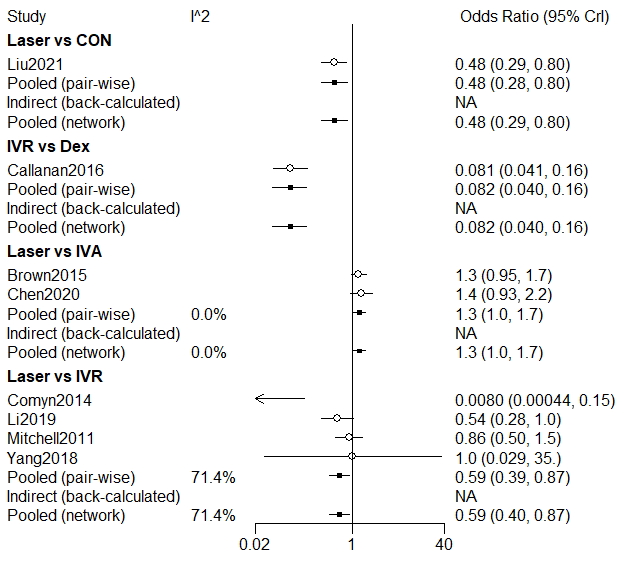

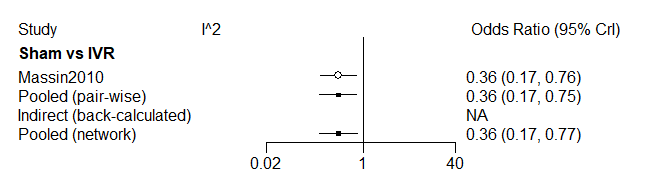


# Appendix 7 Node-split plot, trace plot and density plot

CON, conbercept; Dex, dexamethasone implant; IVA, aflibercept; IVB, bevacizumab; IVR, ranibizumab; Laser, laser; Sham, placebo.

## Mean change in BCVA measured by ETDRS letters from baseline

1. **All population at 1-year follow-up**
   1. **Node-split plot**


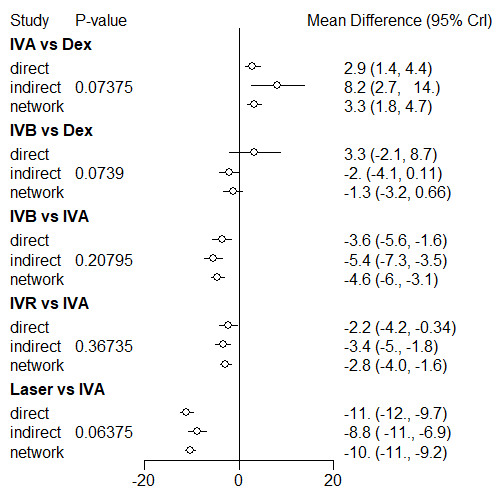

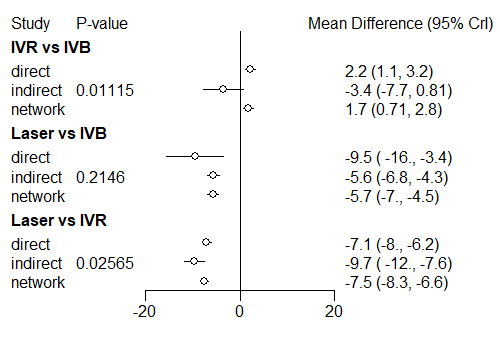


- 1. **Trace plot and density plot**

PSRF=1.000307.


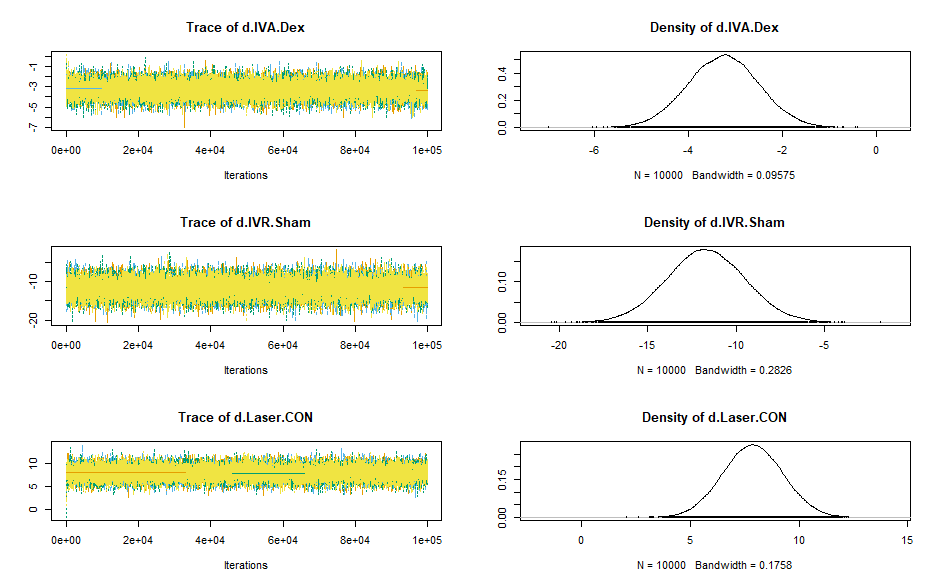

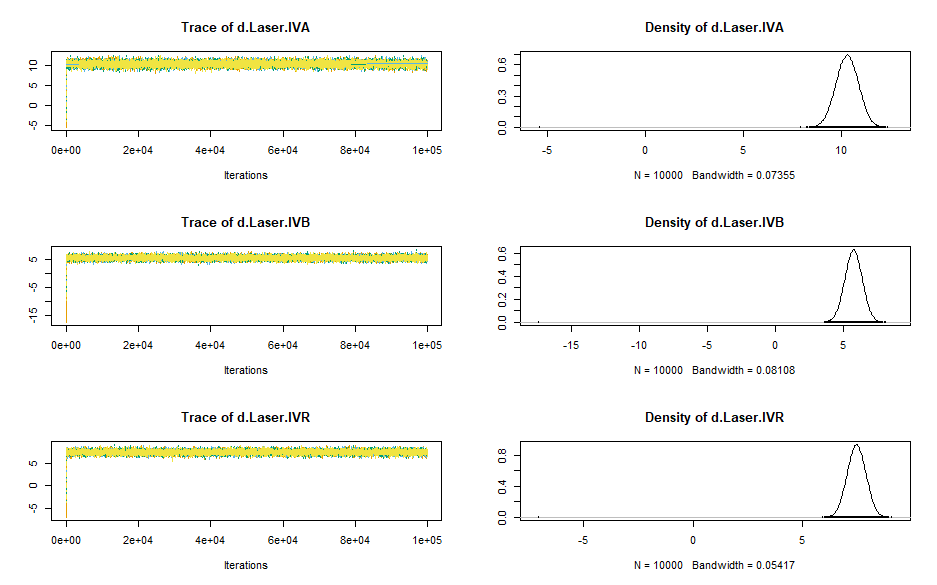

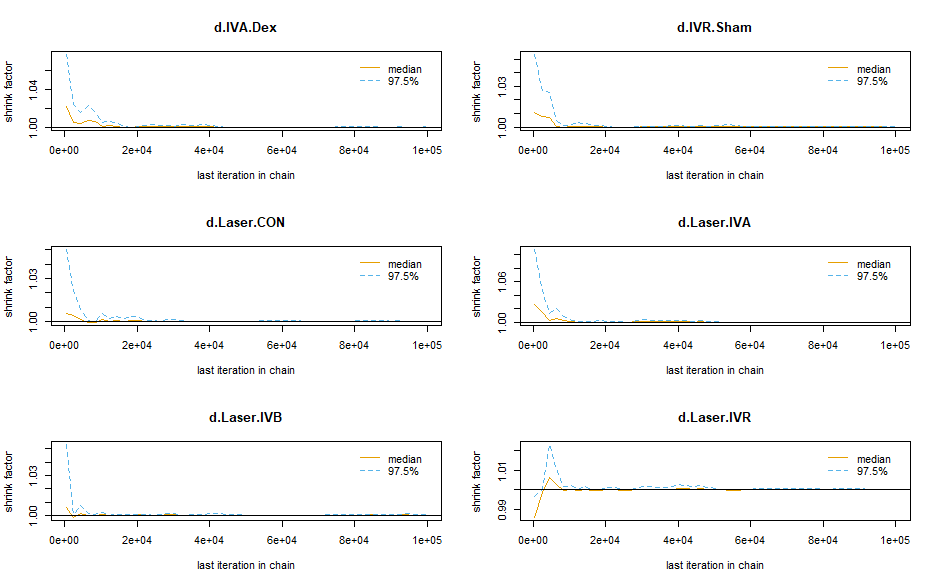


1. **All population at 2-year follow-up**
   1. **Node-split plot**


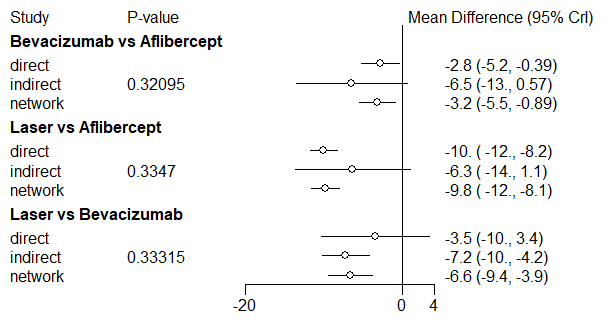


- 1. **Trace plot and density plot**

PSRF=1.000057.


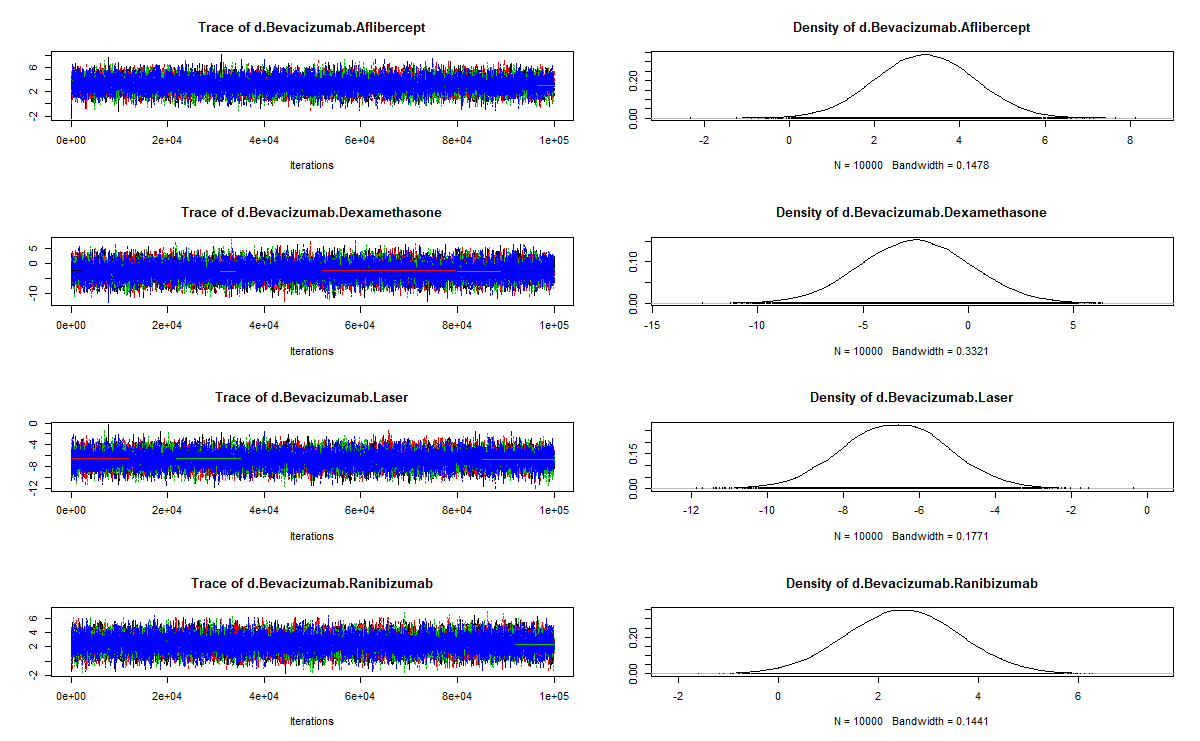

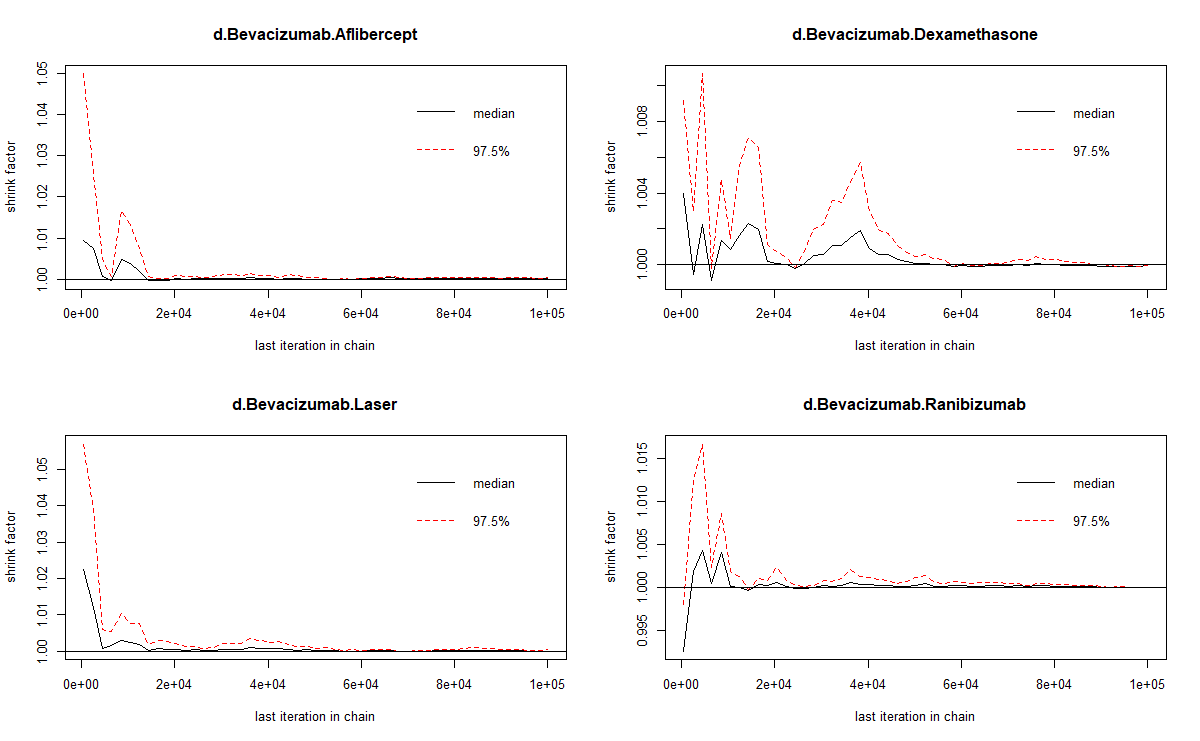


1. **Population with worse baseline VA at 1-year follow-up**
   1. **Node-split plot**


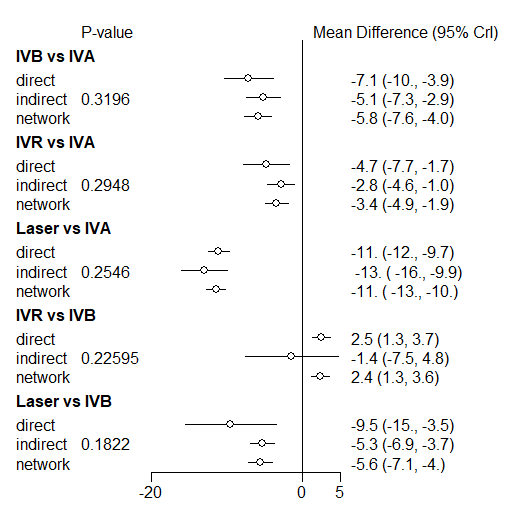

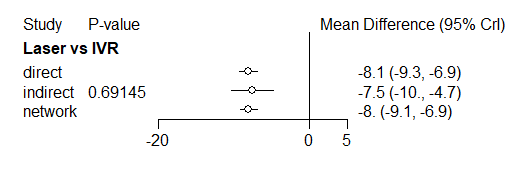


- 1. **Trace plot and density plot**

PSRF=1.000393.


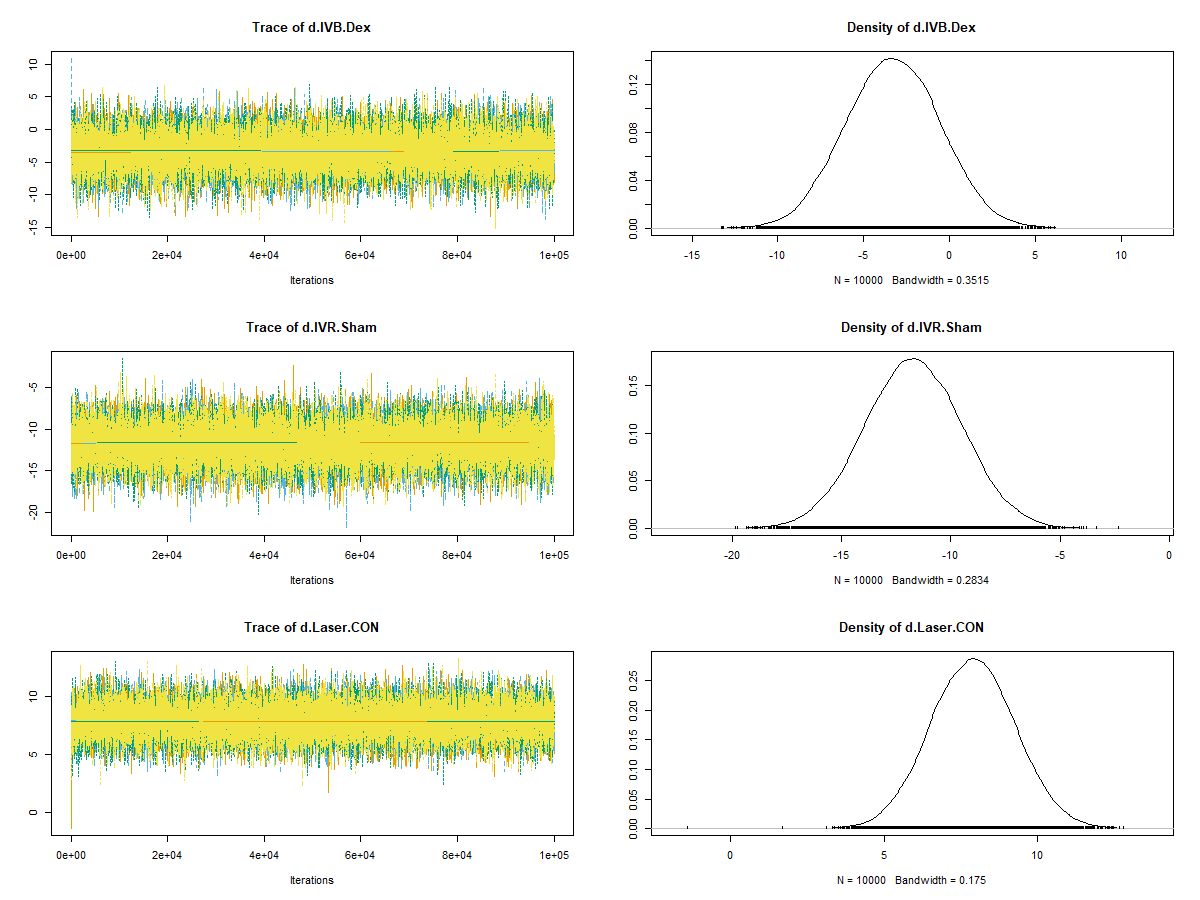

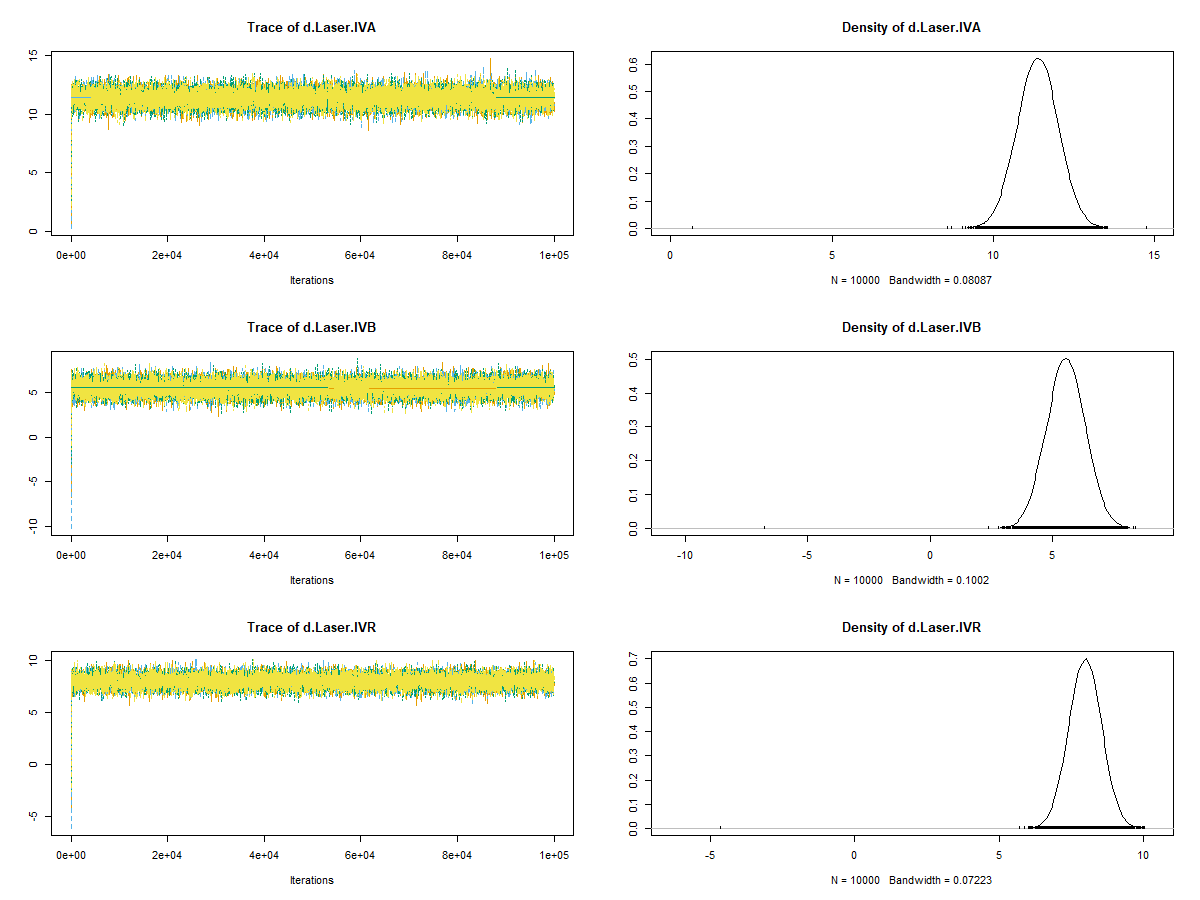

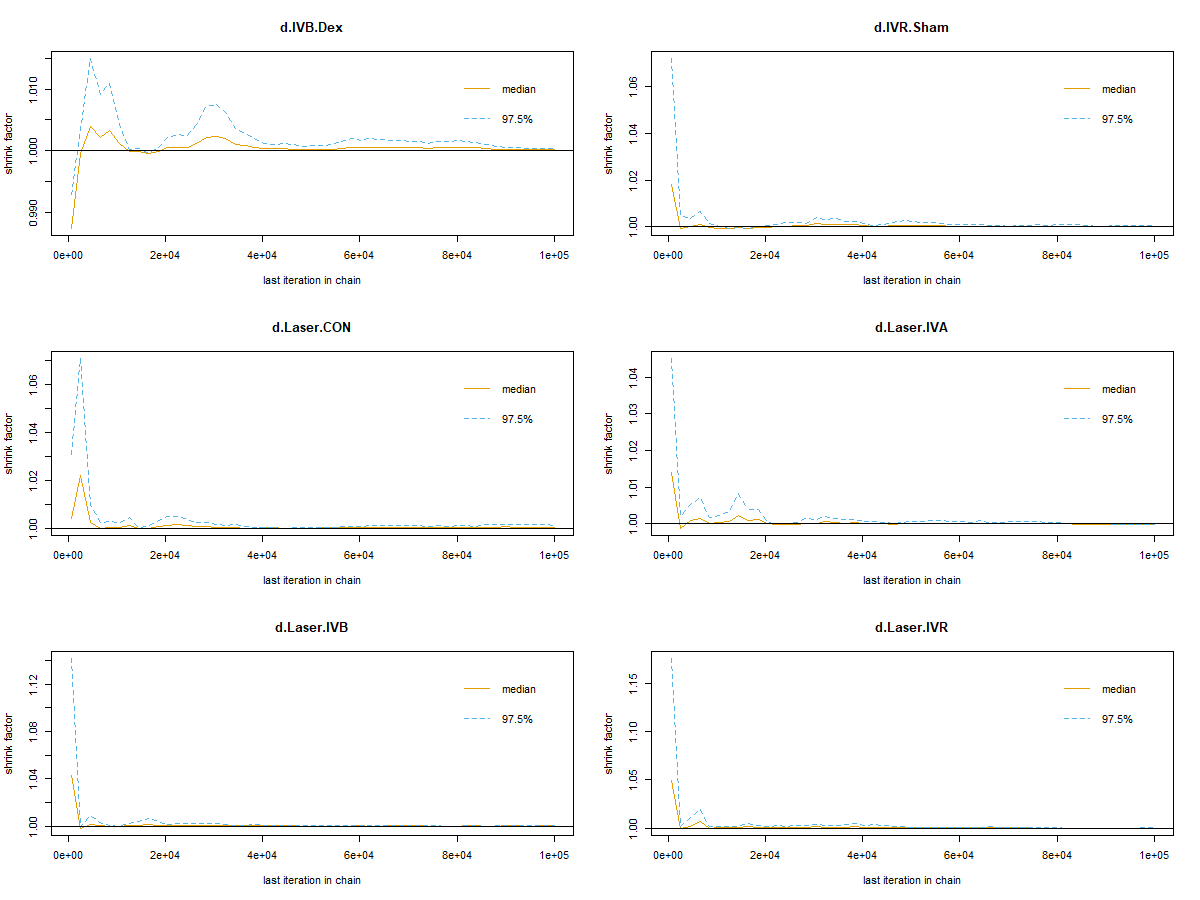


1. **Population with worse baseline VA at 2-year follow-up**
   1. **Node-split plot**


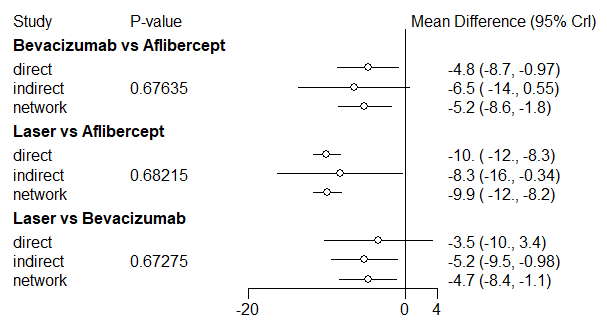


- 1. **Trace plot and density plot**

PSRF=1.000079.


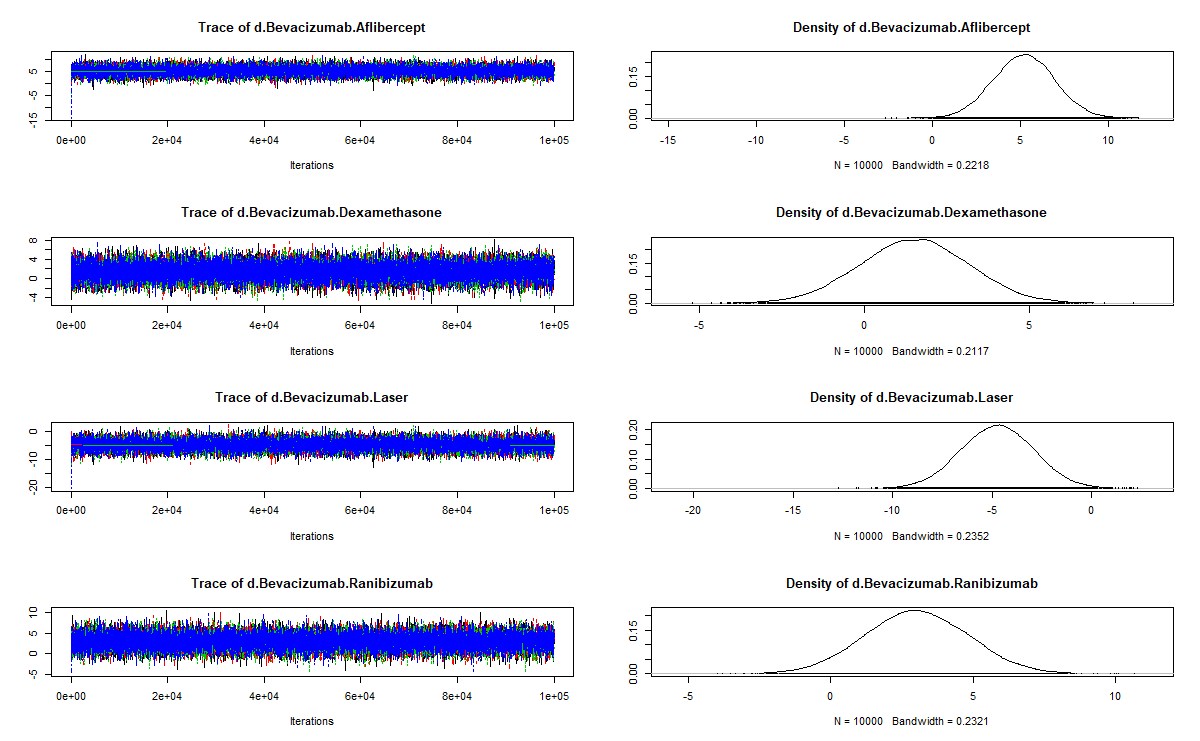

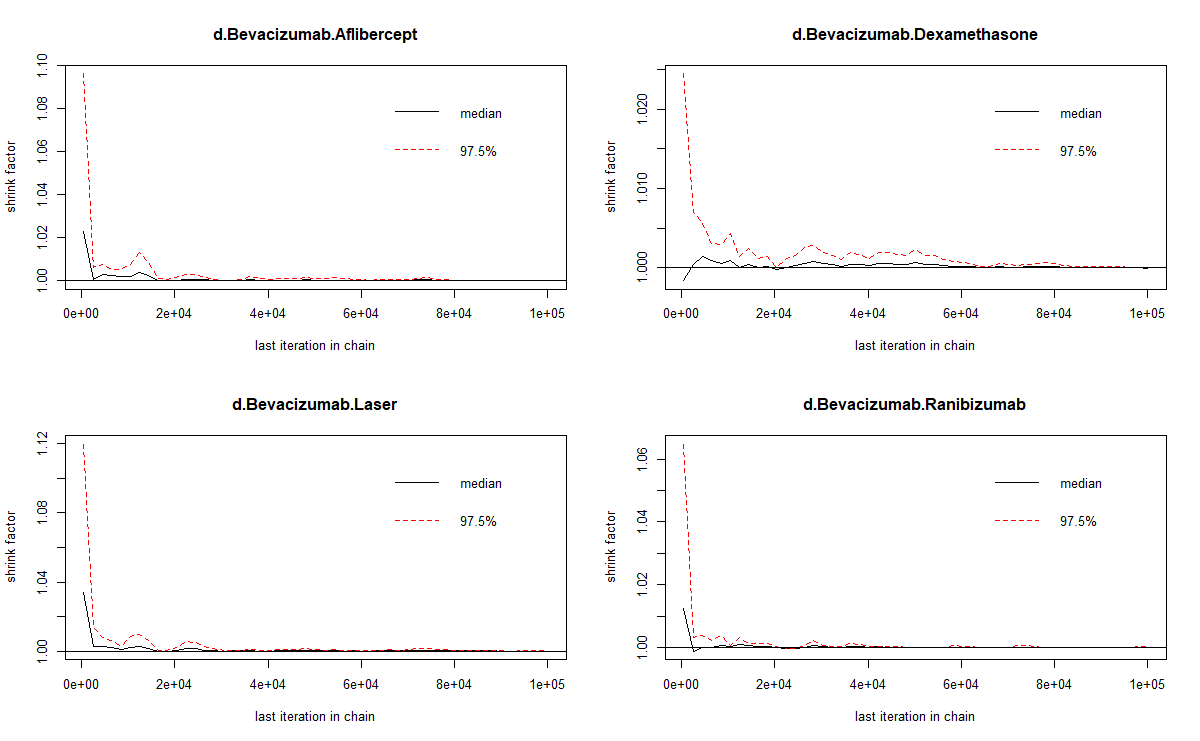


## The proportion of patients with a gain of at least 15 Early Treatment Diabetic Retinopathy Study (ETDRS) letters (3 ETDRS lines or 0.3 logMAR)

1. **All population at 1-year follow-up**
   1. **Node-split plot**


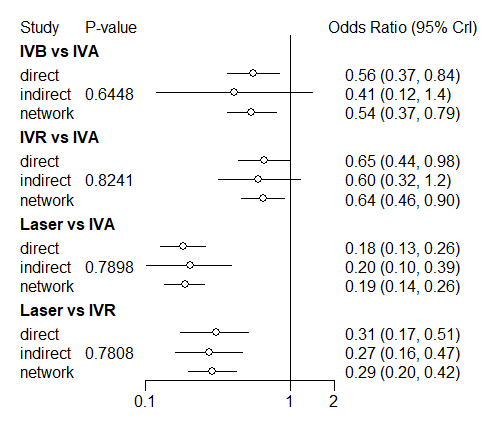


- 1. **Trace plot and density plot**

PSRF=1.000319.


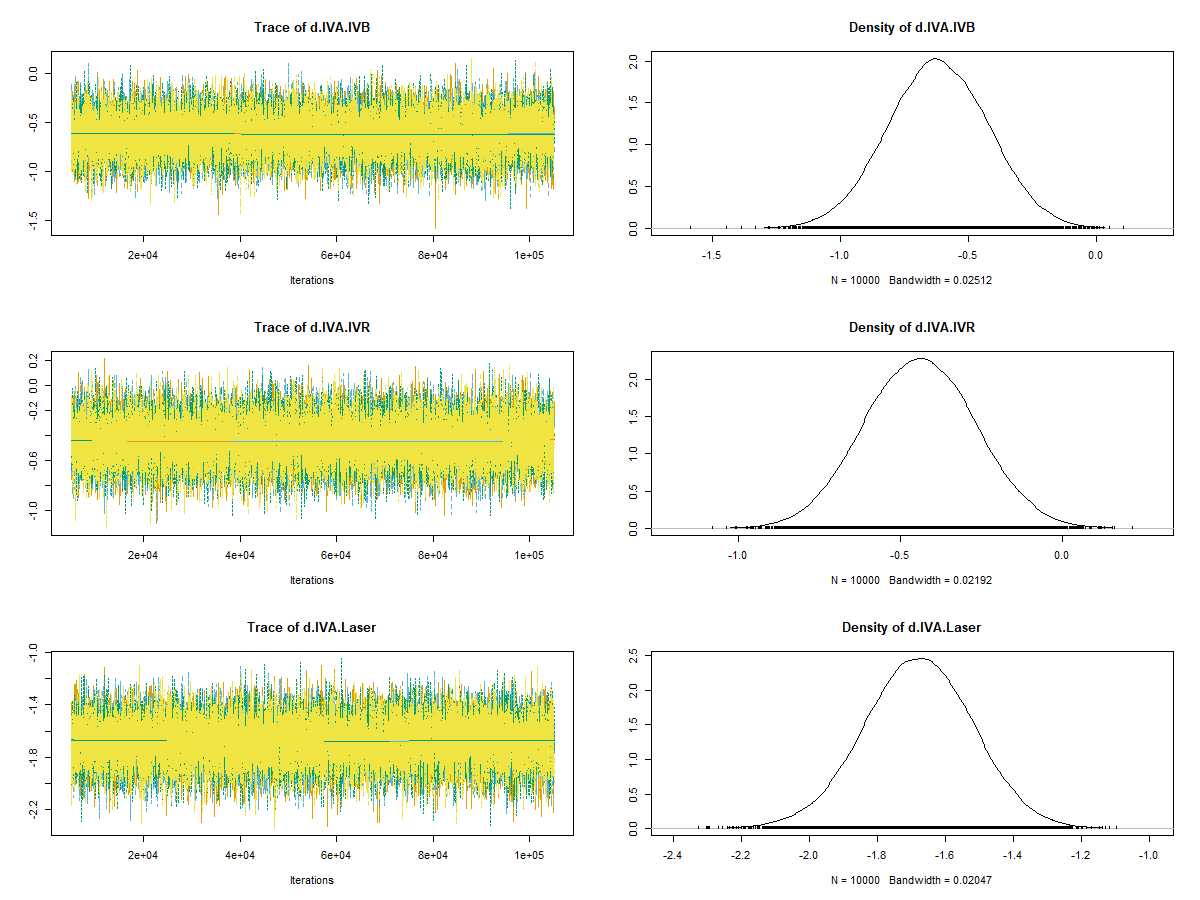

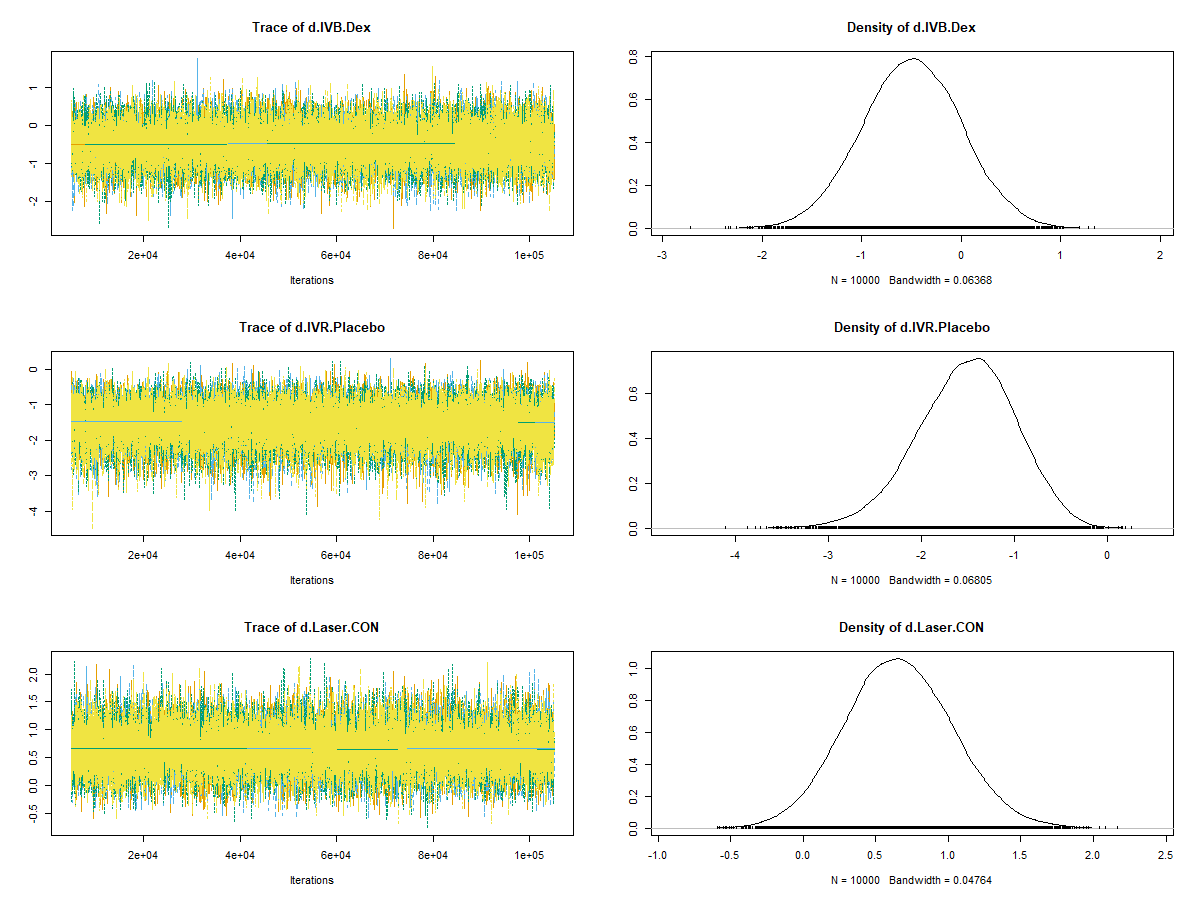

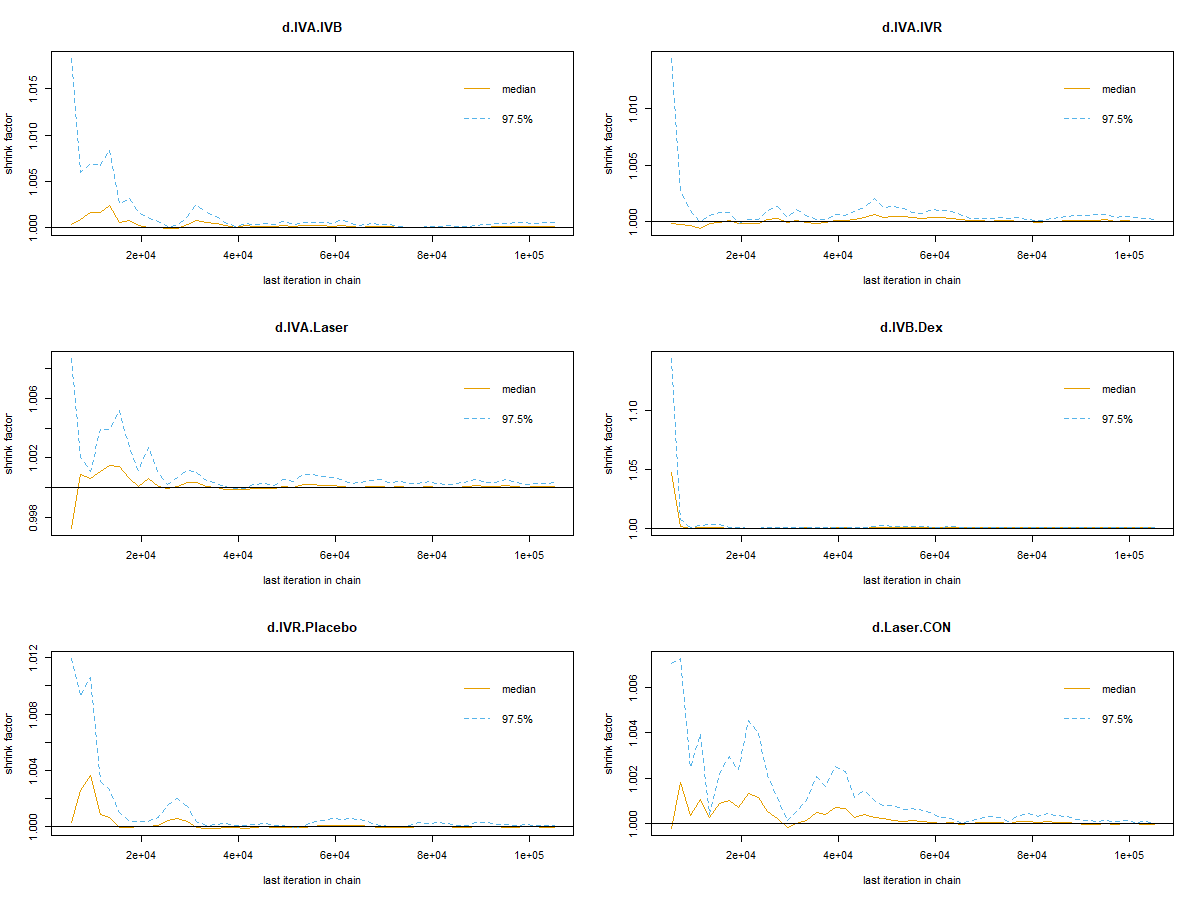


1. **All population at 2-year follow-up**
   1. **Node-split plot**


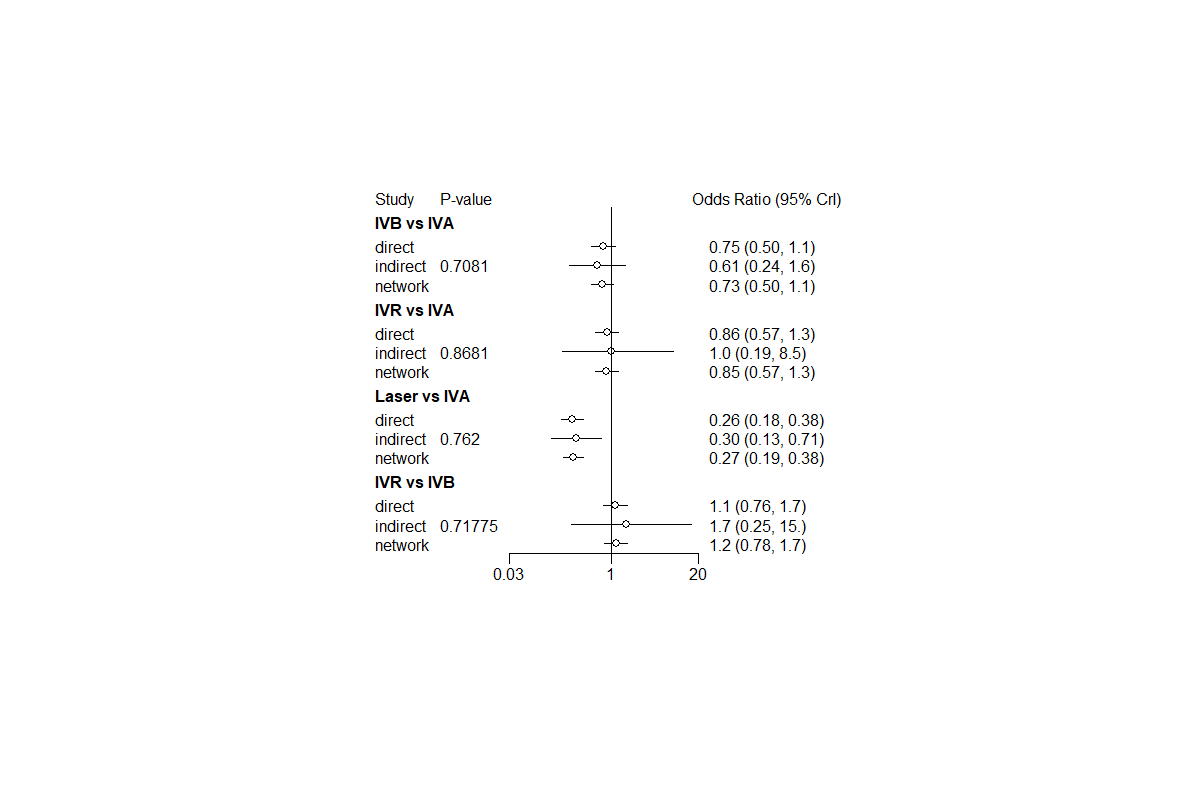

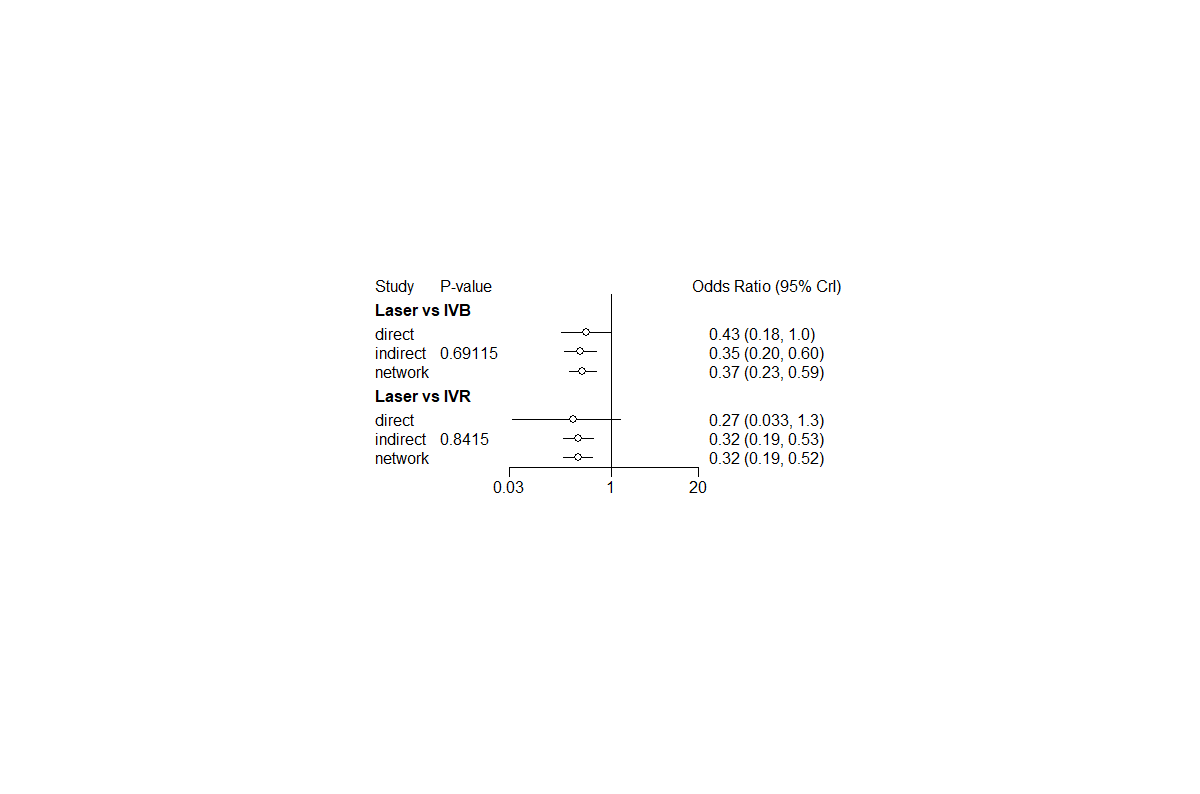


- 1. **Trace plot and density plot**

PSRF=1.00011.


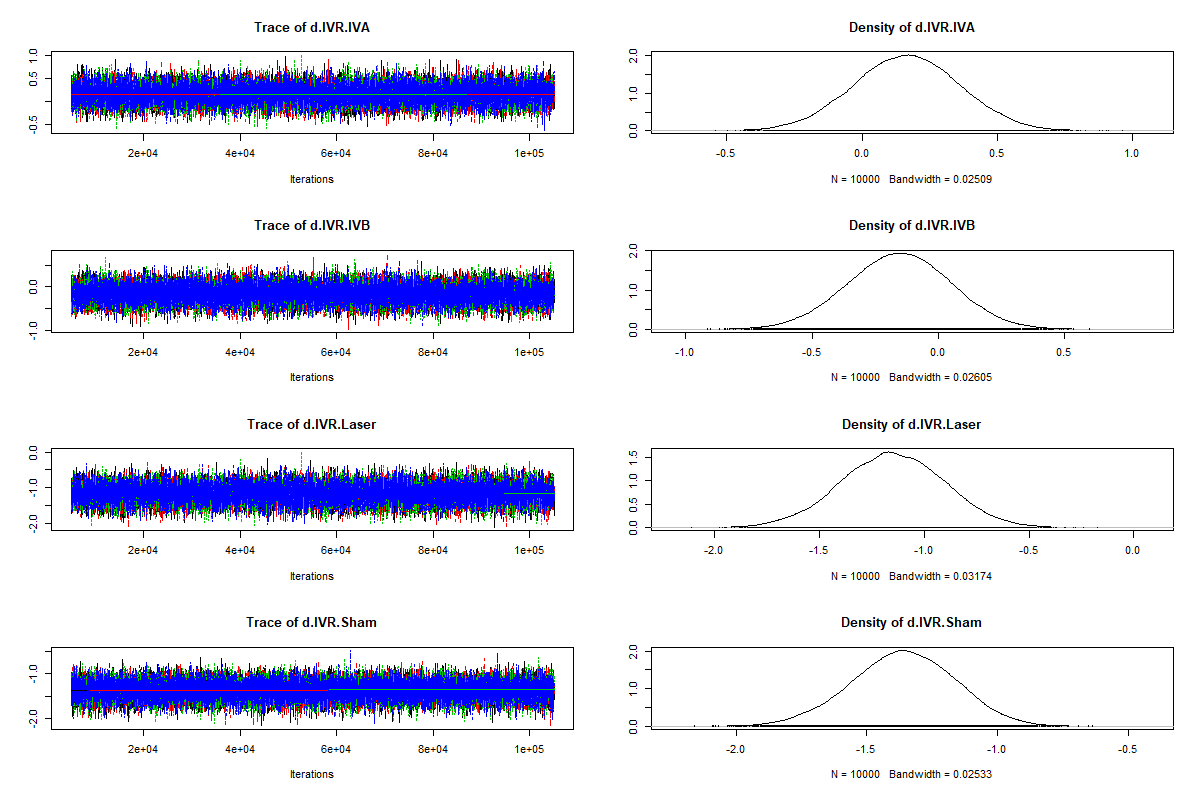

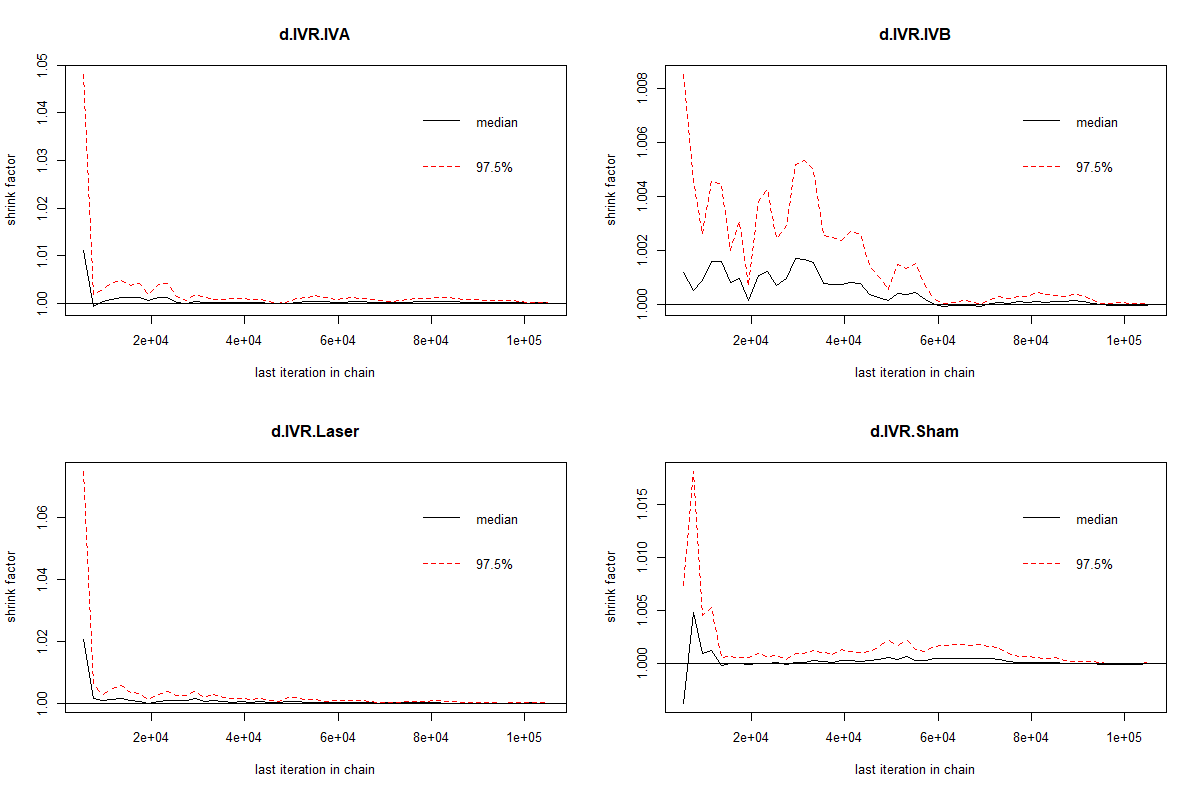


1. **Population with worse baseline VA at 1-year follow-up**
   1. **Node-split plot**

Node-split plot was not got due to lacking closed loop.

- 1. **Trace plot and density plot**

PSRF=1.000395.


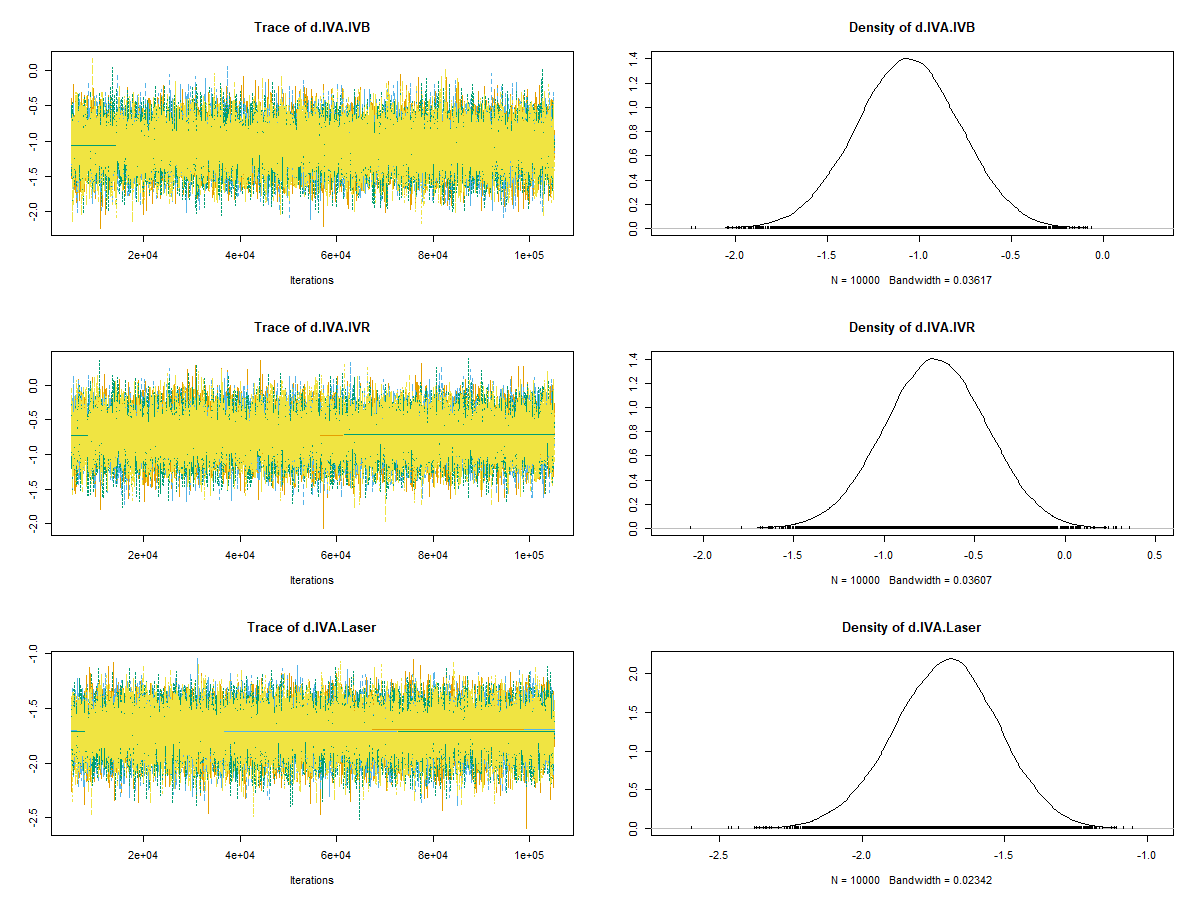

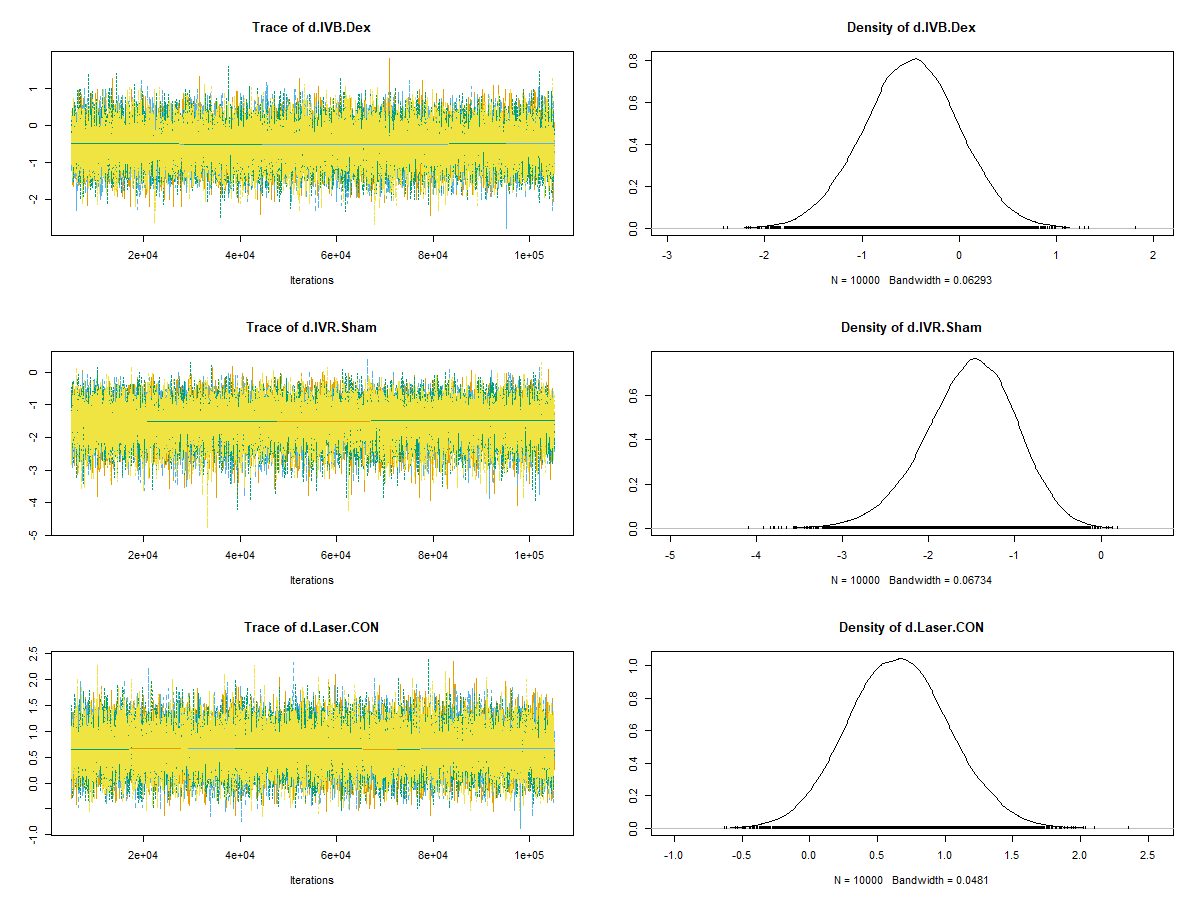

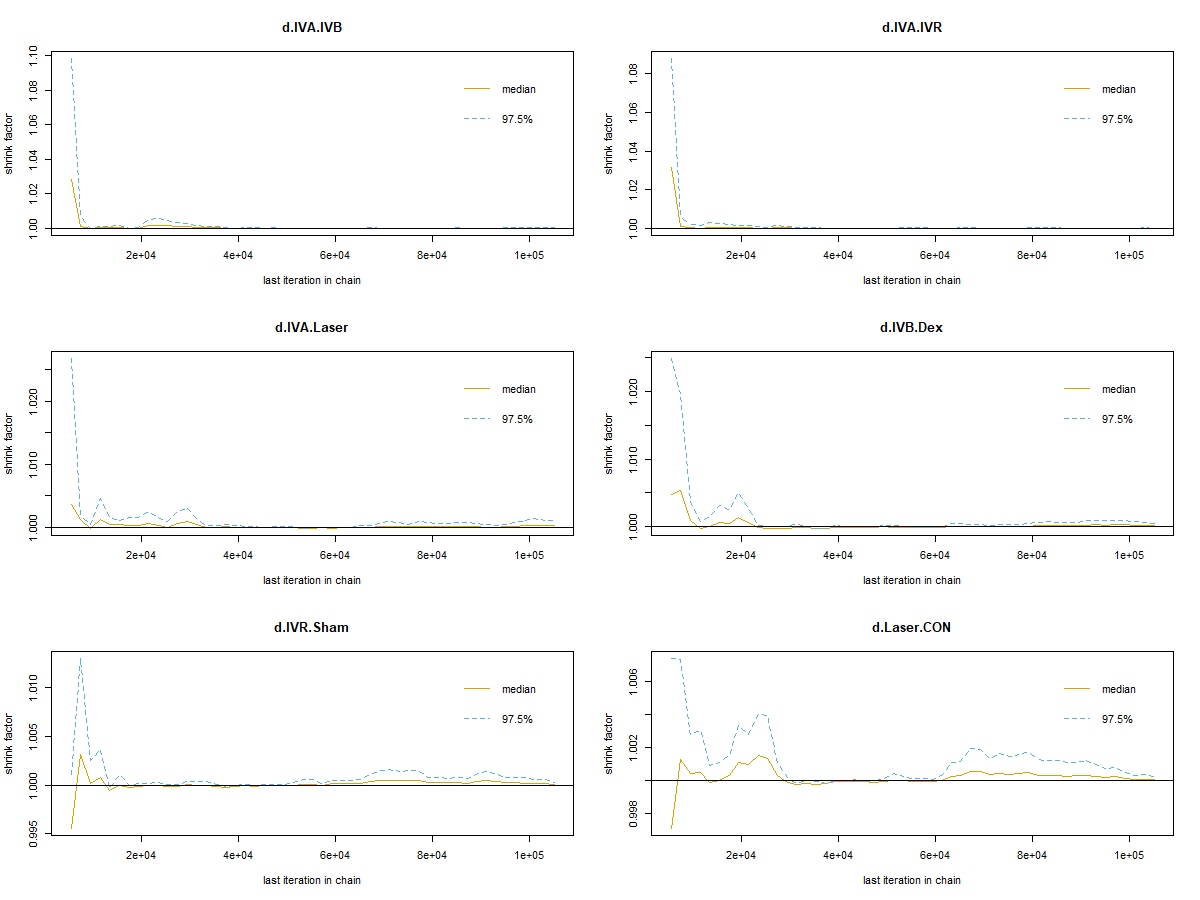


1. **Population with worse baseline VA at 2-year follow-up**
   1. **Node-split plot**


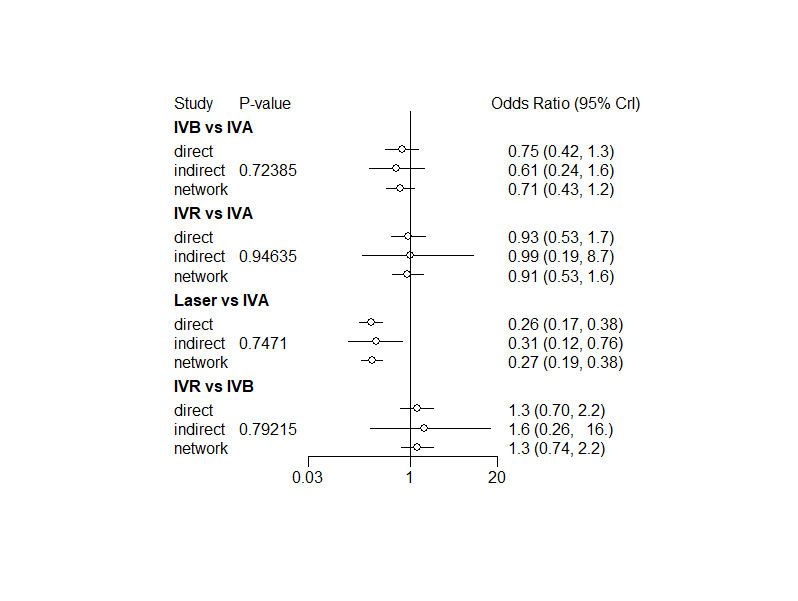

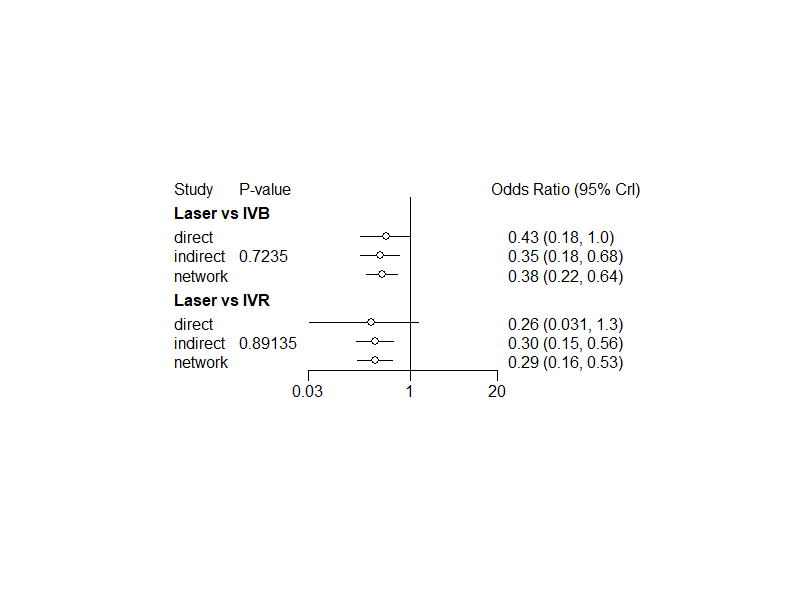


- 1. **Trace plot and density plot**

PSRF=1.000719.


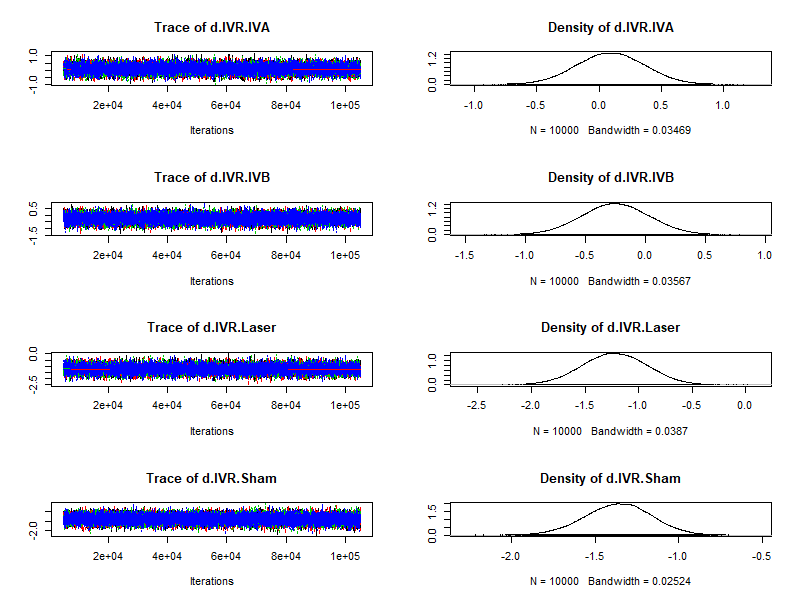

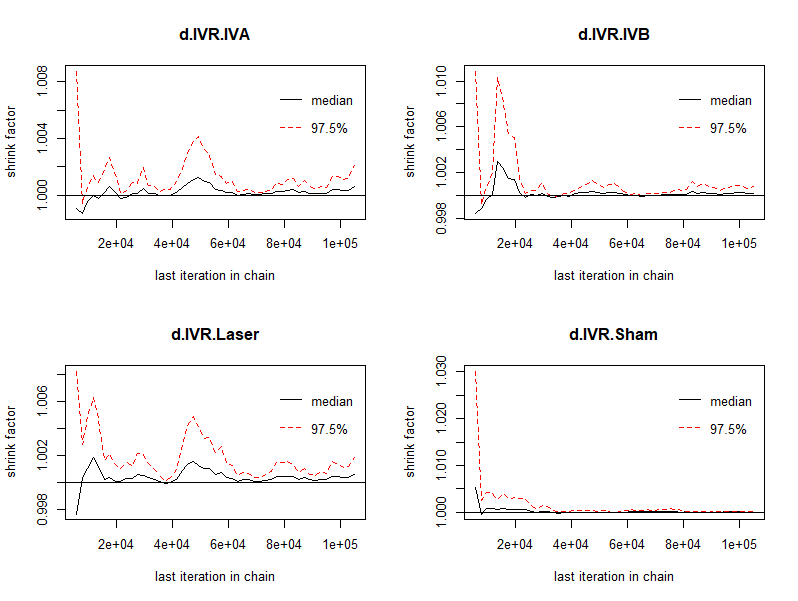


## The proportion of patients with a gain of at least 10 Early Treatment Diabetic Retinopathy Study (ETDRS) letters (2 ETDRS lines or 0.2 logMAR)

1. **All population at 1-year follow-up**
   1. **Node-split plot**


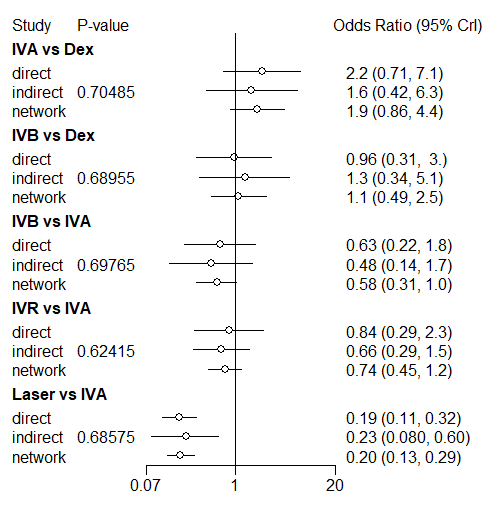

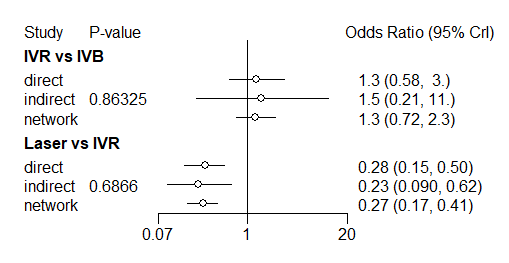


- 1. **Trace plot and density plot**

PSRF=1.002268.


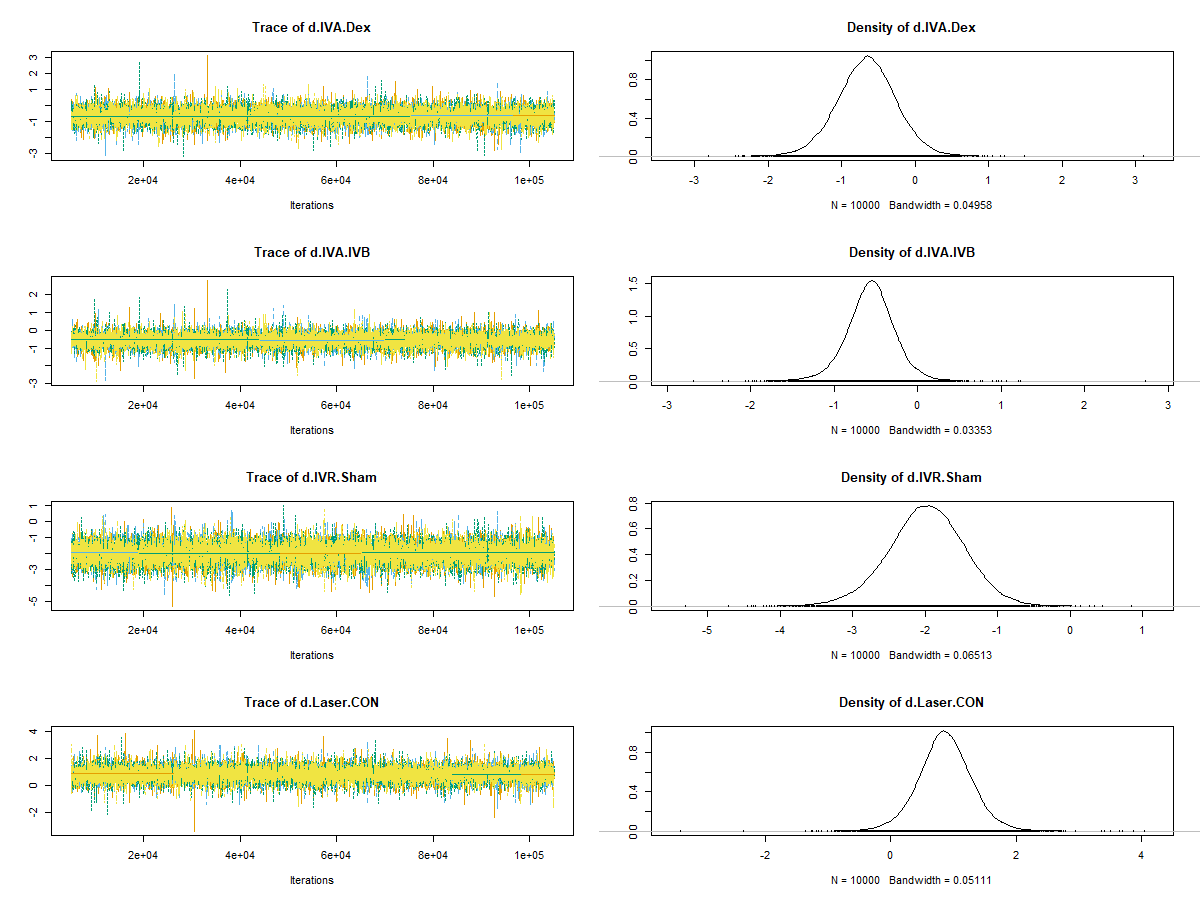

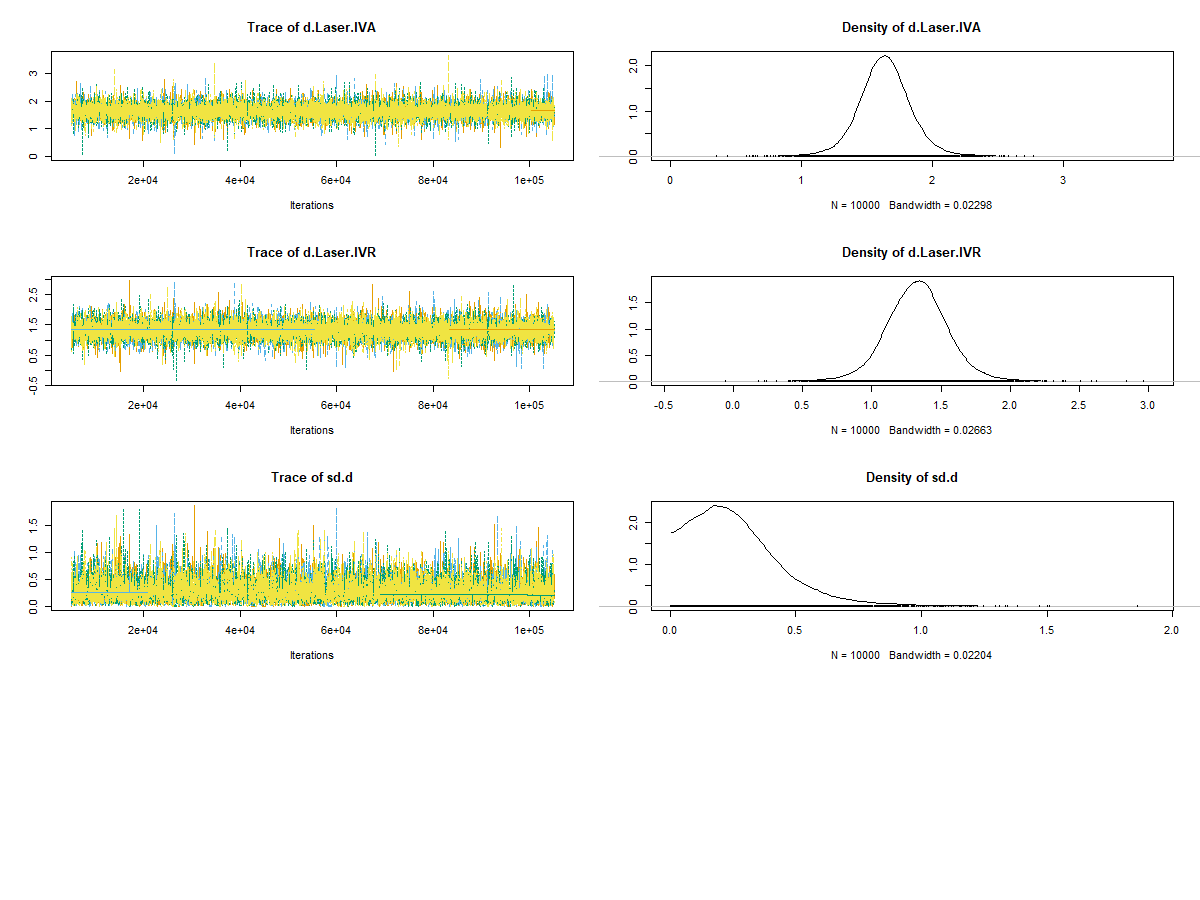

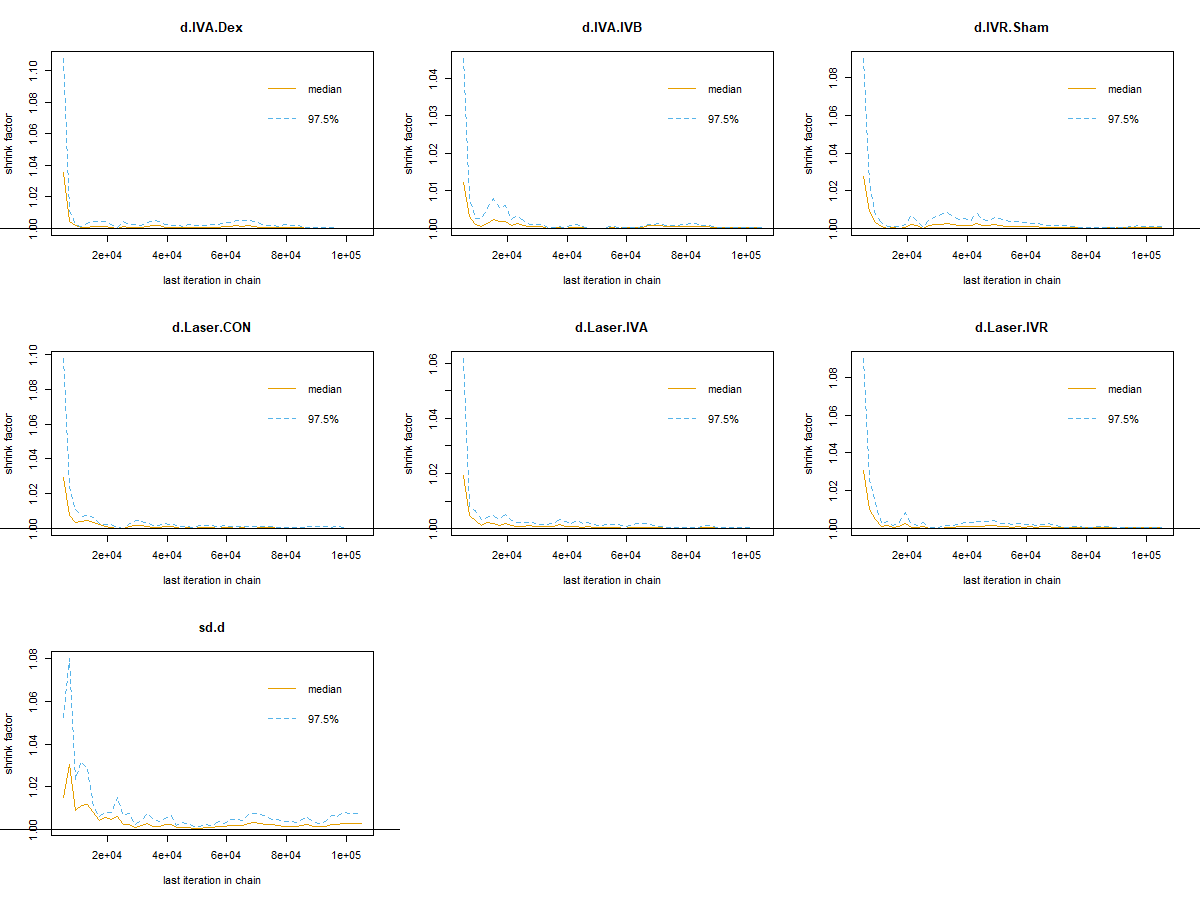


1. **All population at 2-year follow-up**
   1. **Node-split plot**


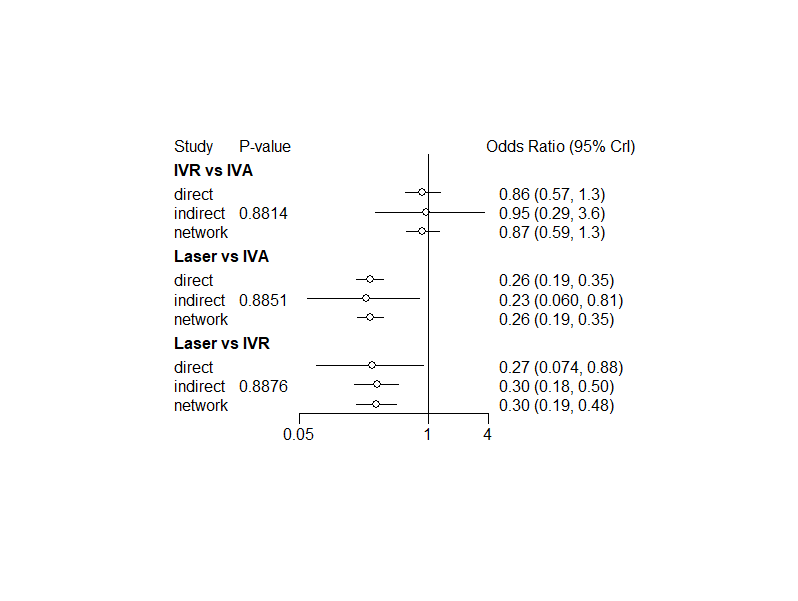


- 1. **Trace plot and density plot**

PSRF=1.00807.


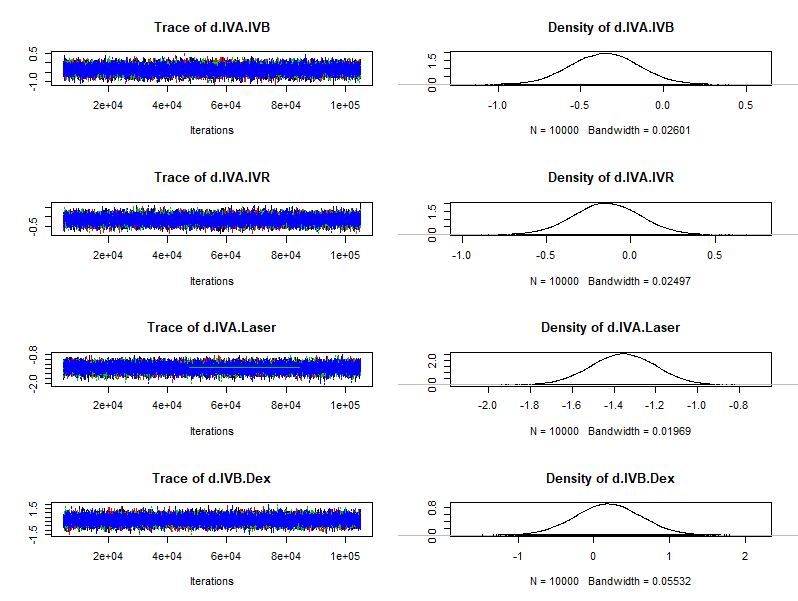

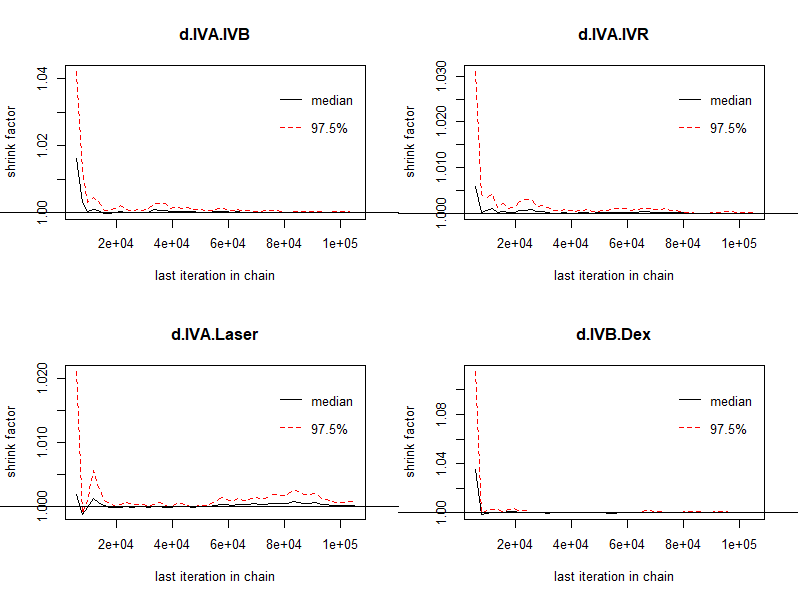


1. **Population with worse baseline VA at 1-year follow-up**
   1. **Node-split plot**

Node-split plot was not got due to lacking closed loop.

- 1. **Trace plot and density plot**

PSRF=1.000116.


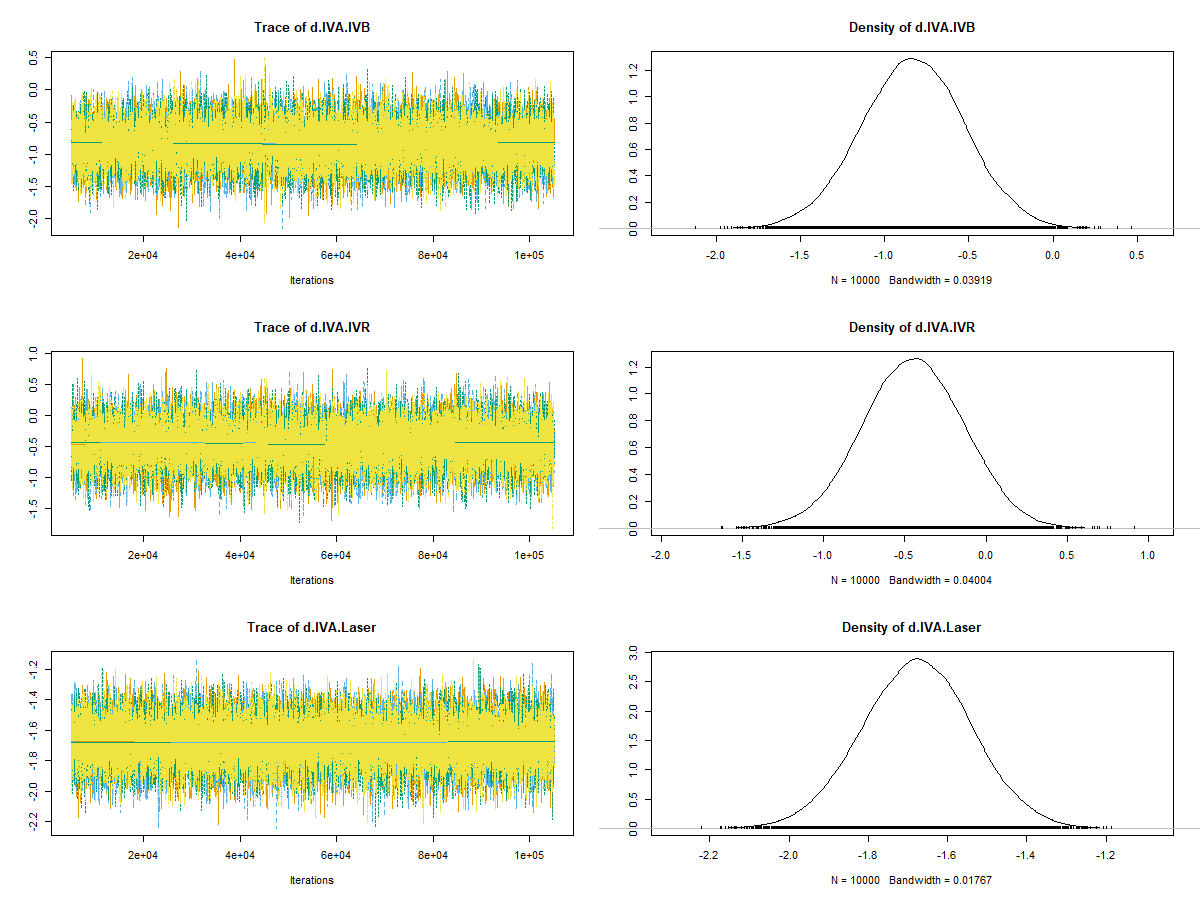

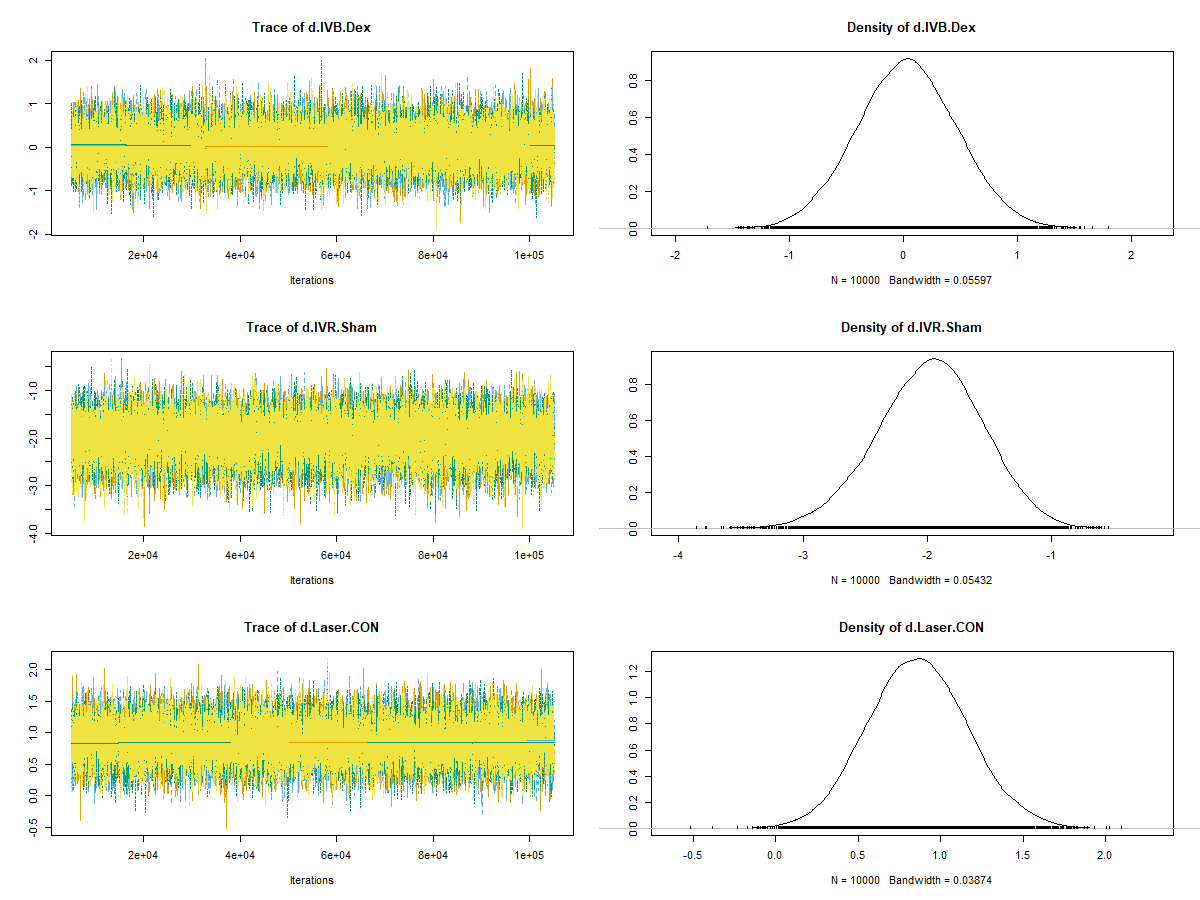

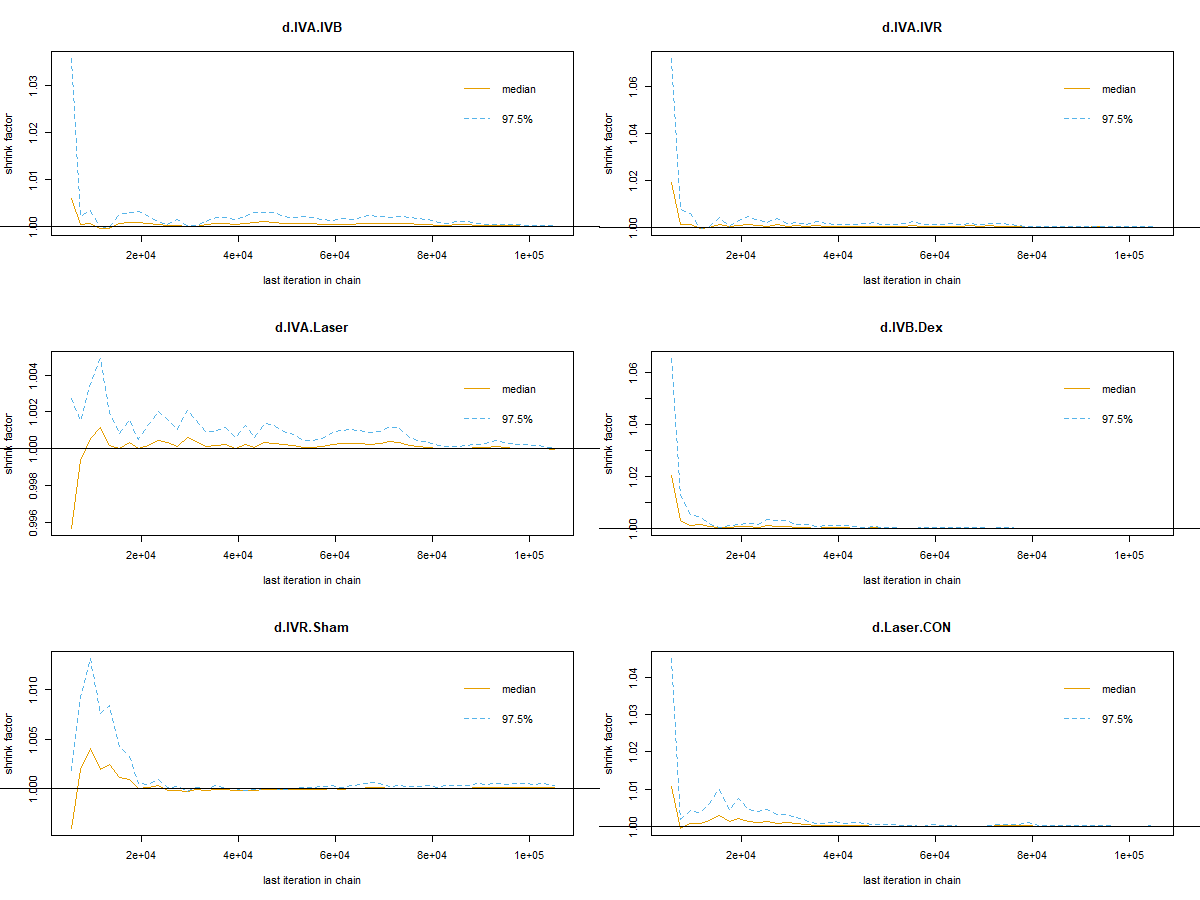


1. **Population with worse baseline VA at 2-year follow-up**
   1. **Node-split plot**


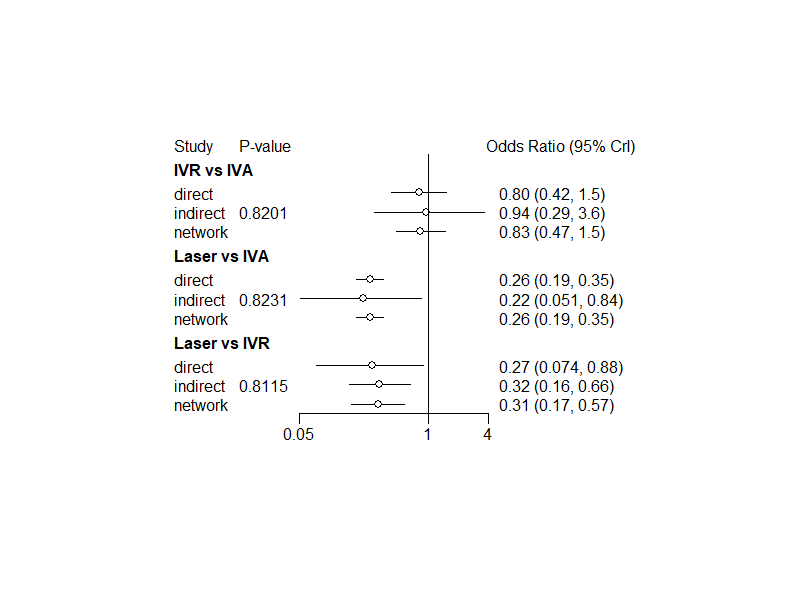


- 1. **Trace plot and density plot**

PSRF=1.000244.


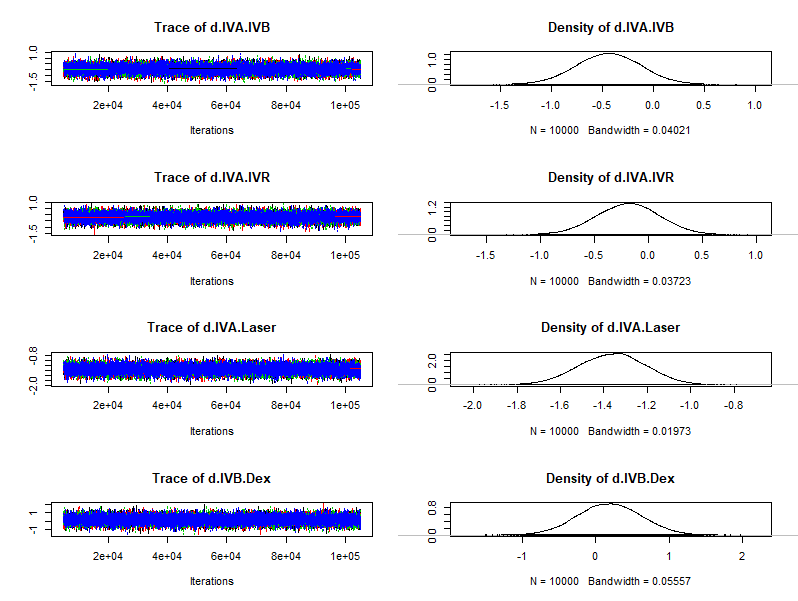

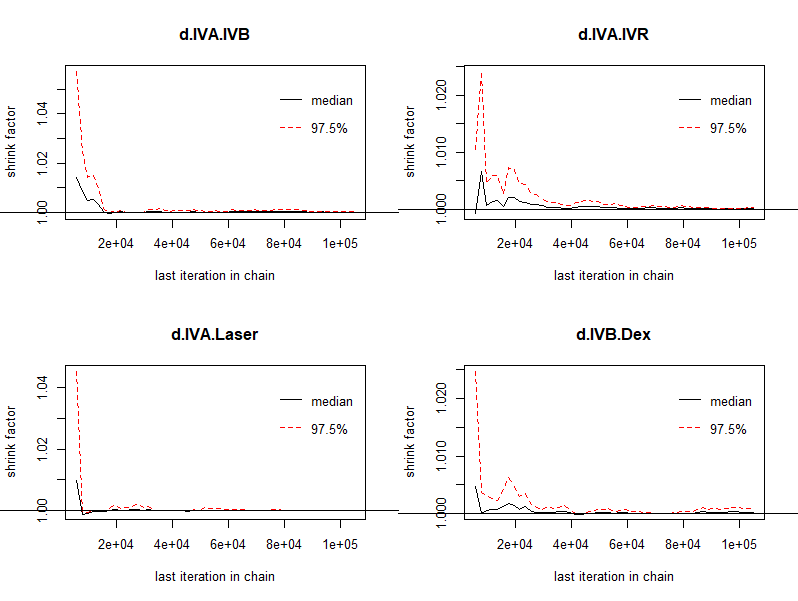


## Mean change in central retinal thickness (μm) from baseline

1. **All population at 1-year follow-up**
   1. **Node-split plot**


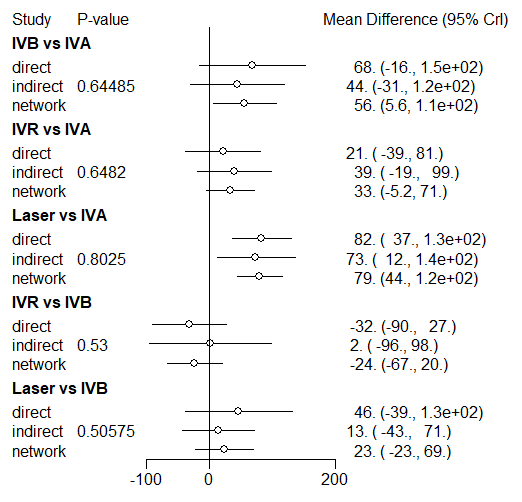

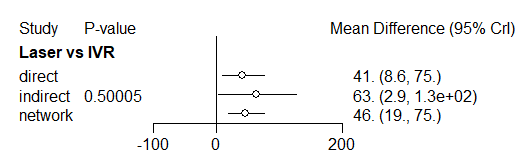


- 1. **Trace plot and density plot**

PSRF=1.000454.


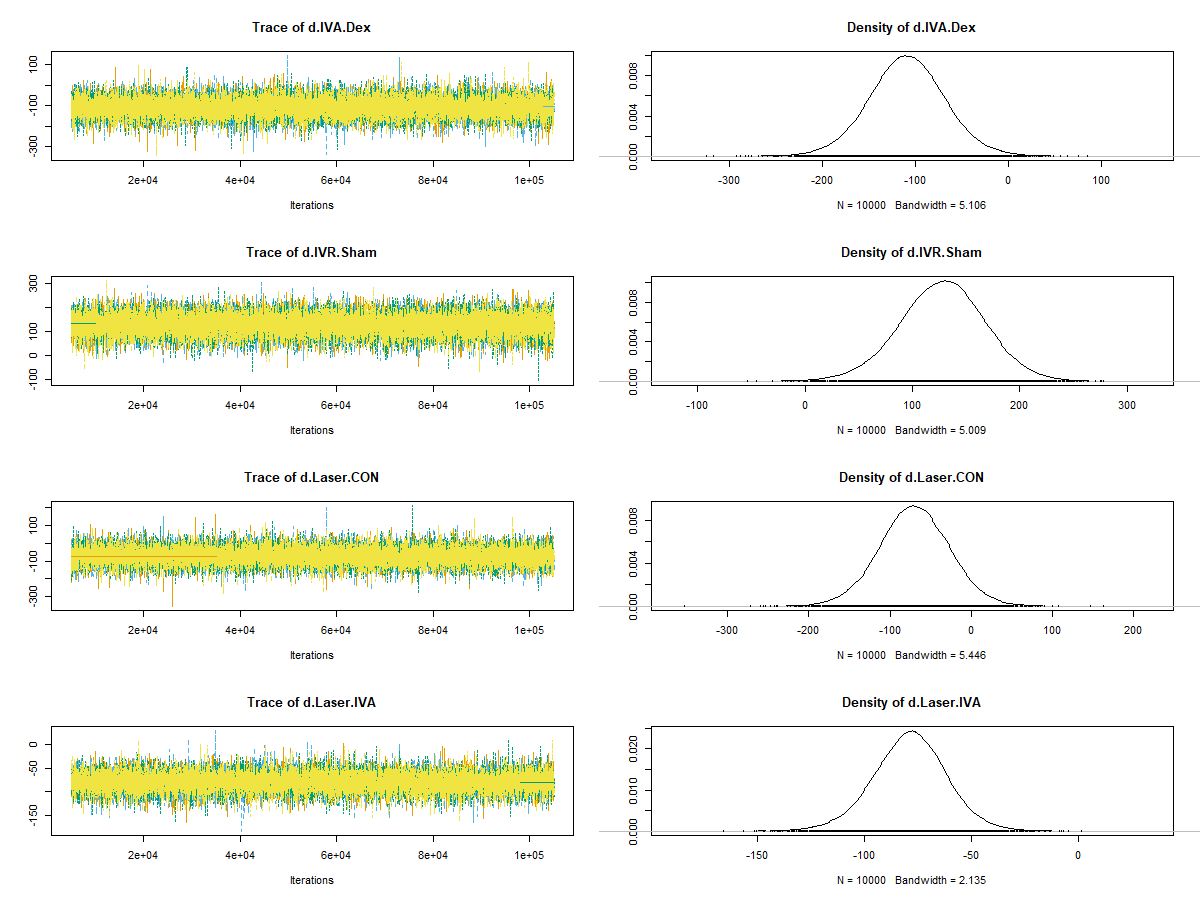

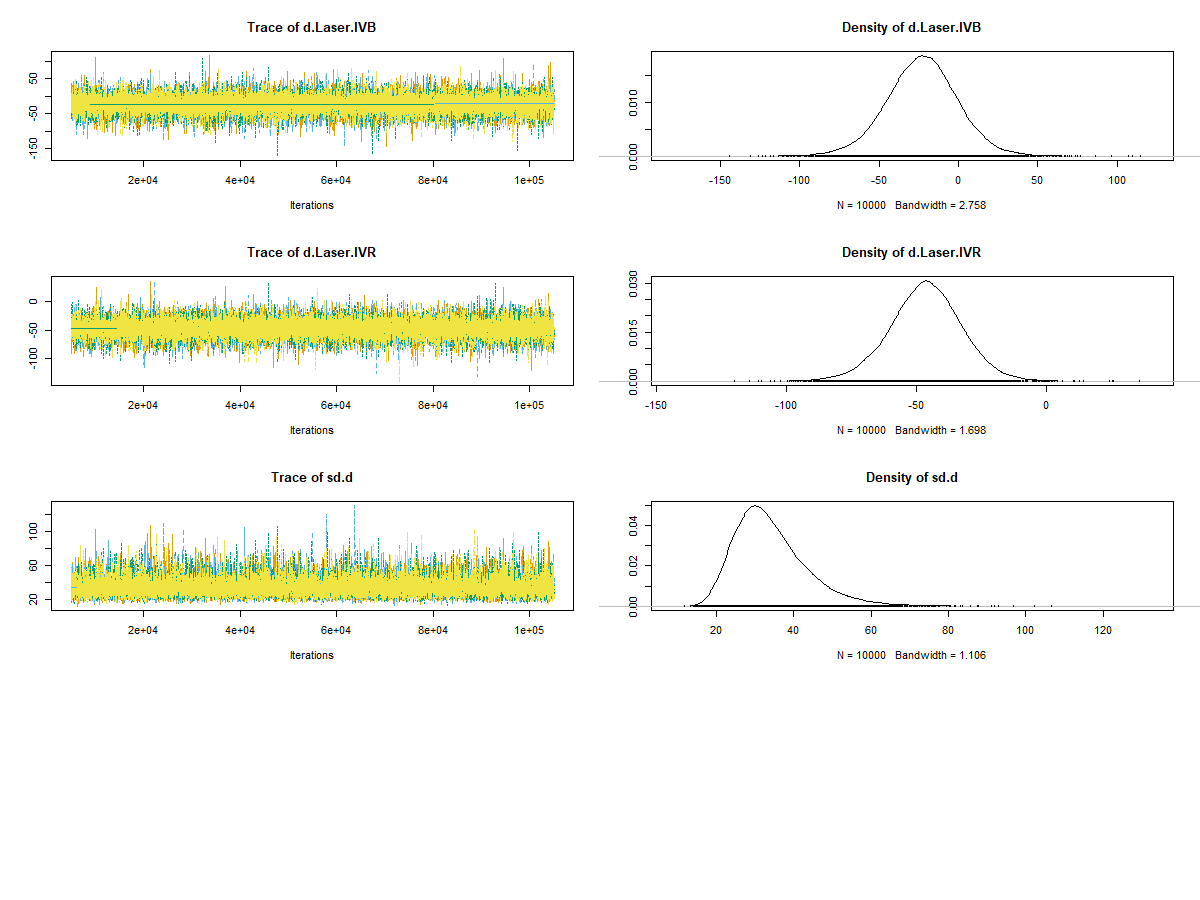

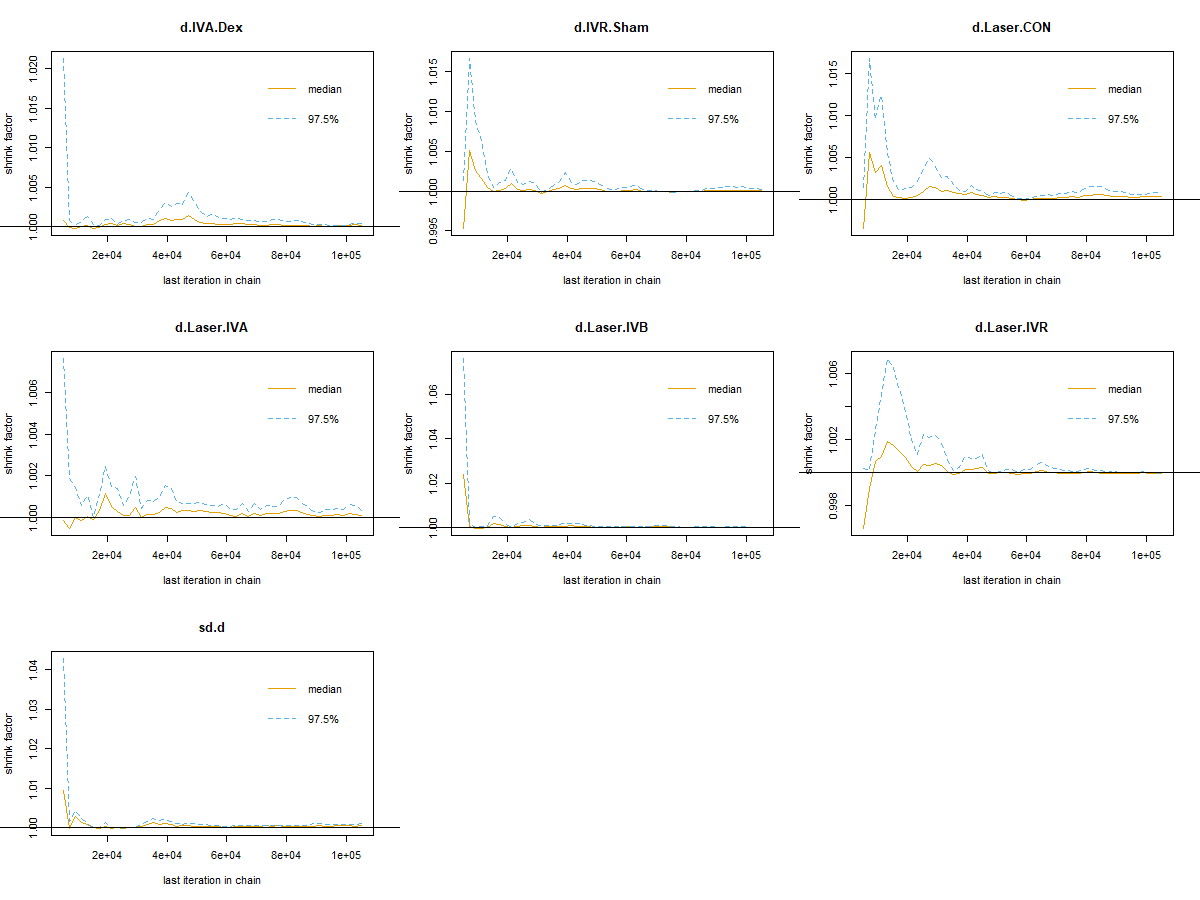


1. **All population at 2-year follow-up**
   1. **Node-split plot**


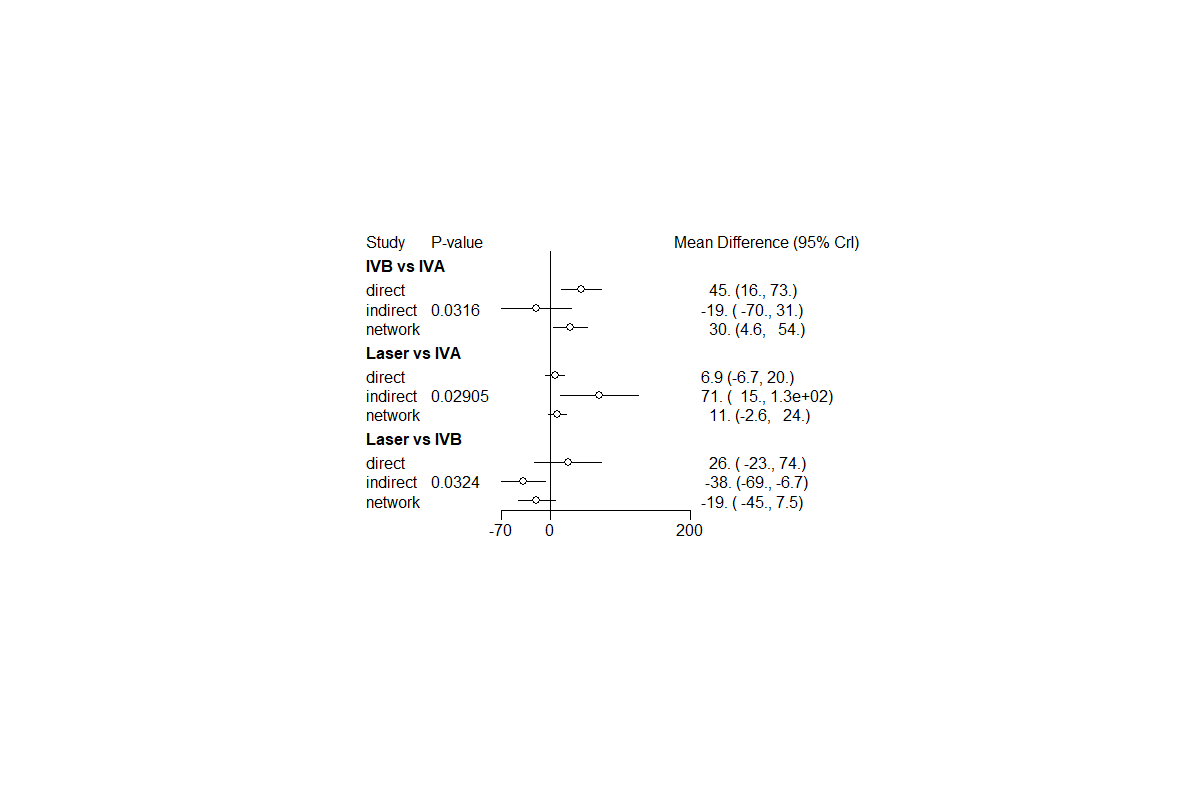


- 1. **Trace plot and density plot**

PSRF=1.000399.


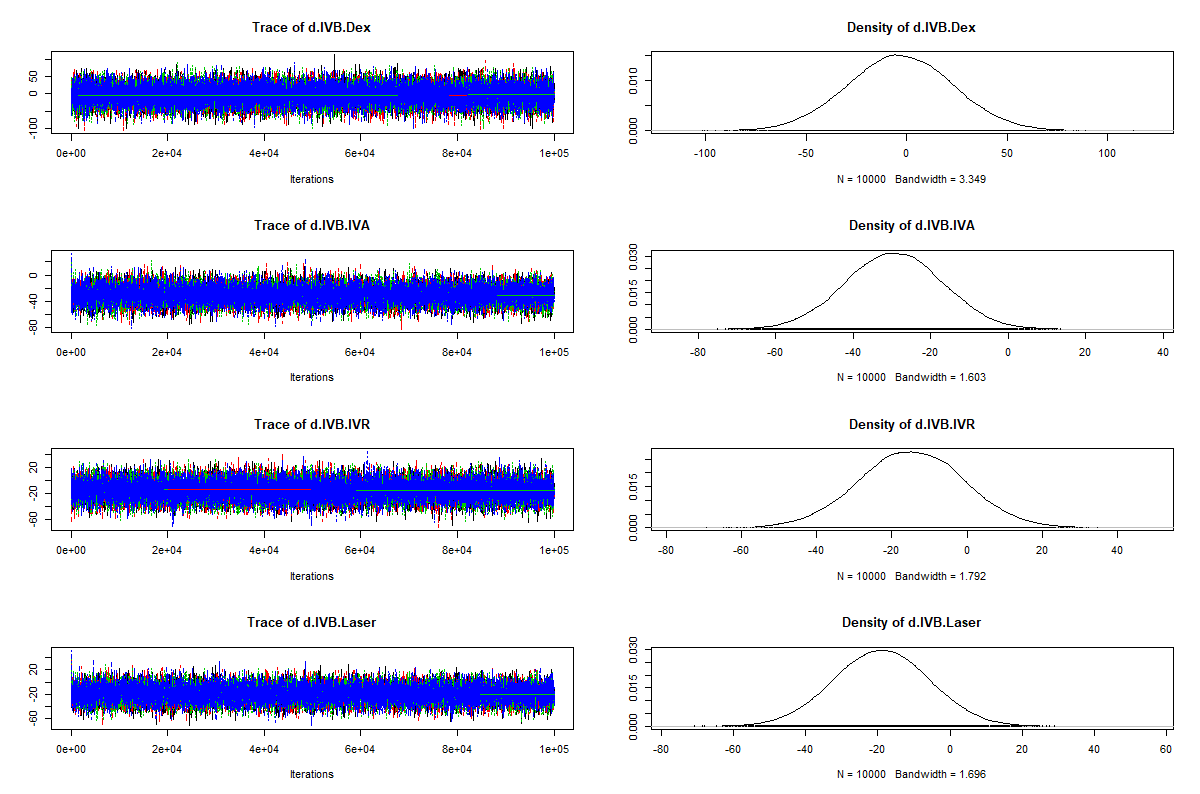


1. **Population with worse baseline VA at 1-year follow-up**
   1. **Node-split plot**

- 1. **Trace plot and density plot**

PSRF=1.000387.

## Adverse events

1. **Serious adverse events in all population at 1-year follow-up**
   1. **Node-split plot**

- 1. **Trace plot and density plot**

PSRF=1.000577.

1. **Ocular adverse events in all population at 1-year follow-up**
   1. **Node-split plot**

Node-split plot was not got due to lacking closed loop.

- 1. **Trace plot and density plot**

PSRF=1.000281.
